# Induction of Paraptotic Cell Death in Cancer Cells by Triptycene–Peptide Hybrids and the Revised Mechanism of Paraptosis II

**DOI:** 10.1021/acs.biochem.4c00085

**Published:** 2024-08-14

**Authors:** Mayuka Nii, Kohei Yamaguchi, Toshifumi Tojo, Nozomi Narushima, Shin Aoki

**Affiliations:** † Faculty of Pharmaceutical Sciences, Tokyo University of Science, 2641 Yamazaki, Noda 278-8510, Japan; ‡ Research Institute for Science and Technology (RIST), Tokyo University of Science, 2641 Yamazaki, Noda, Chiba 278-8510, Japan; § Research Institute for Biomedical Sciences (RIBS), Tokyo University of Science, 2641 Yamazaki, Noda, Chiba 278-8510, Japan

## Abstract

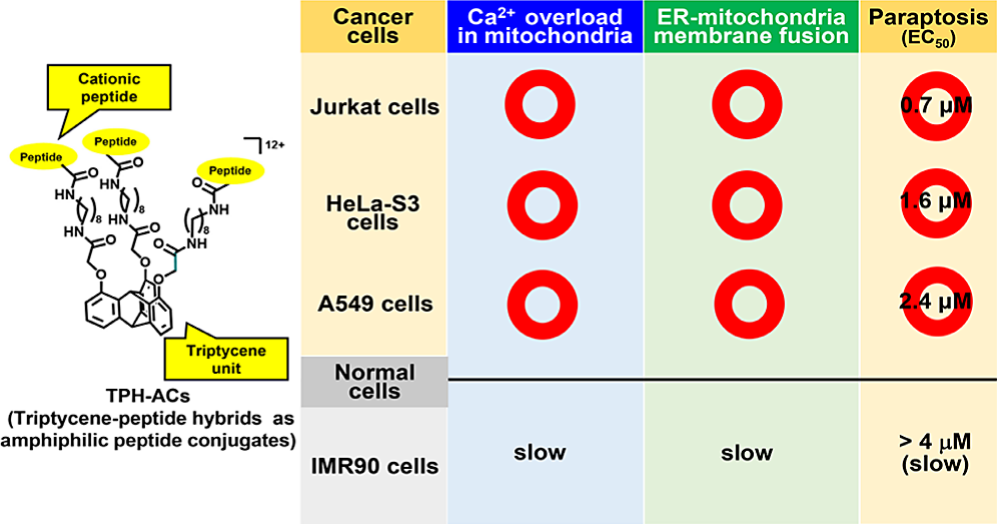

In previous work, we reported on iridium(III) (Ir(III)) complex-peptide hybrids as amphiphilic conjugates (IPH-ACs) and triptycene-peptide hybrids as amphiphilic conjugates (TPH-ACs) and found that these hybrid compounds containing three cationic KK(K)GG peptide units through C_6_–C_8_ alkyl linkers induce paraptosis II, which is one of the nonapoptotic programmed cell death (PCD) types in Jurkat cells and different from previously reported paraptosis. The details of that study revealed that the paraptosis II induced by IPH-ACs (and TPH-ACs) proceeds via a membrane fusion or tethering of the endoplasmic reticulum (ER) and mitochondria, and Ca^2+^ transfer from the ER to mitochondria, which results in a loss of mitochondrial membrane potential (*ΔΨ*_m_) in Jurkat cells. However, the detailed mechanistic studies of paraptosis II have been conducted only in Jurkat cells. In the present work, we decided to conduct mechanistic studies of paraptosis II in HeLa-S3 and A549 cells as well as in Jurkat cells to study the general mechanism of paraptosis II. Simultaneously, we designed and synthesized new TPH-ACs functionalized with peptides that contain cyclohexylalanine, which had been reported to enhance the localization of peptides to mitochondria. We found that TPH-ACs containing cyclohexylalanine promote paraptosis II processes in Jurkat, HeLa-S3 and A549 cells. The results of the experiments using fluorescence Ca^2+^ probes in mitochondria and cytosol, fluorescence staining agents of mitochondria and the ER, and inhibitors of paraptosis II suggest that TPH-ACs induce Ca^2+^ increase in mitochondria and the membrane fusion between the ER and mitochondria almost simultaneously, suggesting that our previous hypothesis on the mechanism of paraptosis II should be revised.

## Introduction

Programmed cell death (PCD) is a suicide process in which unnecessary cells undergo genetically controlled programs classified by terms such as apoptosis, necroptosis, paraptosis, autophagy, and ferroptosis.^1−8^ Although many types of anticancer drugs are used in hospitals, there is a need for the development of anticancer drugs with various mechanisms of action to avoid drug resistance against specific anticancer agents. Paraptosis was first reported by Sperandio et al. as nonapoptotic PCD characterized by cytoplasmic vacuolization and mitochondrial swelling with neither DNA fragmentation nor the effect of apoptosis inhibitors.^9−11^ Later, it was reported that paraptosis is induced in breast cancer cell lines (MDA-MB-435S and MCF-7) and colorectal cancer cell lines (DLD-1 and RKO) by celastrol,^12,13^ in MDA-MB-231 and in MDA-MB-435S by curcumin derivatives,^14−16^ and in ovarian cancer cells (A2780, SK-OV-3 and HO-8910 cells) by morucin.^17^ However, many aspects of paraptosis are poorly understood and a deep understanding of this process could lead to new strategies for the treatment of conditions such as cancers, autoimmune diseases, and viral infections.

**Scheme 1:**
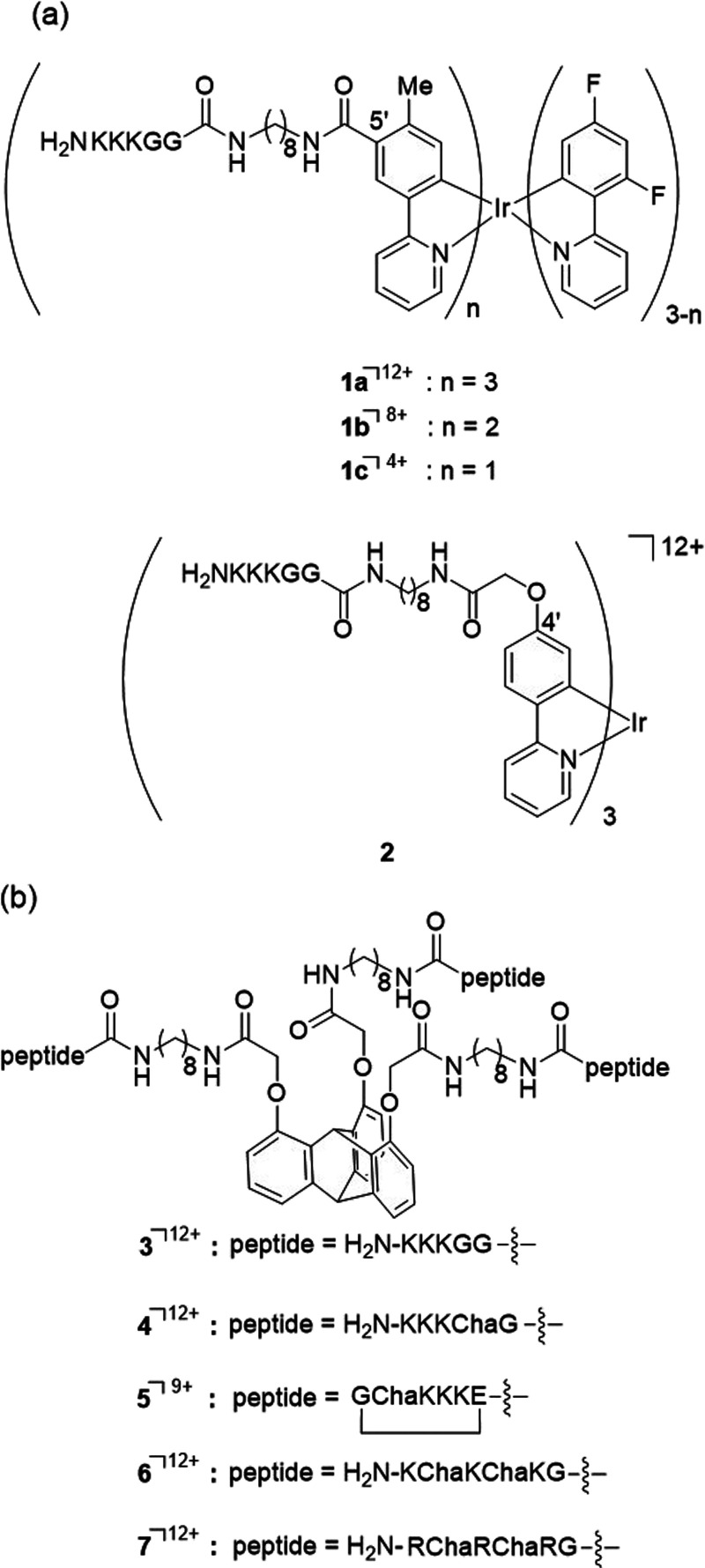
Chemical Structures of Ir(III) Complexes and Triptycene-Peptide Hybrids as Amphiphilic Peptide Conjugates (IPH-ACs (a) and TPH-ACs (b)) (G: glycine, K: l-lysine, and Cha: l-cyclohexylalanine)

We recently reported on the design and synthesis of hybrid compounds of iridium(III) (Ir(III)) complex^18−25^ and triptycene (9,10-dihydro-9,10[1′,2’]-benzenoanthracene)^26^ scaffolds with cationic peptides such as KK(K)GG (K: l-lysine; G: glycine) through C_6_–C_8_ alkyl linkers (Scheme 1). In that study, we found that Ir(III) complex-peptide hybrids and triptycene-peptide hybrids as amphiphilic conjugates (IPH-ACs (such as **1** and **2**) and TPH-ACs (such as **3**), respectively) induce paraptosis in Jurkat (T-lymphocyte leukemia) cells in several ways: membrane fusion or tethering between the endoplasmic reticulum (ER) and mitochondria; the transfer of Ca^2+^ from the ER to mitochondria; a decrease in mitochondrial membrane potential (*ΔΨ*_m_); and, vacuolization of intracellular organelles.

The cytotoxicity of **1a** was found to be more potent than that of either **1b** or **1c**, suggesting that the cytotoxicity of **1a**–**1c** is dependent on the number of cationic peptide units and their net cationic charge.^24^ These results suggest that IPH-ACs and TPH-AC may represent new types of anticancer drugs and that unraveling the induction mechanism of paraptosis would contribute to the development of novel anticancer agents.

**Scheme 2:**
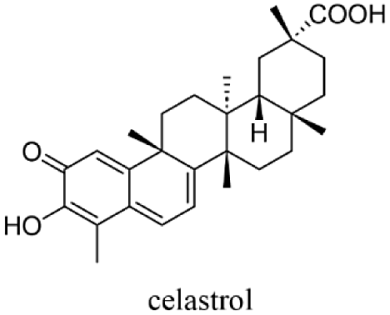
Structure of Celastrol

A more detailed mechanistic study of paraptosis induced by **1**–**3** in Jurkat cells was carried out^22−26^ and compared with that induced by celastrol (Scheme 2), which had been reported as a naturally occurring triterpenoid and a paraptosis inducer.^12,13^ Due to the observation of cell death induced by celastrol and IPH-ACs (TPH-ACs), cell death induced by celastrol was classified as paraptosis I, which negligibly involves membrane fusion between the ER and mitochondria, and cell death induced by IPH-ACs and TPH-ACs was referred to as paraptosis II, which is associated with membrane fusion between the ER and mitochondria.^25,26^ In addition, we found that TPH-ACs exhibit potent cytotoxicity against various cell lines like HeLa-S3 (human cervix carcinoma) and A549 (human Caucasian lung carcinoma) cells, but the cytotoxicity of IPH-ACs against HeLa-S3 and A549 cells is weak.

In this work, we decided to conduct mechanistic studies of the general mechanism of paraptosis II in HeLa-S3 and A549 cells as well as that in Jurkat cells. In addition, we designed and synthesized novel TPH-ACs **4**–**7** that possess linear or cyclic peptides containing cyclohexylalanine (Cha) in addition to K or arginine (R), because the localization of IPH-ACs in mitochondria had been observed in our previous works^25^ and it had been reported that Cha-containing peptides exhibit high localization in mitochondria.^27^ Besides, cyclic peptides exhibit higher stability, higher biological activity, higher cell membrane permeability and higher selectivity against target biomolecules than those of the corresponding linear peptide sequences.^28−30^ The results of the experiments described in this manuscript using fluorescence Ca^2+^ probes in mitochondria and cytosol (Rhod-2/AM and Rhod-4/AM, respectively), fluorescence probes of mitochondria and the ER (MitoTracker Green and ERTracker Red, respectively) suggest that the paraptosis II induced by TPH-ACs in Jurkat, HeLa-S3, and A549 cells proceeds via the Ca^2+^ transfer possibly from the ER to mitochondria and fusion (or tethering) between the ER and mitochondria almost simultaneously. In addition, the experiments using some inhibitors of Ca^2+^ transport system from the ER and mitochondria such as carbonyl cyanide 3-chlorophenylhydrazone (CCCP), which is an uncoupling reagent and an inhibitor of mitochondrial Ca^2+^ uptake and had been found to inhibit paraptosis II, 2-aminophenyl borate (2-APB), an antagonist of InsP_3_ (inositol 1,4,5-trisphosphate) receptor, and 4,4′-diisothiocyano-2,2′-stilbenedisulfonic acid (DIDS), which is an inhibitor of the voltage-dependent anion channel (VDAC), were carried out. As a result, we would like to add some revisions to our previous hypothesis in this manuscript.

Moreover, the cytotoxicity of TPH-ACs against IMR90 cells, a model of normal cells, is reported to be weaker than that against the aforementioned three cancer cell lines. This result is attributed to a slower Ca^2+^ increase in mitochondria and a weaker interaction between the ER and mitochondria than those in cancer cell lines even after treatment with **4**, as proven via costaining experiments using ERTracker Red and MitoTracker Green. The results are reported herein.

## Experimental Section

### General Information

All reagents and solvents were of the highest commercial quality available and used without further purification. Anhydrous *N,N*-dimethylformamide (DMF) was obtained by distillation from calcium hydride. All aqueous solutions were prepared using deionized, distilled water. 3-(4,5-Dimethylthiazol-2-yl)-2,5-diphenyl-2*H*-tetrazolium bromide (MTT), Rhod-2/AM, Rhod-4/AM, zinquin ethyl ester and Mito-FerroGreen were purchased from Dojindo (Kumamoto, Japan). Cisplatin was purchased from Tokyo Chemical Industry (Tokyo, Japan). Z-VAD-fmk was purchased from the Peptide Institute (Osaka, Japan), and necrostatin-1 was purchased from Enzo Life Sciences (U.S.A.). Propidium iodide (PI), etoposide and 3-methyladenine (3-MA) were purchased from Fujifilm Wako Chemicals (Osaka, Japan). Carbonyl cyanide 3-chlorophenylhydrazone (CCCP), trifluoromethoxy carbonylcyanide phenylhydrazone (FCCP), 1,1′,3,3,3′,3′-hexamethylindodicarbocyanine (DilC1(5)), 4,4′-diisothiocyano-2,2′-stilbenedisulfonic acid disodium salt (DIDS), adenosine 5′-triphosphate disodium salt hydrate (ATP), and adenosine 5′-diphosphate sodium salt (ADP) were purchased from Sigma-Aldrich (U.S.A.). MitoTracker Green, and ERTracker Red were purchased from Invitrogen. Roswell Park Memorial Institute (RPMI) 1640 medium, Minimum Essential Medium (MEM), Dulbecco’s modified Eagle’s medium (DMEM), phosphate-buffered saline (PBS), sodium dodecyl sulfate (SDS), glycine, sodium chloride, RuRed, D-PBS(−), accutase and trypsin from porcine pancreas were purchased from Nacalai Tesque (Kyoto, Japan). 2-APB was purchased from Cayman Chemical Co (U.S.A.). Celastrol was purchased from Toronto Research Chemicals (Canada). Fetal bovine serum (FBS) was purchased from Capricorn products, Inc. Concentrations of **3**, **4**, **5**, **6**, and **7** in stock solutions were determined based on elemental analysis. Stock solutions of **3**, **4**, **5**, H_2_N-KKKChaG peptide, cisplatin, 3-MA, DIDS and RuRed in PBS and **6**, **7**, etoposide, celastrol, Z-VAD-fmk, necrostatin-1, CCCP, FCCP, and 2-APB in DMSO were prepared for measurements and cell assays and were stored at 0 °C prior to use. IR spectra were measured with a PerkinElmer FT-IR spectrophotometer (Spectrum 100) at room temperature. Melting points were measured using a Yanaco MP-J3Micro Melting Point apparatus and are uncorrected. The ^1^H (300 and 400 MHz) NMR spectra were recorded on a JEOL 300 spectrometer and a JEOL 400 spectrometer, respectively. Tetramethylsilane (TMS) was used as an internal reference for ^1^H NMR measurements in CD_3_OD and DMSO-*d*_*6*_. Mass spectra measurements were performed using Sciex X500R QTOF (AB SCIEX, Framingham, MA) spectrometer. Elemental analyses were performed on a 2400 series II CHNS elemental analyzer (PerkinElmer, MA). Thin-layer chromatography (TLC) and silica gel column chromatography were performed using Merck Art. 5554 (silica gel) TLC plates and Fuji Silysia Chemical FL-100D, respectively. Gel permeation chromatography (GPC) experiments were performed on a Recycling Preparative GPC system (LaboACE LC-5060) equipped with a UV detector (Japan Analysis Industry Co., Ltd., Japan) and a gel permeation column (JAIGEL-2HR, 20 mm φ × 600 mm). HPLC experiments were carried out using a system consisting of a LC-NetII/ADC HPLC pump (JASCO, Japan), a UV-970 intelligent UV–visible detector (JASCO), a Rheodine injector (model no. 7125), and a ChromeNAV Lite. (JASCO). For analytical HPLC, a SenshuPak Pegasil ODS column (Senshu Scientific Co., Ltd.) (4.6 *ϕ* × 250 mm, no. 2012141S) was used. The results of MTT assays and experiments of fluorescence changes in Rhod-2/AM and Rhod-4/AM were confirmed using a multilabel counter, the Wallac 1420 ARVO (PerkinElmer). Fluorescence imaging studies were performed using fluorescence microscopy (Biorevo, BZ-X800, Keyence; and FluoView, FV-1000, Olympus). Flow cytometric analyses were performed using a flow cytometer (FACS Calibur cytometer, Becton), and data were analyzed using the FlowJo software (FlowJo, LCC).

### Synthesis

#### Protected Peptide **10** (Boc-K(Boc)-K(Boc)-K(Boc)-Cha-G-OH)

Fmoc-Gly-2-Trt-Resin (150 mg, 0.06 mmol) was deprotected by treatment with 20% piperidine/DMF. Each Fmoc-Xaa–OH (0.24 mmol) was coupled to the Fmoc-deprotected resin at room temperature for 1 h in the presence of *N,N*-diisopropylcarbodiimide (DIC) (36 μL, 0.23 mmol) and 1-hydroxybenzotriazole (HOBt) (32 mg, 0.23 mmol) in DMF (1.0 mL). The protected peptide was cleaved from the resin using a solution of 1,1,1,3,3,3-hexafluoropropan-2-ol (HFIP)/CH_2_Cl_2_ (60/40) by stirring for 4 h. After separating the resin by filtration and washing several times with CH_2_Cl_2_, the resultant filtrate was concentrated under reduced pressure. The residue was purified by silica gel column chromatography (CHCl_3_/MeOH = 10/1) to afford **10** (61 mg, quant.) as a colorless solid. Mp 160 °C (dec.). IR(ATR): ν = 3281, 2926, 2856, 2163, 1978, 1684, 1658, 1634, 1521, 1450, 1392, 1366, 1277, 1249, 1167, 1042, 1017, 866, 780, 652, 463, 423 cm^–1^. ^1^H NMR (400 MHz, DMSO-*d*_*6*_/TMS): δ = 8.01 (s, 1H), 7.93 (d, *J* = 7.2 Hz, 1H), 7.84 (d, *J* = 8.0 Hz, 1H), 7.76 (d, *J* = 7.6 Hz, 1H), 6.89 (d, *J* = 7.6 Hz, 1H), 6.74–6.73 (m, 3H), 4.37–4.31 (m, 1H), 4.23–4.17 (m, 2H), 3.85 (m, 1H), 3.75 (d, *J* = 5.6 Hz, 1H), 3.67 (d, *J* = 5.6 Hz, 1H), 2.86–2.85 (m, 7H), 1.70–1.61 (m, 7H), 1.52–1.45 (m, 2H), 1.38–1.34 (m, 49H), 1.23 (m, 4H), 1.11 (m, 1H), 0.89–0.86 (m, 2H) ppm. ESI/MS (*m*/*z*): calcd. for C_49_H_89_N_8_O_14_: [M + Na]^+^ 1013.6493; found 1013.6491.

#### Peptide **11**

A solution of **14** (329 mg, 0.29 mmol) in DMSO (20 mL) was dropped in a mixture of HBTU (440 mg, 1.17 mmol), HOBt (158 mg, 1.17 mmol), and *N,N*-diisopropylethylamine (200 μL, 1.16 mmol) in DMSO (4 mL) at 0 °C. After dropping, the reaction mixture was let stand at room temperature for 6 h. After the reaction, the residue was dried under reduced pressure and dissolved in CHCl_3_. The organic layer was washed with water, then dried over Na_2_SO_4_, filtered, and concentrated under reduced pressure. The residue was purified by GPC (CHCl_3_) to afford the intermediate as a yellow powder (139 mg, 43%). To a suspension of palladium on carbon (77 mg) in MeOH (2 mL) was added a solution of the intermediate (70 mg, 0.06 mmol) and CH_3_COOH (3 drops) in THF (6 mL). The mixture was stirred in the presence of H_2_ gas (1 atm) at room temperature for 6 h. The palladium on carbon was filtered using celite, and the resultant solution was concentrated under reduced pressure. The resultant residue was reprecipitated from CHCl_3_ and hexanes to afford **11** (57 mg, 89%) as a colorless solid. Mp 166–169 °C (dec.). IR(ATR): ν = 3298, 2928, 1647, 1515, 1450, 1392, 1366, 1248, 1167, 995, 862, 557, 463, 439, 427, 408 cm^–1^. ^1^H NMR (400 MHz, DMSO-*d*_6_/TMS): δ = 9.70 (d, *J* = 9.2 Hz, 1H), 9.40 (d, *J* = 7.6 Hz, 1H), 9.32 (d, *J* = 8.0 Hz,1H), 7.98 (d, *J* = 5.6 Hz, 1H), 7.73 (d, *J* = 10 Hz, 1H), 7.48 (m, 1H), 6.75 (s, 2H), 4.34–4.12 (m, 6H), 3.94–3.92 (m, 1H), 2.90–2.84 (m, 6H), 2.50 (s, 1H), 2.33–2.24 (m, 2H), 2.01–1.92 (m, 1H), 1.83–1.70 (m, 1H), 1.66–1.57 (m, 7H), 1.36 (s, 33H), 1.23–1.07 (m, 9H), 0.87–0.84 (m, 3H) ppm. ESI/MS (*m*/*z*): calcd. for C_49_H_89_N_10_O_14_: [M + NH_4_]^+^ 1041.6554; found 1041.6555.

#### Protected Peptide **12** (Boc-K(Boc)-Cha-K(Boc)-Cha-K(Boc)-G-OH)

Compound **12** was obtained as a colorless powder (164 mg, quant.) from Fmoc-Gly-2-Trt-Resin (300 mg, 0.14 mmol) and each Fmoc-Xaa–OH (0.56 mmol) in the presence of DIC (86 μL, 0.56 mmol) and HOBt (75 mg, 0.56 mmol) using a procedure similar to that for **10** (1 mL of DMF was used as the solvent). Mp 235 °C (dec.). IR(ATR): ν = 3267, 2926, 2161, 2020, 1690, 1625, 1521, 1451, 1392, 1366, 1250, 1166, 1002, 863, 698, 460, 428 cm^–1^. ^1^H NMR (400 MHz, DMSO-*d*_*6*_/TMS): δ = 8.12 (s, 1H), 7.93 (d, *J* = 7.2 Hz, 1H), 7.84 (d, *J* = 7.6 Hz, 1H), 7.76 (m, 1H), 6.89 (d, *J* = 7.2 Hz, 1H), 6.75–6.72 (m, 3H), 4.35–4.31 (m, 2H), 4.23–4.19 (m, 2H), 3.85–3.84 (m, 1H), 3.76–3.75 (m, 1H), 3.73–3.71 (m, 1H), 2.88–2.83 (m, 7H), 1.70–1.61 (m, 10H), 1.51–1.41 (m, 2H), 1.38–1.36 (m, 48H), 1.23–1.17 (m, 2H), 1.11–1.07 (m, 3H), 0.86–0.80 (m, 4H) ppm. ESI/MS (*m*/*z*): calcd. for C_58_H_103_N_9_NaO_15_: [M + Na]^+^ 1188.7466; found 1188.7466.

#### Protected Peptide **13** (Boc-R(Boc)-Cha-R(Pbf)-Cha-R(Pbf)-G-OH)

Fmoc-Gly-2-Trt-Resin (318 mg, 0.17 mmol) was deprotected by treatment with 20% piperidine/DMF. Each Fmoc-Xaa–OH (0.67 mmol) was coupled to the Fmoc-deprotected resin at room temperature for 1 h in the presence of DIC (104 μL, 0.67 mmol) and HOBt (91 mg, 0.67 mmol) in DMF (1.0 mL). The protected peptide was cleaved from the resin using a solution of HFIP/CH_2_Cl_2_ (60/40) by stirring for 4 h. After separating the resin by filtration and washing several times with CH_2_Cl_2_, the resultant filtrate was concentrated under reduced pressure. The resultant residue was reprecipitated from CHCl_3_/MeOH = 10/1 and Hexanes to afford **13** (212 mg, 81%) as a colorless powder. Mp 187 °C (dec.). IR(ATR): ν = 3282, 2924, 2851, 2162, 1977, 1625, 1546, 1449, 1394, 1369, 1252, 1152, 1091, 1033, 900, 852, 808, 783, 661, 641, 619, 567, 507, 451, 418 cm^–1^. ^1^H NMR (400 MHz, DMSO-*d*_*6*_/TMS): δ = 7.95 (m, 1H), 7.89–7.87 (m, 1H), 6.39 (s, 1H), 4.36–4.25 (m, 2H), 4.16 (s, 1H), 3.91–3.89 (m, 1H), 3.49 (s, 2H), 3.08–3.02 (m, 4H), 2.96 (s, 3H), 2.73–2.65 (m, 1H), 2.42 (m, 5H), 2.33 (m, 1H), 2.00 (s, 5H), 1.60 (s, 2H), 1.48–1.47 (m, 3H), 1.44 (s, 4H), 1.37 (s, 26H), 1.10 (m, 5H), 0.86 (m, 4H) ppm. ESI/MS (*m*/*z*): calcd. for C_74_H_121_N_15_O_17_S_2_: [M + 2H]^2+^ 777.9248; found 777.9250.

#### Protected Peptide **14** (H_2_N–K(Boc)-K(Boc)-K(Boc)-Cha-E(Bn)-G-OH)

Fmoc-Gly-2-Trt-Resin (300 mg, 0.12 mmol) was deprotected by treatment with 20% piperidine/DMF. Each Fmoc-Xaa–OH (0.48 mmol) was coupled to the Fmoc-deprotected resin at room temperature for 1 h in the presence of DIC (73 μL, 0.48 mmol) and HOBt (64 mg, 0.48 mmol) in DMF (1.0 mL). The protected peptide was cleaved from the resin using a solution of HFIP/CH_2_Cl_2_ (60/40) by stirring for 4 h. After separating the resin by filtration and washing several times with CH_2_Cl_2_, the resultant filtrate was concentrated under reduced pressure. The resultant residue was reprecipitated from DMSO and H_2_O to afford **14** (127 mg, 96%) as a colorless powder. Mp 239 °C (dec.). IR(ATR): ν = 3281, 2923, 2853, 2161, 1979, 1689, 1632, 1516, 1448, 1391, 1366, 1248, 1166, 1013, 864, 780, 502, 476, 459, 430, 419, 406 cm^–1^. ^1^H NMR (300 MHz, DMSO-*d*_*6*_/TMS): δ = 8.08 (d, *J* = 7.8 Hz, 1H), 7.91–7.83 (m, 3H), 7.35 (s, 5H), 6.80–6.64 (m, 3H), 5.08 (s, 2H), 4.32–4.15 (m, 4H), 2.85 (t, *J* = 6.3 Hz, 7H), 2.73 (t, *J* = 2.1 Hz, 1H), 1.60 (m, 7H), 1.47 (m, 10H), 1.37–1.12 (m, 49H), 0.85–0.81 (m, 2H) ppm. ESI/MS (*m*/*z*): calcd. for C_56_H_94_N_9_O_15_: [M + Na]^+^ 1132.6864; found 1132.6864.

#### Triptycene-Peptide Hybrid **4**

To a solution of **9**^26^ (25 mg, 0.03 mmol) in distilled DMF (0.8 mL) was added PyBOP (82 mg, 0.16 mmol), *N,N*-diisopropylethylamine (27 μL, 0.16 mmol), and the protected peptide (KKKChaG) **10** (137 mg, 0.14 mmol). The reaction mixture was stirred at room temperature for 19 h. After the reaction, the residue was dried under reduced pressure and dissolved in CHCl_3_. The organic layer was washed with water, then dried over Na_2_SO_4_, filtered, and concentrated under reduced pressure. The residue was purified by GPC (CHCl_3_) to afford the protected compound. To a solution of the protected compound in CH_2_Cl_2_ (4 mL) was added TFA (4 mL), and the reaction mixture was stirred at room temperature for 5 h. The solution was evaporated and dried under reduced pressure. The residue was purified by RP-HPLC (CH_3_CN (0.1% TFA)/H_2_O (0.1% TFA) = 5/95 to 65/35 (30 min), t_τ_ = 23 min, 1.0 mL/min) and lyophilized to afford **4** as a colorless solid (42 mg, 27% as TFA salt). Mp 135 °C (dec.). IR(ATR): ν = 2928, 1659, 1538, 1179, 1129, 837, 799, 722, 516, 459, 413 cm^–1^. ^1^H NMR (400 MHz, CD_3_OD/TMS): δ = 7.13 (d, *J* = 7.6 Hz, 3H), 6.96 (t, *J* = 8.0 Hz, 3H), 6.92 (s, 1H), 6.64 (d, *J* = 8.0 Hz, 3H), 5.55 (s, 1H), 5.03 (s, 4H), 4.61 (s, 6H), 4.35–4.33 (m, 8H), 3.84–3.81 (m, 6H), 3.49–3.47 (m, 4H), 3.46–3.45 (m, 4H), 3.17–3.12 (m, 12H), 2.96–2.91 (m, 21H), 1.86–1.79 (m, 4H), 1.72–1.66 (m, 35H), 1.50–1.44 (m, 31H), 1.29–1.21 (m, 38H), 0.90 (m, 10H) ppm. ESI/MS (*m*/*z*): calcd. for C_137_H_242_N_30_O_21_: [M + 6H]^6+^ 440.6460; found 440.6459. C_137_H_248_N_30_O_21_^12+^·12TFA^–^· 5TFA·14H_2_O: calcd. for C 42.03, H 5.75, N 8.50; found C 42.08, H 5.45, N 8.61.

#### Triptycene-Peptide Hybrid **5**

To a solution of **9**^26^ (16 mg, 0.02 mmol) in DMSO (1.2 mL) was added HBTU (109 mg, 0.29 mmol), HOBt (42 mg, 0.31 mmol), *N,N*-diisopropylethylamine (49 μL, 0.28 mmol), and the protected cyclic peptide **11** (72 mg, 0.07 mmol). The reaction mixture was stirred at room temperature for 18 h. After the reaction, the residue was dried under reduced pressure and dissolved in CHCl_3_. The organic layer was washed with water, then dried over Na_2_SO_4_, filtered, and concentrated under reduced pressure. The residue was purified by silica gel column chromatography (CHCl_3_/MeOH = 30/1) to afford the protected compound. To a solution of the protected compound in CH_2_Cl_2_ (1.5 mL) was added TFA (1.5 mL), and the reaction mixture was stirred at room temperature for 5 h. The solution was evaporated and dried under reduced pressure. The residue was purified by RP-HPLC (CH_3_CN (0.1% TFA)/H_2_O (0.1% TFA) = 5/95 to 65/35 (30 min), t_τ_ = 27.4 min, 1.0 mL/min) and lyophilized to afford **5** as a colorless solid (17 mg, 35% as TFA salt). Mp 159 °C (dec.). IR(ATR): ν = 3283, 3061, 2928, 2856, 1643, 1532, 1439, 1261, 1199, 1178, 1129, 837, 800, 722, 518, 433 cm^–1^. ^1^H NMR (400 MHz, CD_3_OD/TMS): δ = 7.13 (d, *J* = 7.2 Hz, 3H), 6.96 (t, *J* = 8.0 Hz, 3H), 6.92 (s, 1H), 6.65 (d, *J* = 8.4 Hz, 3H), 5.55 (s, 1H), 5.02 (s, 1H), 4.61 (s, 6H), 4.59 (s, 6H), 4.36–4.22 (m, 12H), 4.13 (t, *J* = 6.8 Hz, 3H), 3.98 (s, 1H), 3.94 (s, 1H), 3.84 (s, 1H), 3.80 (s, 1H), 3.20–3.09 (m, 6H), 2.94–2.90 (m, 18H), 2.27–2.23 (m, 5H), 2.13–2.09 (m, 4H), 1.90–1.77 (m, 3H), 1.72–1.67 (m, 30H), 1.49–1.40 (m, 30H), 1.24 (s, 32H), 1.02–0.90 (m, 6H) ppm. ESI/MS (*m*/*z*): calcd. for C_152_H_256_N_33_O_27_: [M + 5H]^5+^ 595.1929; found 595.1924. C_152_H_260_N_33_O_27_^12+^·12TFA^–^· 3TFA·3H_2_O: calcd. for C 48.10, H 6.17, N 10.52; found C 48.19, H 6.11, N 10.57.

#### Triptycene-Peptide Hybrid **6**

To a solution of **9**^26^ (6.9 mg, 0.01 mmol) in distilled DMF (0.5 mL) was added PyBOP (18 mg, 0.04 mmol), *N,N*-diisopropylethylamine (9.8 μL, 0.04 mmol), and the protected peptide (KChaKChaKG) **12** (42 mg, 0.04 mmol). The reaction mixture was stirred at room temperature for 17 h. After the reaction, the residue was dried under reduced pressure and dissolved in CHCl_3_. The organic layer was washed with water, then dried over Na_2_SO_4_, filtered, and concentrated under reduced pressure. The residue was purified by silica gel column chromatography (CHCl_3_/MeOH = 30/1) to afford the protected compound. To a solution of the protected compound in CH_2_Cl_2_ (1 mL) was added TFA (1 mL), and the reaction mixture was stirred at room temperature for 5 h. The residue was purified by RP-HPLC (CH_3_CN (0.1% TFA)/H_2_O (0.1% TFA) = 5/95 to 65/35 (30 min), t_τ_ = 25.7 min, 1.0 mL/min) and lyophilized to afford **6** as a pale-yellow powder (9.8 mg, 47% as TFA salt). Mp 177 °C (dec.). IR(ATR): ν = 2925, 2853, 1635, 1533, 1447, 1200, 1179, 1130, 838, 800, 722, 518, 449, 419, 410 cm^–1^. ^1^H NMR (400 MHz, CD_3_OD/TMS): δ = 7.13 (d, *J* = 6.8 Hz, 3H), 6.96 (t, *J* = 8.4 Hz, 3H), 6.92 (s, 1H), 6.64 (d, *J* = 8.4 Hz, 3H), 5.55 (s, 1H), 5.01 (s, 6H), 4.61 (s, 6H), 4.41–4.37 (m, 8H), 4.28–4.20 (m, 2H), 3.84–3.83 (m, 6H), 3.48–3.47 (m, 3H), 3.46–3.45 (m, 2H), 3.17–3.12 (m, 8H), 2.96–2.91 (m, 21H), 1.86–1.60 (m, 85H), 1.49–1.47 (m, 34H), 1.29–1.21 (m, 49H), 0.99–0.92 (m, 17H) ppm. ESI/MS (*m*/*z*): calcd. for C_164_H_287_N_33_O_24_: [M + 6H]^6+^ 517.2036; found 517.2028. C_164_H_293_N_33_O_24_^12+^·12TFA^–^· 7TFA·3H_2_O: calcd. for C 45.61, H 5.80, N 8.69; found C 45.43, H 5.82, N 8.89.

#### Triptycene-Peptide Hybrid **7**

To a solution of **9**^26^ (25 mg, 0.03 mmol) in distilled DMF (2 mL) was added PyBOP (106 mg, 0.20 mmol), *N,N*-diisopropylethylamine (36 μL, 0.20 mmol), and the protected peptide (RChaRChaRG) **13** (292 mg, 0.19 mmol). The reaction mixture was stirred at room temperature for 14 h. After the reaction, the residue was dried under reduced pressure and dissolved in CHCl_3_. The organic layer was washed with water, then dried over Na_2_SO_4_, filtered, and concentrated under reduced pressure. The residue was purified by silica gel column chromatography (CHCl_3_/MeOH = 30/1) to afford the protected compound. To a solution of the protected compound in CH_2_Cl_2_ (1.5 mL) was added TFA (4.5 mL), and the reaction mixture was stirred at room temperature for 7 h. The solution was evaporated and dried under reduced pressure. The residue was purified by RP-HPLC (CH_3_CN (0.1% TFA)/H_2_O (0.1% TFA) = 5/95 to 65/35 (30 min), t_τ_ = 26.2 min, 1.0 mL/min) and lyophilized to afford **7** as a colorless solid (7.0 mg, 12% as TFA salt). Mp 147 °C (dec.). IR(ATR): ν = 3284, 2927, 2855, 1631, 1537, 1479, 1439, 1262, 1200, 1180, 1130, 839, 800, 722, 518, 465, 441, 416, 407 cm^–1^. ^1^H NMR (400 MHz, CD_3_OD/TMS): δ = 7.13 (d, *J* = 7.6 Hz, 3H), 6.96 (t, *J* = 7.6 Hz, 3H), 6.92 (s, 1H), 6.64 (d, *J* = 8.4 Hz, 3H), 5.55 (s, 1H), 5.02 (s, 7H), 4.61 (s, 11H), 4.42–4.38 (m, 10H), 4.33–4.29 (m, 3H), 3.89–3.79 (m, 8H), 3.49–3.46 (m, 12H), 3.20–3.17 (m, 20H), 3.15–3.14 (m, 5H), 1.96–1.60 (m, 92H), 1.49–1.44 (m, 10H), 1.29–1.21 (m, 59H), 1.02–0.90 (m, 18H) ppm. ESI/MS (*m*/*z*): calcd. for C_164_H_287_N_51_O_24_: [M + 6H]^6+^ 559.2129; found 559.2132. C_164_H_293_N_51_O_24_^12+^·12TFA^–^·14H_2_O: calcd. for C 40.39, H 5.39, N 11.44; found C 40.19, H 5.24, N 11.70.

### Stability of 4 and 5 After Treatment with Trypsin

**4** and **5** (10 μM) were incubated with trypsin (5 U/mL) in 10 mM 2-[4-(2-hydroxyethyl)-1-piperazinyl]ethanesulfonic acid ((HEPES), pH = 7.5) for 1 h at 37 °C and then analyzed via RP-HPLC (CH_3_CN (0.1% TFA)/H_2_O (0.1% TFA) = 20/80 to 70/30 (30 min), 1.0 mL/min).

### Cell Cultures

Jurkat cells (T-lymphocyte leukemia) were cultured in RPMI 1640 medium supplemented with 10% heat-inactivated fetal bovine serum (FBS), l-glutamine, (2-[4-(2-hydroxyethyl)-1-piperazinyl]ethanesulfonic acid (HEPES), pH = 7.5), penicillin, and streptomycin. HeLa S3 cells (human cervical carcinoma) were cultured in Minimum essential medium (MEM) containing 10% FBS, penicillin, and streptomycin. A549 cells (human Caucasian lung carcinoma) and IMR90 cells (human Caucasian fetal lung fibroblast) were cultured in Dulbecco’s modified Eagle’s medium (DMEM) with 10% FBS, penicillin, and streptomycin. All cells were cultured at 37 °C under a humidified atmosphere containing 5% CO_2_.

### PI Stain Assay of Jurkat, HeLa-S3, and A549 Cells After Treatment with 3, 4, 5, 6, 7, Etoposide, Cisplatin, and Celastrol

(a) Jurkat cells: Jurkat cells (2.0 × 10^5^ cells) were treated with **3** (5 μM, 100 μL), **4** (2.5 μM, 100 μL), **5** (5 μM, 100 μL), **6** (5 μM, 100 μL), and **7** (5 μM, 100 μL) for 1 h, and with etoposide (5 μM, 100 μL) and cisplatin (100 μM, 100 μL) for 24 h under 5% CO_2_ at 37 °C. After incubation, the cells were centrifuged at 2000 rpm at 4 °C for 3 min and then the resultant supernatant was discarded and washed with PBS. Propidium iodide (PI) (1 μg/mL, 100 μL) was added to the cells and the cell suspension was incubated for 15 min under the same conditions. The cells were centrifuged at 2000 rpm at 4 °C for 3 min and then washed with PBS. The cells were observed by fluorescence microscopy (BZ-X800, Keyence) using a Greiner CELLview Petri dish (35 × 10 mm^2^). Emission images were observed using a TRITC filter (excitation 540 nm, emission 605 nm).

(b) HeLa-S3, A549 and IMR90 cells: The cells were seeded on a Greiner CELLview Petri dish (35 × 10 mm^2^) in cell culture medium. After incubation overnight under 5% CO_2_ at 37 °C, the cells were treated with **3**–**7** (100 μL) for 1 or 2 h, and cisplatin (100 μM, 100 μL) and celastrol (100 μM, 100 μL) for 24 h under 5% CO_2_ at 37 °C. After incubation, the supernatant was discarded and washed with PBS. Subsequent procedures were the same as those used for the Jurkat cells.

### MTT Assays

Jurkat, HeLa-S3, A549 cells (2.0 × 10^5^ cells/mL) and IMR90 cells (1.0 × 10^5^ cells/mL) were incubated in the presence of **3**–**7** for 1 h, and etoposide, cisplatin and celastrol for 24 h (50 μL) in cell culture medium under CO_2_ at 37 °C in 96-well plates (BD Falcon). MTT reagent (5 mg/mL) in PBS (10 μL) was then added to the cells. After incubation at 37 °C for 4 h, a formazan lysis solution (10% sodium dodecyl sulfate (SDS) in 0.01 N HCl) (100 μL) was added and the resultant solution was incubated overnight under the same conditions, which was followed by measurement of the absorbance at 570 nm using a microplate reader (ARVO, PerkinElmer).

### MTT Assays of Jurkat, HeLa-S3, A549 Cells After Treatment with 4 and Celastrol in the Presence of Inhibitors (Z-VAD-fmk, Necrostatin-1, 3-Methyladenine, CCCP, FCCP, DIDS, 2-APB, and RuRed)

(a) Jurkat cells: Jurkat cells (2.0 × 10^5^ cells/mL) in RPMI 1640 medium with 10% FBS were pretreated with the inhibitors (Z-VAD-fmk (final concentration = 15 μM), necrostatin-1 (final concentration = 30 μM), 3-MA (final concentration = 10 μM), and DIDS (75 or 100 μM)) for 3 h or pretreated with other inhibitors (CCCP (40 μM), FCCP (40 μM), 2-APB (50 or 100 μM) and RuRed (75 μM)) for 1 h under 5% CO_2_ at 37 °C. After incubation, **4** (final concentration = 2.5 μM) was added to the cell suspension, which was then incubated for 1 h at 37 °C. To test the effect of celastrol, the cells were pretreated with the aforementioned inhibitors and then incubated with celastrol (5 μM) for 24 h. MTT reagent (5 mg/mL) in PBS (10 μL) was added to the cells. After incubation at 37 °C for 4 h, a formazan lysis solution (10% sodium dodecyl sulfate (SDS) in 0.01 N HCl) (100 μL) was added, and the resultant solution was incubated overnight under the same conditions, followed by measurement of the absorbance at 570 nm using a microplate reader (ARVO, PerkinElmer).

(b) HeLa-S3 cells: HeLa-S3 cells (2.0 × 10^5^ cells/mL) in MEM medium with 10% FBS were pretreated with the inhibitors (3-MA (5 mM), and DIDS (75 or 100 μM)) for 3 h or pretreated with other inhibitors (Z-VAD-fmk (100 μM), necrostatin-1 (100 μM), CCCP (160 μM), and FCCP (80 μM)) for 1 h under 5% CO_2_ at 37 °C. After the incubation, **4** (final concentration = 5 μM) was added to the cell suspension, which was then incubated for 1 h at 37 °C. Subsequent procedures were the same as those used for the Jurkat cells.

(c) A549 cells: A549 cells (2.0 × 10^5^ cells/mL) in DMEM medium with 10% FBS were pretreated with the inhibitors (3-MA (1 mM), and DIDS (250 μM)) for 3 h or pretreated with other inhibitors (Z-VAD-fmk (100 μM), necrostatin-1 (100 μM), CCCP (250 μM), and FCCP (10 μM)) for 1 h under 5% CO_2_ at 37 °C. After incubation, **4** (final concentration = 10 μM) was added to the cell suspension, which was then incubated for 1 h at 37 °C. Subsequent procedures were the same as those used for the Jurkat cells.

### Measurement of Mitochondrial Membrane Potential (*Δ*Ψ_m_)

Jurkat cells (1.2 × 10^5^ cells) were treated with 1,1’,3,3,3',3'-hexamethylindodicarbocyanine iodide (DilC1(5)) (500 nM) at 37 °C under 5% CO_2_ for 30 min. After Jurkat cells were washed with PBS, cells were incubated with **4** (2.5 μM) for 10–60 min under 5% CO_2_ at 37 °C and the time-dependent change of the emission from DilC1(5) was observed on fluorescence microscopy (BZ-X800, Keyence) (excitation wavelength for DilC1(5) was 635 nm). The time-dependent change of emission from DilC1(5) in Jurkat cells after the treatment with **4** in the presence of CCCP (40 μM) was measured by the pretreatment of Jurkat cells with CCCP for 1 h prior to the treatment with DilC1(5) and **4**.

### Flow Cytometry Analysis of Jurkat, HeLa-S3, and A549 Cells Stained with Rhod-2/AM or Rhod-4/AM

Jurkat, HeLa-S3, and A549 cells (2.0 × 10^5^ cells) were preincubated with Rhod-2/AM (final concentration = 5 μM) or Rhod-4/AM (final concentration = 5 μM) in medium under 5% CO_2_ at 37 °C for 30 min and then treated with **4** (final concentration = 2.5, 5, or 10 μM) for 0, 15, 30, 45, and 60 min. HeLa-S3 and A549 cells were detached by accutase, immediately after which the cells were suspended in 300 μL of cell culture medium and then analyzed on a flow cytometer (FACS Calibur cytometer, Becton), and the data were analyzed using the FlowJo software (FlowJo, LCC).

### Fluorescence Microscopic Observation of Jurkat, HeLa-S3, A549, and IMR90 Cells Treated with 4 and Stained Rhod-2/AM or Rhod-4/AM

Jurkat, HeLa-S3, A549, and IMR90 cells (2.0 × 10^5^ cells) were stained with Rhod-2/AM or Rhod-4/AM (final concentration = 5 μM) for 30 min at 37 °C under 5% CO_2_. The cells were washed with PBS and were treated with **4** (final concentration = 2.5, 5, or 10 μM) in cell culture medium for 30 and 60 min at 37 °C under 5% CO_2_. To test the effect of celastrol and cisplatin in HeLa-S3 and A549 cells, the cells were incubated with celastrol (final concentration = 100 μM) and cisplatin (final concentration = 100 μM) for 2–6 h. After washing with PBS, the cells were observed via fluorescence microscopy (BZ-X800, Keyence) using a Greiner CELLview Petri dish (35 × 10 mm^2^). Emission images were observed using a TRITC filter (excitation 540 nm, emission 605 nm).

### Observation of Time-Dependent Fluorescence Emission Changes of Rhod-2/AM or Rhod-4/AM After Treatment with 4 for 0–60 min

Jurkat, HeLa-S3, A549, and IMR90 cells (8.0 × 10^4^ cells) were incubated with CCCP or 2-APB for 1 h and then stained with Rhod-2/AM or Rhod-4/AM (final concentration = 5 μM) for 30 min at 37 °C under 5% CO_2_. The cells were washed with PBS and were treated with **4** (final concentration = 2.5, 5, or 10 μM) in PBS for 0–60 min at 37 °C in a microplate reader (ARVO, PerkinElmer). Excitation at 540 nm and emission at 590 nm were used for the measurement of Rhod-2/AM and Rhod-4/AM on the microplate reader.

### Observation of Intracellular (Mitochondria and the Endoplasmic Reticulum (ER)) in Jurkat, HeLa-S3, A549, and IMR90 Cells with 4 by Confocal Microscopy

Jurkat, HeLa-S3, A549, and IMR90 cells (2.0 × 10^5^ cells) were incubated with **4** (final concentration = 2.5 μM (for Jurkat), 5 μM (for HeLa-S3), 10 μM (for A549) and 5 μM and 50 μM (for IMR90)) in cell culture medium at 37 °C under 5% CO_2_. After incubation, MitoTracker Green (final concentration = 100 nM (for Jurkat) or 500 nM (for HeLa-S3, A549 and IMR90)), or ERTracker Red (final concentration = 500 nM (for Jurkat) or 1 μM (for HeLa-S3, A549 and IMR90)) were applied for 1 h under the same conditions. After washing with PBS, the cells were observed via confocal fluorescence microscopy (FluoView, FV-1000, Olympus). Excitation at 473 nm and emissions from 485 to 585 nm were used for detecting the MitoTracker Green. Excitation at 559 nm and emissions from 570 to 670 nm were used for detecting the ERTracker Red. Exposure time was 20 μs/pixel.

### MTT Assays of Jurkat Cells with 4 in the Presence of Zn(NO_3_)_2_

Jurkat cells (2.0 × 10^5^ cells) were incubated in RPMI 1640 medium containing solution of **4** (final concentration = 0.16–10 μM) with Zn(NO_3_)_2_ (final concentration = 15 μM) for 1 h. MTT reagent (5 mg/mL) in PBS (10 μL) was then added to the cells. After incubation at 37 °C for 4 h, a formazan lysis solution (10% sodium dodecyl sulfate (SDS) in 0.01 N HCl) (100 μL) was added and the resultant solution was incubated overnight under the same conditions, which was followed by measurement of the absorbance at 570 nm using a microplate reader (ARVO, PerkinElmer).

### Observation of Fe^2+^ and Zn^2+^ Ions in Jurkat Cells by Fluorescence Microscopy

Jurkat cells (2.0 × 10^5^ cells) were treated with **4** (2.5 μM), etoposide (5 μM), cisplatin (100 μM) and celastrol (5 μM) and stained with Mito-FerroGreen (a probe for labile Fe^2+^ in mitochondria) (5 μM) and zinquin (a probe for intracellular Zn^2+^) (25 μM). The cell images were observed via fluorescence microscopy.

### MTT Assays of Jurkat Cells with 4 in the Presence of ATP and ADP

Jurkat cells (2.0 × 10^5^ cells) were incubated in RPMI 1640 medium containing a solution of **4** (0.16–10 μM), etoposide (0.39–25 μM) and celastrol (0.16–10 μM) with ATP and ADP (100 μM) for 1 h. MTT reagent (5 mg/mL) in PBS (10 μL) was then added to the cells. After incubation at 37 °C for 4 h, a formazan lysis solution (10% sodium dodecyl sulfate (SDS) in 0.01 N HCl) (100 μL) was added and the resultant solution was incubated overnight under the same conditions, which was followed by measurement of the absorbance at 570 nm using a microplate reader (ARVO, PerkinElmer).

## Results and Discussion

### Design and Synthesis of Triptycene-Peptide Hybrids as Amphiphilic Peptide Conjugates (TPH-ACs) (4–7)

**Scheme 3:**
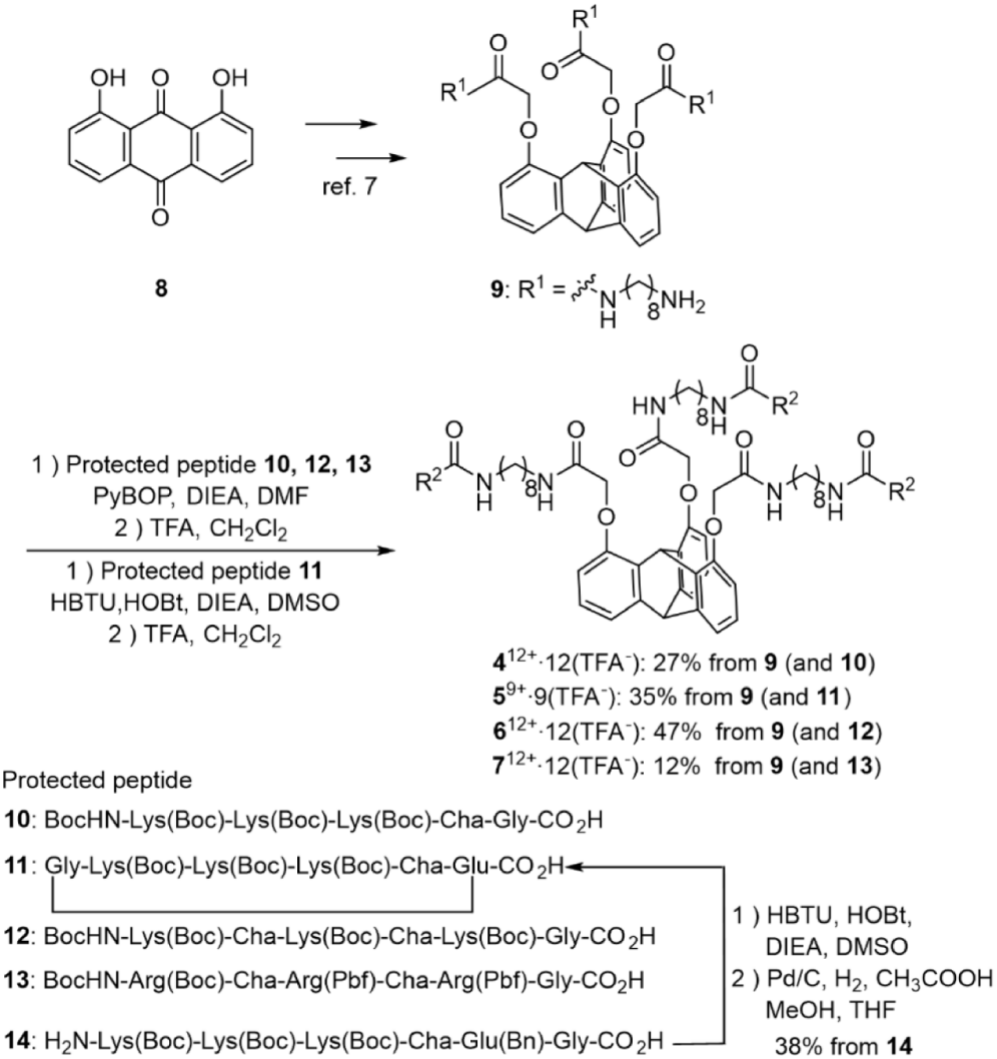
Synthesis of Triptycene-peptide Hybrids as Amphiphilic Conjugates (TPH-ACs) (**4–7**)

The synthesis of TPH-ACs (**4**–**7**) is shown in Scheme 3. The triptycene unit with three C_8_ linkers (**9**) was synthesized from **8** as described in our previous paper.^26^ The Boc (*tert*-butyloxycarbonyl) protected peptides **10**, **12**, and **13** were prepared by Fmoc solid-phase peptide synthesis and their coupling reactions with **9** were carried out using PyBOP (benzotriazole-1-yl-oxytripyrrolidinophosphonium hexafluorophosphate) and DIEA (*N,N*-diisopropylethylamine) in distilled DMF. Note that the coupling reaction of **9** with **11** was conducted by using HBTU (1-[bis(dimethylamino)methylene]-1*H*-benzotriazolium 3-oxide hexafluorophosphate), HOBt (1-hydroxybenzotriazole) and DIEA in DMSO. Finally, the deprotection of the protected groups in the corresponding intermediates by the treatment with TFA and purification by reversed-phase HPLC (RP-HPLC) afforded the TFA salts of **4**, **6** and **7**.

For the synthesis of **5**, the cyclization reaction of **14** was carried out between Lys(Boc) at the *N*-terminus and Gly at the *C*-terminus by using HBTU, HOBt, and DIEA followed by deprotection of the benzyl group via catalytic hydrogenations using H_2_, Pd/C, and CH_3_COOH in MeOH and THF to give cyclic peptide **11** without racemization during the cyclization reaction. Condensation reactions of **11** with **9** and successive deprotection afforded **5**.

We examined the stability of **4** containing linear peptides and **5** having the corresponding cyclic peptides against trypsin, which is a proteolytic enzyme that specifically hydrolyzes on the *C*-terminal side of R and K residues. We incubated **4** and **5** with trypsin (5 U/mL) for 1 h at 37 °C and analysis was conducted via RP-HPLC. As shown in Figure S1, considerable degradation of **4** was observed, while negligible decomposition of **5** was detected, which suggests a higher stability for **5** than that of **4** against trypsin digestion.

### Evaluation of the Cytotoxicity of TPH-ACs Against Jurkat, HeLa-S3, A549 Cells and Normal (IMR90) Cell Lines

**Figure 1:**
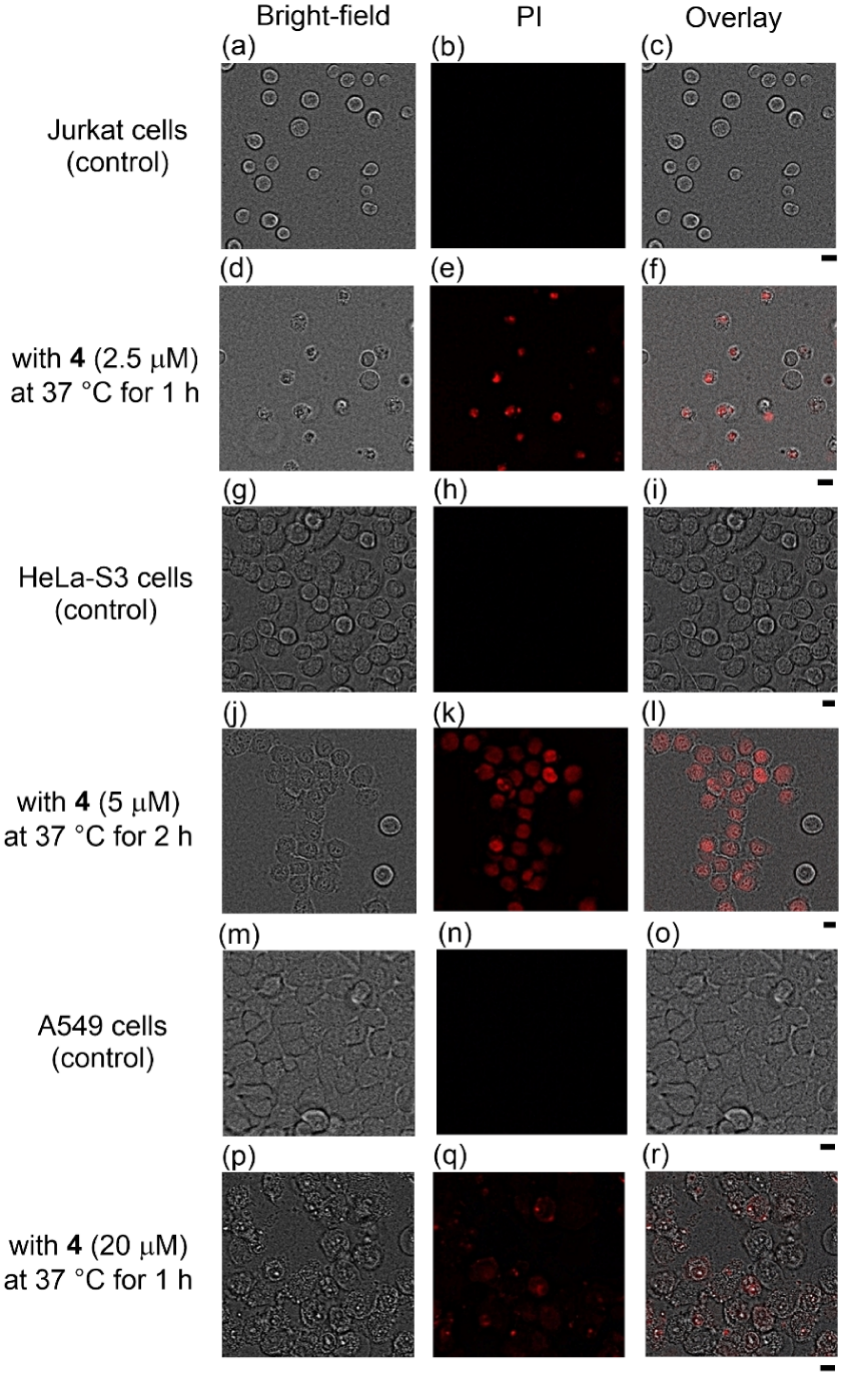
Fluorescence microscopic images of Jurkat (a-f), HeLa-S3 (g-l) and A549 cells (m-r) after treatment with **4** (2.5–20 μM) at 37 °C either for 1 or 2 h. (a) and (d) Bright-field images of Jurkat cells, (b) and (e) emission images of PI, (c) overlay images of (a) and (b), and (f) overlay images of (d) and (e). (g) and (j) Bright-field images of HeLa-S3 cells, (h) and (k) emission images of PI, and (i) overlay images of (g) and (h), and (l) overlay images of (j) and (k). (m) and (p) Bright-field images of A549 cells, (n) and (q) emission images of PI, (o) overlay images of (m) and (n), and (r) overlay images of (p) and (q). Excitation was at 540 nm for propidium iodide. Scale bar (black) is 10 μm.

The cytotoxicities of **3**, **4**, **5**, **6**, and **7** against Jurkat cells, HeLa-S3 cells, A549 cells, and IMR90 cells (human Caucasian fetal lung fibroblasts) was examined by using propidium iodide (PI), a fluorescent DNA intercalator and a detector of dead cells.^31^ The cells (2.0 × 10^5^ cells) were incubated with **3**–**7** for 1 or 2 h at 37 °C, which was followed by treatment with PI (1 μg/mL) for 15 min. As shown in Figure 1, cell death was observed in Jurkat, HeLa-S3, and A549 cells with similar morphological changes after incubation with **4** for 1 or 2 h at 37 °C under 5% CO_2_. Figures S2–S4 feature the fluorescence microscopic images of Jurkat, HeLa-S3, and A549 cells after treatment with **3** (5–25 μM), **4** (2.5–20 μM), **5** (5–25 μM), **6** (5–25 μM), and **7** (5–25 μM) at 37 °C for 1 or 2 h.

**Figure 2:**
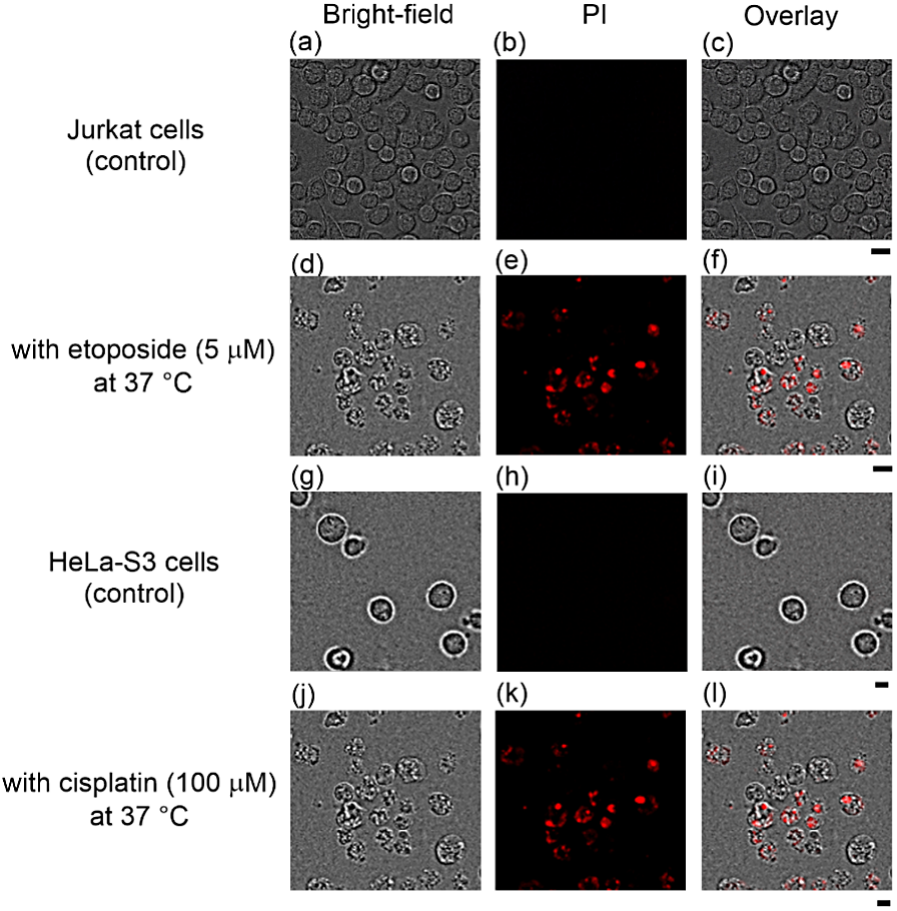
Fluorescence microscopic images of Jurkat cells treated with etoposide (5 μM), and HeLa-S3 cells treated with cisplatin (100 μM) at 37 °C for 24 h. (a) Bright-field, (b) emission, (c) overlay images of the control, (d) bright-field, (e) emission, and (f) overlay images with etoposide at 37 °C for 24 h, (g) bright-field, (h) emission, (i) overlay images of the control, (j) bright-field, (k) emission, and (l) overlay images with cisplatin at 37 °C for 24 h. Excitation was at 540 nm for propidium iodide. Scale bar (black) is 10 μm.

For comparison, morphological changes in Jurkat and HeLa-S3 cells after treatment with apoptosis inducers, etoposide (5 μM, 24 h) and cisplatin (100 μM, 24 h) (the structures of these compounds are shown in Chart S1), were observed by fluorescence microscopy after staining with PI (Figure 2). The results indicated that the morphological change was different from that induced by the TPH-ACs presented in Figures 1 and S2–S4.

Our previous work strongly indicated that paraptosis induced by celastrol in Jurkat cells involves negligible membrane fusion between the ER and mitochondria. In contrast, the paraptosis induced by TPH-ACs (and IPH-ACs) is characterized by membrane fusion between the ER and mitochondria, as previously reported.^25,26^ These facts allowed us to classify the examples of paraptosis induced by celastrol and TPH-ACs (IPH-ACs) as paraptosis I and paraptosis II, respectively. Morphological change in HeLa-S3 and A549 cells induced by celastrol (100 μM, 24 h) was observed using fluorescence microscopy. It should be noted that HeLa-S3 and A549 cells were detached from the well after treatment with celastrol, possibly because celastrol inhibits cell adhesion through the inhibition of VEGFR2 (vascular endothelial growth factor receptor 2), as described by Huan and Simons.^32,33^ Therefore, the detached cells in culture medium were collected by centrifugation, treated with PI, and then observed via microscopy (Figure S5).

**Figure 3:**
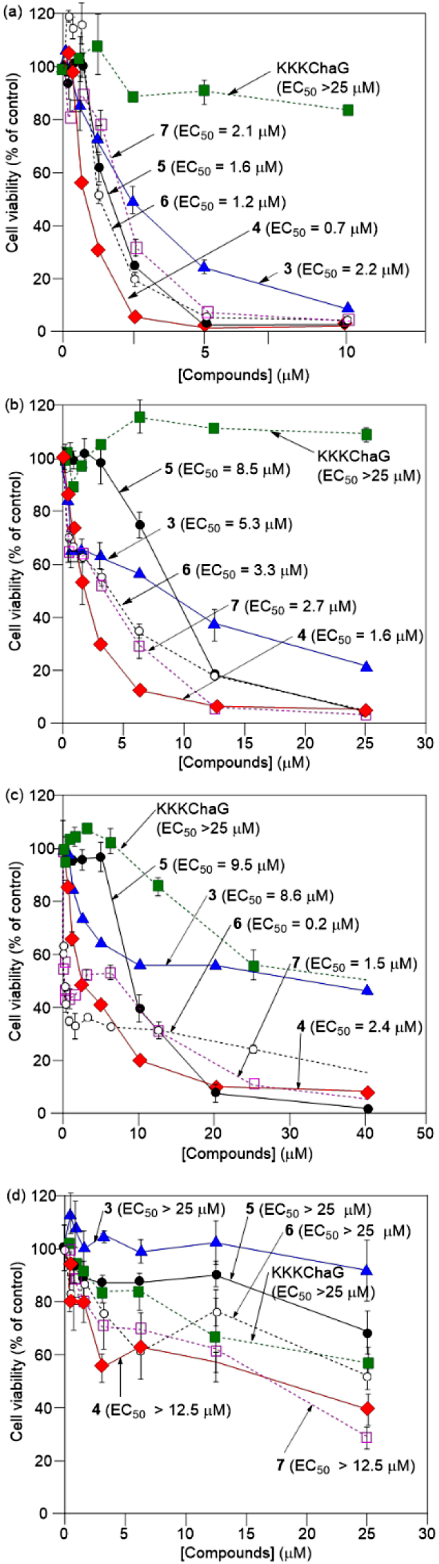
MTT assay results of Jurkat (a), HeLa-S3 (b), A549 (c), and IMR90 cells (d) after treatments with **3** (blue solid line, closed triangles), **4** (red solid line, closed diamonds), **5** (black solid line, closed circles), **6** (black dashed line, opened circles), **7** (pink dashed line, opened squares), and H_2_N-KKKChaG peptide (green dashed line, closed squares) at 37 °C for 1 h.

The cytotoxicity of TPH-ACs **3**–**7** against Jurkat, HeLa-S3, and A549 cells was evaluated via MTT (3-(4,5-dimethylthiazol-2-yl)-2,5-diphenyl-2*H*-tetrazolium bromide) assay. The cells (2.0 × 10^5^ cells/mL) were incubated with **3**–**7** in culture medium containing 10% fetal bovine serum (FBS) for 1 h at 37 °C under 5% CO_2_ and then treated with the MTT reagent and the results are summarized in Figure 3 and Table 1. The EC_50_ values of **3**–**7** were determined to be 0.7–2.2 μM against Jurkat cells, 1.6–8.5 μM against HeLa-S3 cells, and 0.2–9.5 μM against A549 cells, respectively, and it was found that **4** exhibits a higher cytotoxicity than **3** against these cell lines. For comparison, the EC_50_ values for **3**–**7** against IMR90 cells (human Caucasian fetal lung fibroblasts), a model of a normal cell line, were determined to be >12.5 μM under the incubation conditions described above (Figure 3d). The EC_50_ value of **4** against IMR90 cells was decreased to 4.4 μM after incubation for 5 h, suggesting slow induction of cell death in IMR90 cells by **4**.

**Table 1:** EC_50_ Values of **2**, **3**, **4**, **5, 6**, **7**, H_2_N-KKKChaG Peptide, Etoposide, Cisplatin, and Celastrol Against Jurkat, HeLa-S3, A549, and IMR90 Cells

compounds	Jurkat cells (μM)	HeLa-S3 cells (μM)	A549 cells (μM)	IMR90 cells (μM)
**2**	4.4 ± 0.1	6.5 ± 0.4	>25	>25
**3**	2.2 ± 0.5	5.3 ± 0.5	8.6 ± 1.8	>25
**4**	0.7 ± 0.1	1.6 ± 0.1	2.4 ± 0.1	>12.5 (4.4 μM)^a^
**5**	1.6 ± 0.1	8.5 ± 0.4	9.5 ± 0.1	>25
**6**	1.2 ± 0.1	3.3 ± 0.2	0.2 ± 0.4	>25
**7**	2.1 ± 0.1	2.7 ± 0.5	1.5 ± 0.1	>12.5
KKKChaG	>25	>25	>25	>25
etoposide	3.1 ± 0.1	>100	>100	>25
cisplatin	5.8 ± 1.4	15 ± 3.4	>100	>12.5
celastrol	0.9 ± 0.1	22 ± 6.0	31 ± 2.2	>25

a EC_50_ value after incubation with **4** for 5 h.

### Effect of Inhibitors of Intracellular Events on Cell Death Induced by TPH-ACs

In our previous papers, we reported that the cell death induced by **1**–**3** is considerably inhibited by carbonyl cyanide 3-chlorophenylhydrazone (CCCP), which is an uncoupling reagent and an inhibitor of mitochondrial Ca^2+^ uptake,^34−37^ while cell death was negligibly inhibited by benzyloxycarbonyl-VAD(OMe)-fluoromethylketone (Z-VAD-fmk, a broad caspase inhibitor),^38−40^ necrostatin-1 (a RIPK-1 inhibitor and necroptosis inhibitor),^41−43^ and 3-methyladenine (3-MA), an inhibitor of autophagosome formation that functions by inhibiting the action of type III phosphatidylinositol 3-kinases (PI3K)^44−46^ (the structures of these inhibitors are shown in Chart S2). This finding allowed us to conclude that the TPH-AC-induced cell death is paraptosis rather than apoptosis, necroptosis, or autophagy.^26^

**Figure 4:**
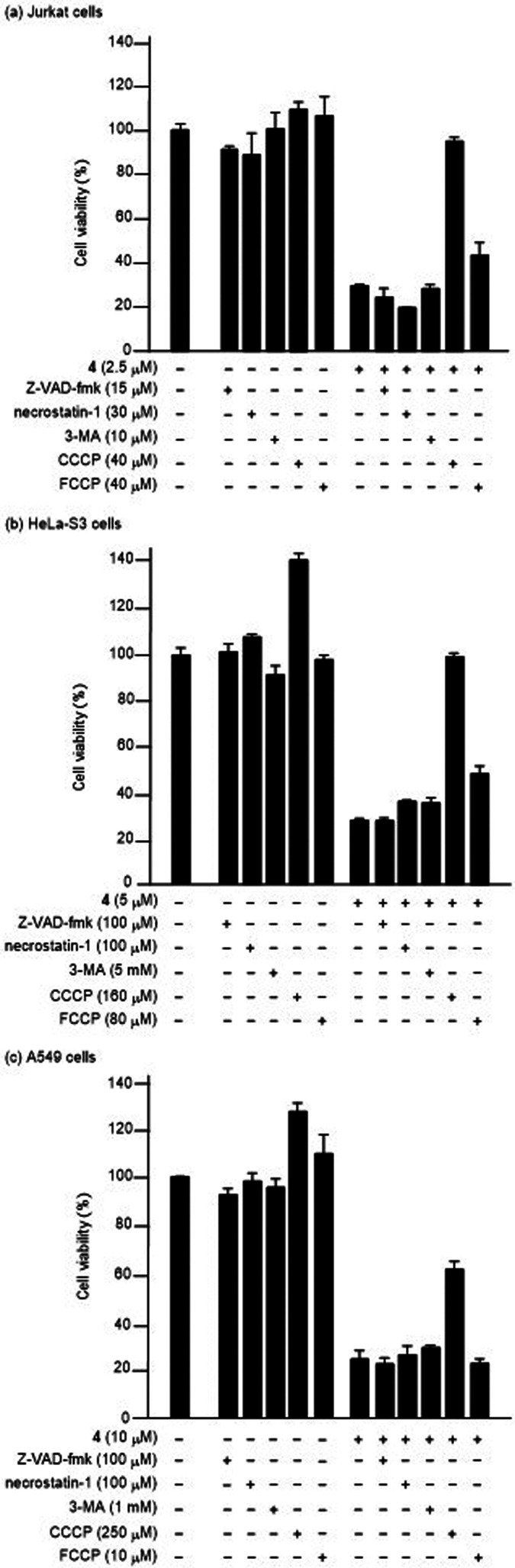
Results of an MTT assay of Jurkat (a), HeLa-S3 (b), and A549 cells (c) after the treatment with **4** in the presence of Z-VAD-fmk, necrostatin-1, 3-MA, CCCP, FCCP and then **4** at the indicated concentrations of these agents.

In the present work, we conducted MTT assays of HeLa-S3 and A549 cells treated with **4** and celastrol in the presence of Z-VAD-fmk, necrostatin-1, 3-MA, CCCP and trifluoromethoxy carbonyl cyanide phenylhydrazone (FCCP), which is an uncoupler of oxidative phosphorylation in mitochondria.^47^ Jurkat, HeLa-S3, and A549 cells were incubated with these inhibitors and then treated with **4** for 1 h and celastrol for 24 h at 37 °C under 5% CO_2_. As shown in Figure 4, the **4**-induced cell death in Jurkat, HeLa-S3 and A549 cells was considerably inhibited by CCCP, but negligibly inhibited by Z-VAD-fmk, necrostatin-1, and 3-MA, which strongly suggests that apoptosis, necroptosis, and autophagy are unlikely in these cancer cell lines. In addition, celastrol-induced cell death was negligibly inhibited by any inhibitors, as shown in Figure S6.

### Changes in Calcium Concentrations in Mitochondria and Cytosol

**Figure 5:**
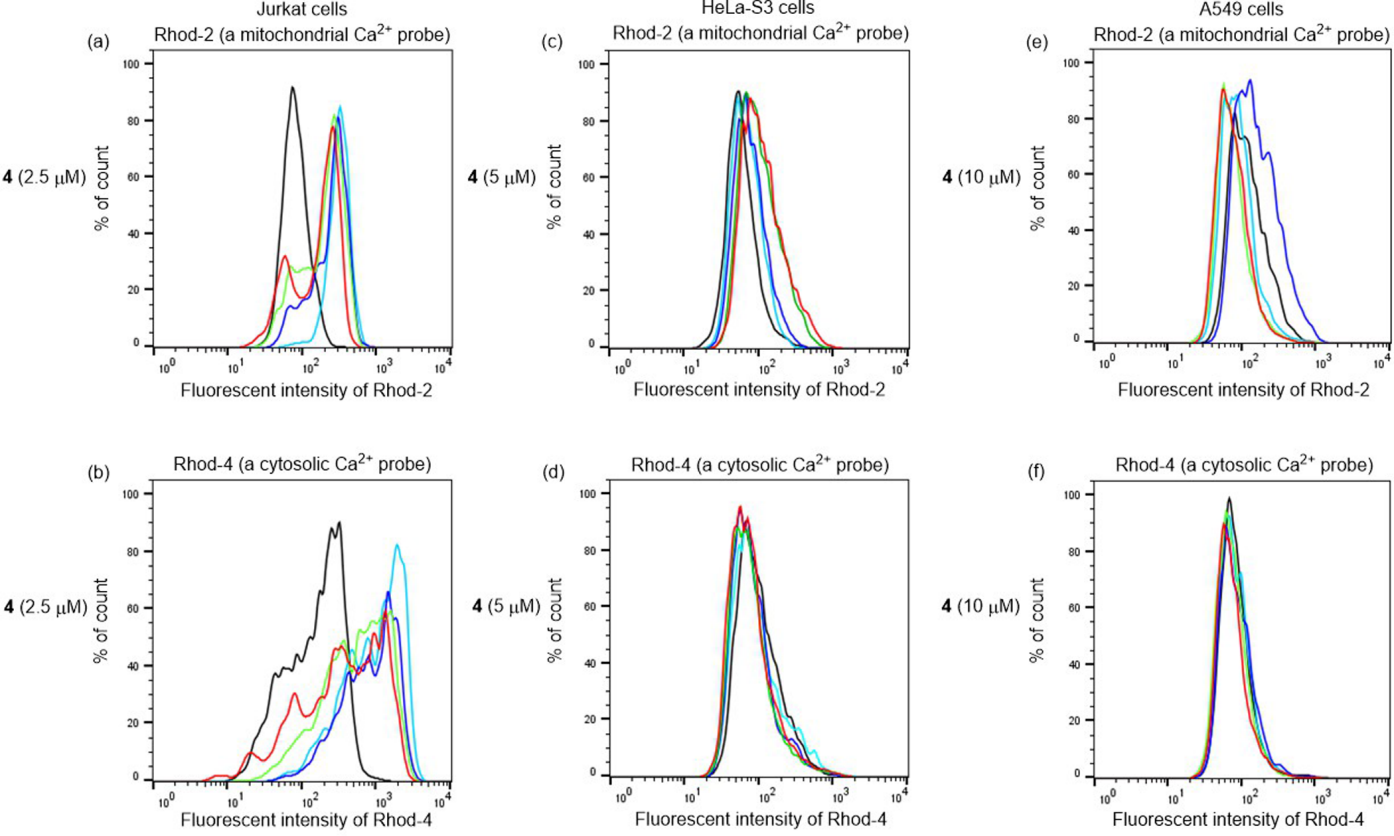
Flow cytometry analysis of Jurkat (a, b), HeLa-S3 (c, d), and A549 cells (e, f) after treatment with Rhod-2 (5 μM) and **4** (2.5 μM for Jurkat, 5 μM for HeLa-S3 and 10 μM for A549 cells) (a, c, e), and Rhod-4 (5 μM) and **4** (b, d, f). Different colors mean different incubation time with **4**: control (black), 15 min (light blue), 30 min (blue), 45 min (green), and 60 min (red).

Since it was reported that the transfer of Ca^2+^ from the ER to mitochondria is crucial in paraptosis II in Jurkat cells induced by **3**,^26^ we conducted the measurement of Ca^2+^ concentrations in mitochondria and cytosol in Jurkat, HeLa-S3, and A549 cells after treatment with **4** by flow cytometric analysis. Jurkat, HeLa-S3, and A549 cells were preincubated with Rhod-2/AM (5 μM), a probe of mitochondrial Ca^2+^,^48^ or Rhod-4/AM (5 μM), an indicator of cytosolic Ca^2+^,^49^ for 30 min and then treated with **4** (2.5, 5, or 10 μM) for various incubation times (0, 15, 30, 45, 60 min). Figure 5 shows the emission intensity of Rhod-2/AM and Rhod-4/AM in Jurkat cells and that of Rhod-2/AM in HeLa-S3 and A549 cells after treatment with **4** for 0, 15, 30, 45, and 60 min, which suggests that **4** promotes an increase in mitochondrial Ca^2+^ concentrations.

In addition, we examined the time-dependent change of fluorescence emission from Rhod-2/AM and Rhod-4/AM on fluorescence microscopy and microplate reader after treatment with **4**. The considerable enhancement of red emissions from Rhod-2/AM was observed in Jurkat cells after incubation with **4** in about 30 min, as shown in Figures S7 and S8a, which is parallel to the observation shown in Figure 5a. In Figure 5b as well as Figure S8a, moderate∼weak enhancement was observed in the fluorescence intensity of Rhod-4/AM.

We also examined the effect of **4** on the emission of Rhod-2/AM and Rhod-4/AM in HeLa-S3 and A549 cells, as shown in Figures S7, S8b and S8c. The enhancement of the red emission from Rhod-2/AM was observed in HeLa-S3 and A549 cells after incubation with **4** for 1 h, while there was a negligible enhancement of emission from Rhod-4/AM. These results are almost parallel to the observation in Figures 5c–f, confirming the generality of the Ca^2+^ overload in mitochondria rather than in the cytosol in these cancer cells. Figure S8d shows a somewhat slower emission enhancement from Rhod-2/AM in IMR90 cells and this point is described below.

It has been reported that celastrol promotes an increase in Ca^2+^ concentration in the cytosol.^12,13,22^ Therefore, we measured the emission changes of Rhod-2/AM and Rhod-4/AM for about 4 h after addition of celastrol, and observed a moderate enhancement of Rhod-2/AM rather than Rhod-4/AM (Figure S8e).

For comparison, we examined the time-dependent fluorescence changes of Rhod-2/AM and Rhod-4/AM in HeLa-S3 and A549 cells after addition of celastrol (a paraptosis I inducer) and cisplatin (an apoptosis inducer) on fluorescence microscopy, in which a mitochondrial and cytoplasmic Ca^2+^ overload in HeLa-S3 cells and a mitochondrial Ca^2+^ overload in A549 cells proceeded in 2–6 h, while the cytoplasmic Ca^2+^ concentration was negligibly changed by celastrol (Figures S9 and S10). On the other hand, a weak enhancement of the red emissions from Rhod-2/AM was observed in HeLa-S3 cells after incubation with cisplatin, as shown in Figure S11, which indicated a small increase in mitochondrial Ca^2+^.

### Induction of Membrane Fusion Between Mitochondria and the ER in Jurkat, HeLa-S3, and A549 Cells by 4

**Figure 6:**
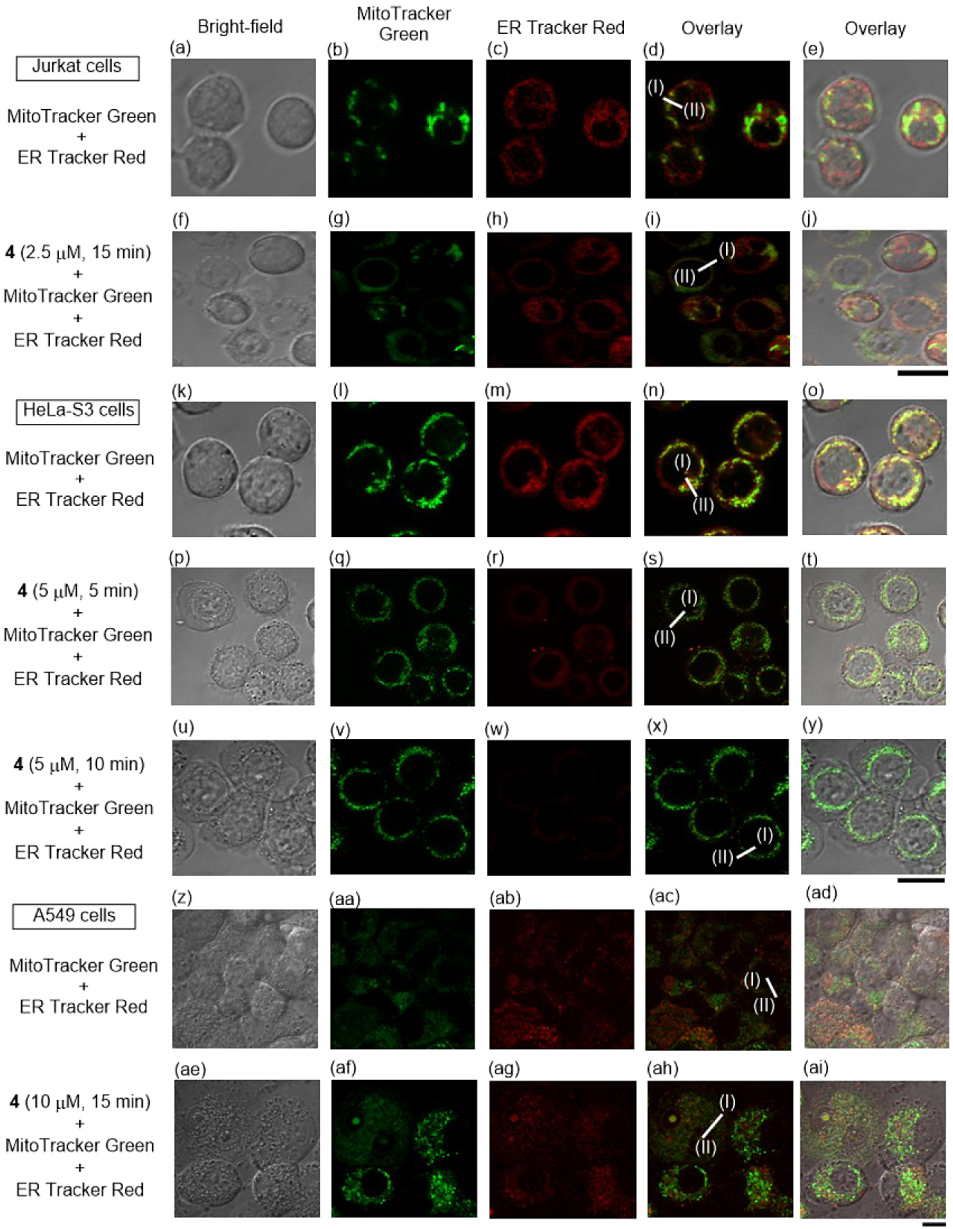
Typical fluorescence confocal microscopy images of Jurkat, HeLa-S3, and A549 cells treated with MitoTracker Green, and ERTracker Red in the presence of **4** (2.5, 5, or 10 μM). (a) and (f) Bright-field images of Jurkat cells, (b) and (g) emission images of MitoTracker Green, (c) and (h) emission images of ERTracker Red, (d) overlay images (a–c), (e) overlay images (a–d), (i) overlay images (f–h), and (j) overlay images (f–i). (k), (p) and (u) Bright-field images of HeLa-S3 cells, (l), (q) and (v) emission images of MitoTracker Green, (m), (r) and (w) emission images of ERTracker Red, (n) overlay images (k–m), (o) overlay images (k–n), (s) overlay images (p–r), (t) overlay images (p–s), (x) overlay images (u–w), and (y) overlay images (u–x). (z) and (ae) Bright-field images of A549 cells, (aa) and (af) emission images of MitoTracker Green, (ab) and (ag) emission images of ERTracker Red, (ac) overlay images (z–ab), (ad) overlay images (z–ac), (ah) overlay images (ae-ag), and (ai) overlay images (ae-ah). Excitation at 473 nm for (b), (g), (l), (q), (v), (aa) and (af) and at 559 nm for (c), (h), (m), (r), (w), (ab) and (ag). Exposure time was 20 μs/pixel. Scale bar (black) is 10 μm.

Next, we conducted costaining experiments of the ER and mitochondria in Jurkat, HeLa-S3, and A549 cells with ERTracker Red, and MitoTracker Green, which are selective probes of the ER and mitochondria, respectively. Jurkat, HeLa-S3, and A549 cells were treated with MitoTracker Green and ERTracker Red for 1 h, respectively, and then incubated with **4** for 15 min (Jurkat and A549), or for 5 and 10 min (HeLa-S3). As shown in Figure 6a–e (Jurkat), 6k-o (HeLa-S3), and 6z-ad (A549), a weak overlap of the emission from MitoTracker Green and ERTracker Red was observed before the addition of **4,** and a considerable overlap was observed after incubation with **4** as shown in Figure 6f–j (Jurkat), 6p-y (HeLa-S3), and 6ae-ai (A549). In these experiments, faster degradation of the ER was observed in HeLa-S3 than in Jurkat and A549 cells.^50^

**Figure 7:**
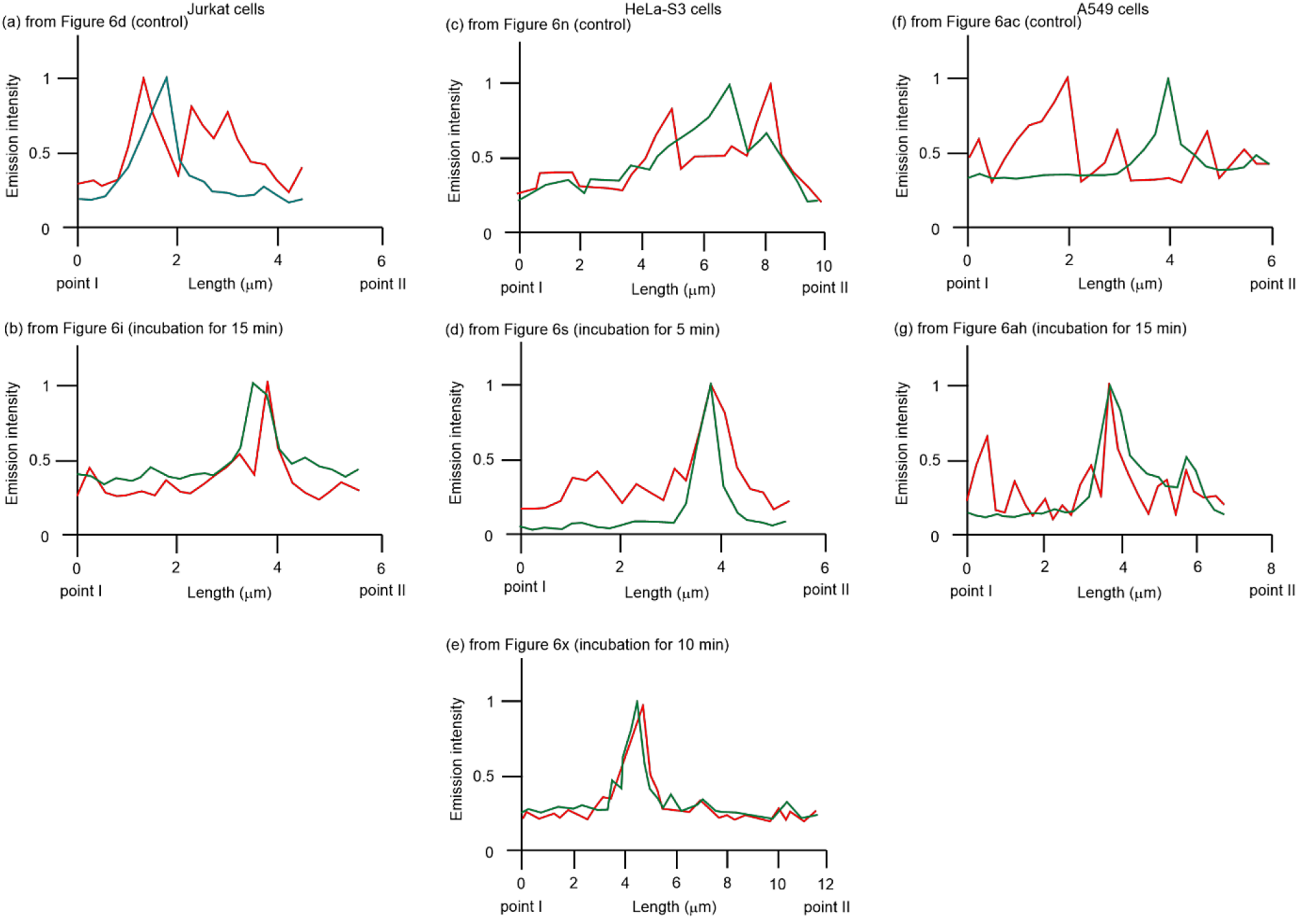
Emission intensity profiles of MitoTracker Green (green curves) and ERTracker Red (red curves) in Jurkat (a, b), HeLa-S3 (c–e), and A549 cells (f, g) before and after treatment with **4** (2.5, 5, or 10 μM) from point I to point II in Figure 6d (a), Figure 6i (b), Figure 6n (c), Figure 6s (d), Figure 6x (e), Figure 6ac (f) and Figure 6ah (g), respectively.

The emission intensity profiles of MitoTracker Green and ERTracker Red are indicated by the green and red curves, respectively, in Figure 7 between points I and II in the corresponding images of Figure 6 (more intensity profiles at other points are presented in Figure S12). Figure 7a (Jurkat), Figure 7c (HeLa-S3), and Figure 7f (A549) show a partial overlap of the emission from MitoTracker Green and ERTracker Red before treatment with **4** and Figures 7b (Jurkat), Figures 7d–e (HeLa-S3), and Figure 7g (A549) show their considerable overlap, indicating that **4** induces tethering or membrane fusion between the ER and mitochondria in all of these cells.

**Figure 8:**
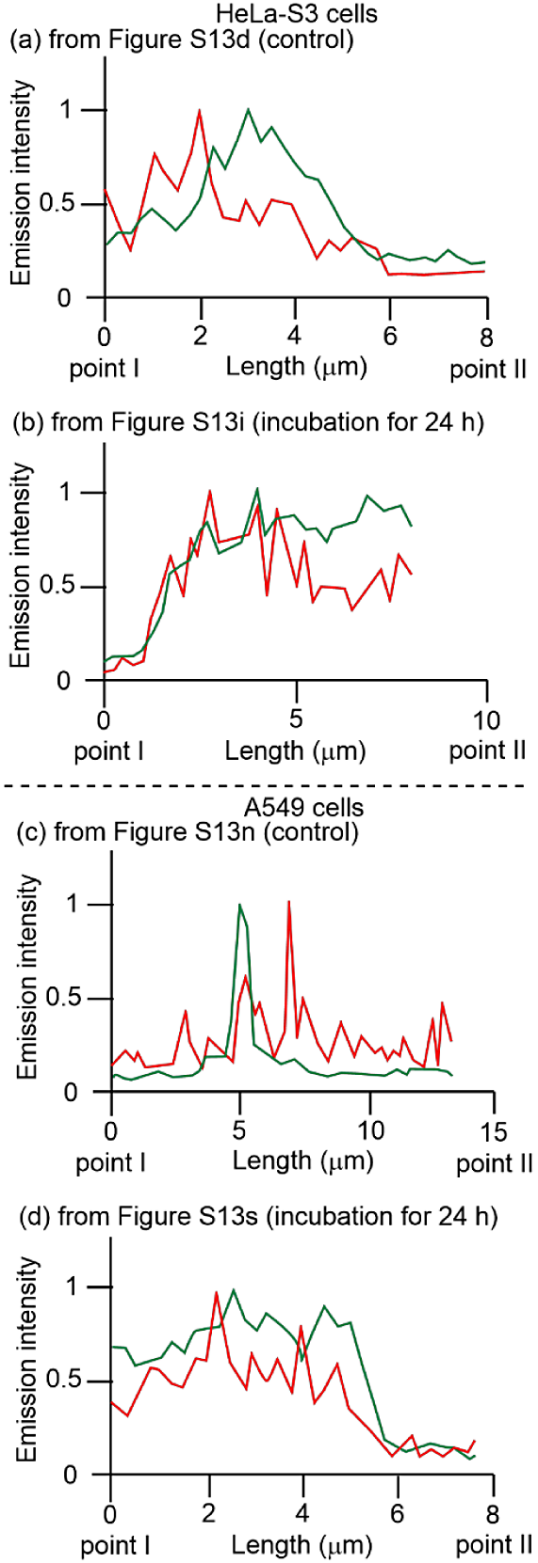
Emission intensity profiles of MitoTracker Green (green curves) and ERTracker Red (red curves) in HeLa-S3 (a, b) and A549 cells (c, d) before and after treatment with celastrol (100 μM) from point I to point II in Figure S13d (a), Figure S13i (b), Figure S13n (c), and Figure S13s (d).

For comparison, we also examined the effect of celastrol on the interaction between the ER and mitochondria in HeLa-S3 and A549 cells. As shown in Figure S13a–e along with Figure 8a (HeLa-S3) (also in Figure S13k–o along with Figure 8c (A549)), a weak overlap of the emission from MitoTracker Green and ERTracker Red was observed before the addition of celastrol. A weaker overlap was observed after incubation with celastrol than after incubation with **4**, as shown in Figure S13f–j along with Figure 8b (HeLa-S3) (also in Figure S13p–t along with Figure 8d (A549 cells). More intensity profiles at other points are shown in Figure S14.

### Effect of Inhibitors of Intracellular Ca^2+^-related Events Between the ER and Mitochondria

**Scheme 4:**
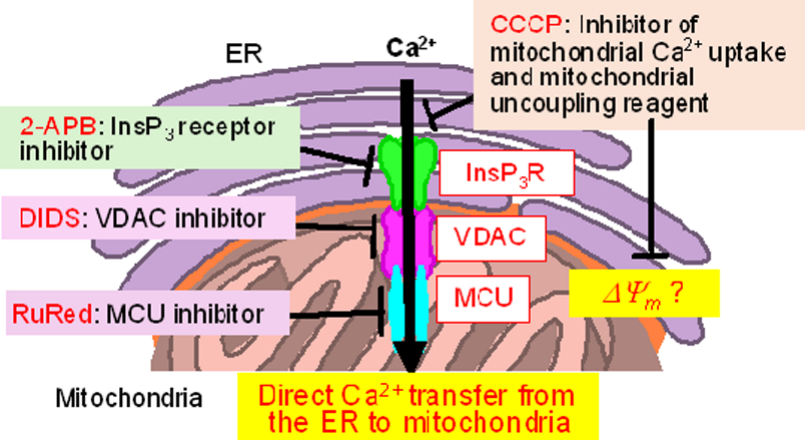
Relationship Between InsP_3_ Receptor, VDAC, MCU and Mitochondrial Ca^2+^ uptake and Their Inhibitors in the Proposed Processes of Paraptosis II

The aforementioned results suggest that mitochondrial Ca^2+^ uptake is promoted by **4** in the paraptotic II processes. Scheme 4 shows the proposed relationship of InsP_3_ (inositol 1,4,5-trisphosphate) receptor (InsP_3_R), the voltage-dependent anion channel (VDAC), and mitochondrial Ca^2+^ uniporter (MCU) involved in Ca^2+^ transport from the ER and mitochondria at their interface.^51−53^ We previously reported that Ruthenium Red (RuRed), (an inhibitor of MCU),^54^ and 2-aminophenyl borate (2-APB), an antagonist of InsP_3_R, (the structures of these inhibitors are shown in Chart S3),^55^ inhibit paraptosis II induced by TPH-ACs (Scheme 4) to some extent.^26^

**Figure 9:**
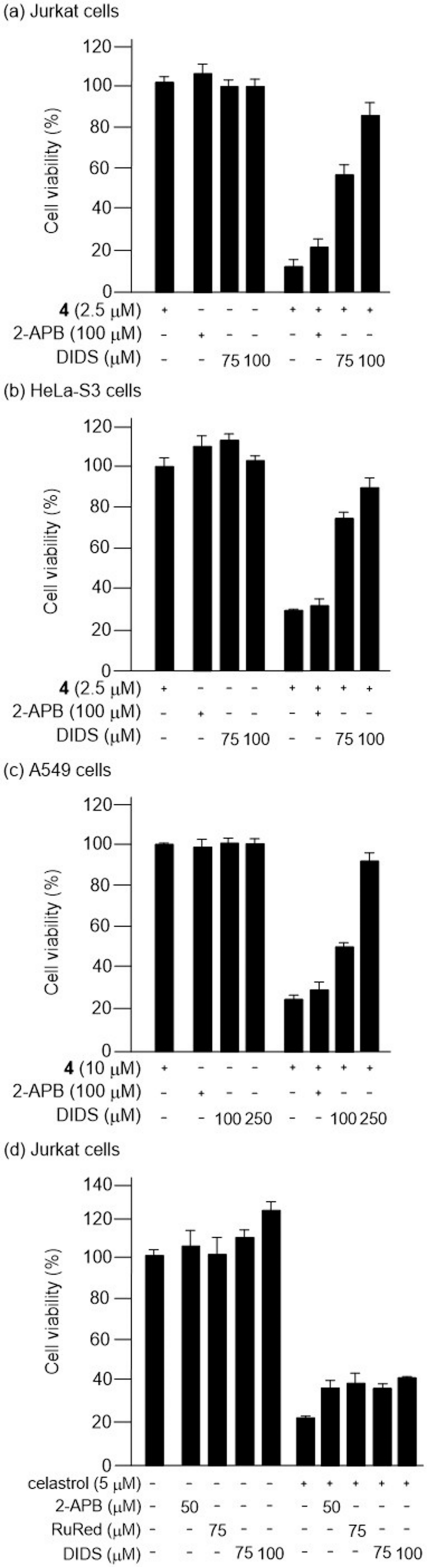
(a–c) The results of MTT assays of Jurkat (a), HeLa-S3 (b), and A549 cells (c) pretreated with 2-APB and DIDS and then treated with **4** at the indicated concentrations. (d) The results of MTT assays of Jurkat cells pretreated with 2-APB, RuRed, and DIDS prior to addition of celastrol.

Then, we decided to check the role of the voltage-dependent anion channel (VDAC), which is expressed on the outer mitochondrial membrane and is known to function as transporters of metabolites such as ATP, ADP, and Ca^2+^.^56−64^ For this purpose, the effect of an inhibitor of VDAC, 4,4′-diisothiocyano-2,2′-stilbenedisulfonic acid (DIDS)^65−69^ (Chart S3) on **4**-induced paraptosis II was checked. Jurkat, HeLa-S3, and A549 cells were preincubated with DIDS (75, 100, or 250 μM for these cancer cell lines, respectively) for 3 h and then treated with **4** for 1 h. The results of MTT assays indicate that DIDS considerably inhibits paraptosis II induced by **4** in Jurkat (Figure 9a), HeLa-S3 (Figure 9b), and A549 cells (Figure 9c). The inhibitory effect of 2-APB and RuRed on the **4**-induced paraptosis II was found to be rather weak in this work. It should also be mentioned that it was reported that thapsigargin, which is an inhibitor of Ca^2+^ transport between the sarco-endoplasmic reticulum (SR/ER) negligibly inhibit paraptosis II induced by **3**, one of our previous TPH-ACs.^26^

We compared the effects of 2-APB, RuRed, and DIDS on paraptosis I induced by celastrol with paraptosis II induced by **4**. Jurkat cells were preincubated with 2-APB (50 μM), RuRed (75 μM), and DIDS (75 or 100 μM) and then treated with celastrol (5 μM) for 24 h. As shown in Figure 9d, these inhibitors did not significantly stop cell death induced by celastrol.

We checked the direct interaction of **4** and celastrol with DIDS and found the formation of weak precipitates in a mixture of **4** and DIDS in water (containing ca. 1% DMSO) at neutral pH, possibly due to the electrostatic interaction between polycationic **4** (possibly 12+) and dianionic DIDS (DIDS^2–^).^70^ Therefore, we could not exclude the possibility of the suppression of paraptosis II by the complexation of TPH-ACs with DIDS inside cancer cells and hence DIDS was not used for further mechanistic studies described below. It is considered that interaction between anionic celastrol (due to the anionic form of carboxylate) with DIDS^2–^ is very weak.

### Observation of Fe^2+^ and Zn^2+^ Ions in Paraptosis Induced by 4

Apoptosis inducers are known to cause a concentration change in Fe^2+^ and Zn^2+^ ions in Jurkat cells during apoptotic processes.^71^ Therefore, we examined the effect that Zn^2+^ ions exert on the cytotoxicity of **4** against Jurkat cells. A mixture of **4** and Zn(NO_3_)_2_ was incubated for 1 h at 37 °C. The mixture was then added to Jurkat cells and incubated for 1 h at [**4**] = 0.16–10 μM and [Zn^2+^] = 15 μM. MTT assays then were performed. As shown in Figure S15, the EC_50_ value of **4**+Zn(NO_3_)_2_ (1.8 μM) was nearly the same as that of **4** alone (1.5 μM), which suggests that Zn^2+^ had a negligible effect on cytotoxicity.

Furthermore, we conducted fluorescent microscopic analysis to check the change in the concentrations of these ions during cell death induced by **4** by using Mito-FerroGreen (a probe for labile Fe^2+^ in mitochondria)^72,73^ and zinquin (a probe for intracellular Zn^2+^)^74−79^ (the structures of these probes are shown in Chart S4). The enhancement of the green fluorescence emission from Mito-FerroGreen shown in Figure S16 and blue emission from zinquin shown in Figure S17 suggests that increases in both free-Fe^2+^ and free-Zn^2+^ during apoptosis were induced by cisplatin and etoposide.^50,80^ On the other hand, the intensity of these emissions was negligibly enhanced in Jurkat cells treated with **4** and celastrol. These facts imply that the change in intracellular free-Zn^2+^ and free-Fe^2+^ was negligible during both paraptosis I (by celastrol) and paraptosis II (by TPH-ACs) and that the destruction of Ca^2+^ homeostasis is important in these paraptosis processes.

### Effect of ATP and ADP on Paraptosis Induced by 4

An increase in ATP concentrations in the mitochondria is known to enhance the transport of Ca^2+^ from the extracellular space into mitochondria.^81 −86^ Therefore, we examined the effect of ATP uptake into cancer cells by agonists of P2X, which are ATP ligand-gated ion channel receptors. Because ADP and ATP had been established as a natural agonist and a competitive antagonist of P2Y1 which are G protein-coupled receptors, respectively,^81−86^ we conducted MTT assays of Jurkat cells with **4** and celastrol, paraptosis inducers, and etoposide, an apoptosis inducer, in the presence of ATP and ADP. As shown in Figure S18, the EC_50_ values of compound+ATP and compound+ADP approximated those of the compounds alone. The data suggests that ATP and ADP (extracellular) exert negligible effects on the cytotoxicity of these cell-death inducers.

### Mechanistic Study Regarding Cancer Cell/Normal Cell Selectivity of TPH-ACs

**Figure 10:**
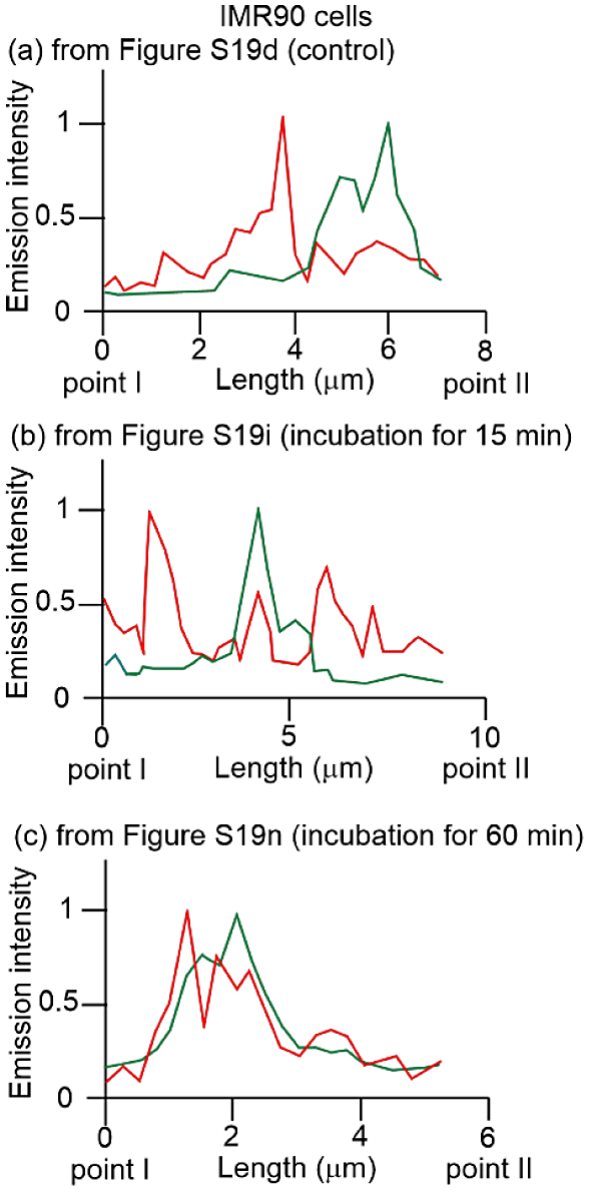
Emission intensity profiles of MitoTracker Green (green curves) and ERTracker Red (red curves) in IMR90 cells before and after treatment with **4** (5 or 50 μM) from point I to point II in Figure S19d (a), Figure S19i (b), and Figure S19n (c), respectively.

TPH-ACs exhibit lower levels of toxicity in IMR90 cells than that in cancer cell lines, as described above (Table 1). Next, we then conducted costaining experiments of the ER and mitochondria in IMR90 cells with ERTracker Red and MitoTracker Green. IMR90 cells were treated with MitoTracker Green and ERTracker Red for 1 h, respectively, and were then incubated with **4** for either 15 or 60 min. As shown in Figure S19 and in Figure 10, a weak overlap of the emission from MitoTracker Green and ERTracker Red was observed both before and after the addition of **4** (more points for the intensity profile are presented in Figure S20). One more possibility suggests the occurrence of smaller numbers of VDAC and MCU in normal cells^87,88^ or slower response of these Ca^2+^ channels, as shown in Figure S8e, than that in cancer cells.

In addition, we examined the time-dependent fluorescence changes in either Rhod-2/AM or Rhod-4/AM in IMR90 cells using fluorescence microscopy and a microplate reader after treatment with **4** for 60 min. As shown in Figure S8d, enhancement of the red emissions from Rhod-2/AM in IMR90 cells is induced more slowly than in other cancer cell lines (see also Figure S21). We assume that the differences between cancer cells and normal cells account for the cancer cell selectivity of TPH-ACs.

### Possible Mechanisms of Paraptosis II Induced by TPH-ACs in Jurkat, HeLa-S3, and A549 Cells

The aforementioned mechanistic studies are summarized as follows.

**Figure 11:**
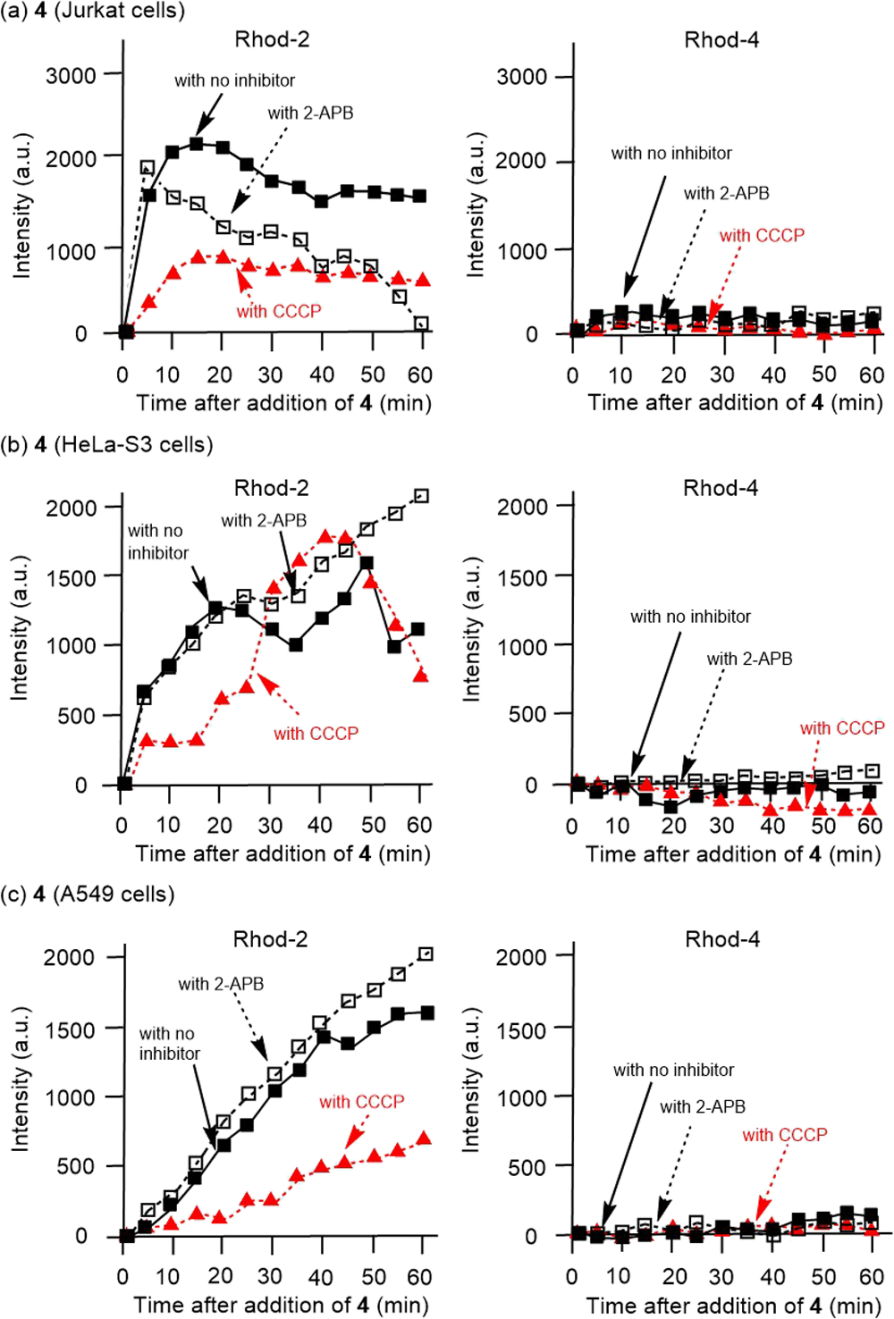
Time-dependent change of fluorescence emission from Rhod-2/AM (left side) and Rhod-4/AM (right side) (excitation at 540 nm and emission at 590 nm) in Jurkat (a), HeLa-S3 (b), and A549 cells (c) measured on microplate reader after the pretreatment with CCCP (40 μM for Jurkat, 160 μM for HeLa-S3 and A549 cells) and 2-APB (100 μM for Jurkat, HeLa-S3 and A549 cells) prior to the addition of **4** (2.5 μM for Jurkat, 5 μM for HeLa-S3, and 10 μM for A549 cells). Black plain curves with filled squares indicate emission from Rhod-2/AM and Rhod-4/AM in the absence of CCCP and 2-APB. Black dashed curves with open squares indicate emission from Rhod-2/AM and Rhod-4/AM in the presence of 2-APB. Red dashed curves with filled triangles indicate emission from Rhod-2/AM and Rhod-4/AM, respectively, in the presence of CCCP at the concentrations described above. A.u. is arbitrary unit.

**Scheme 5:**
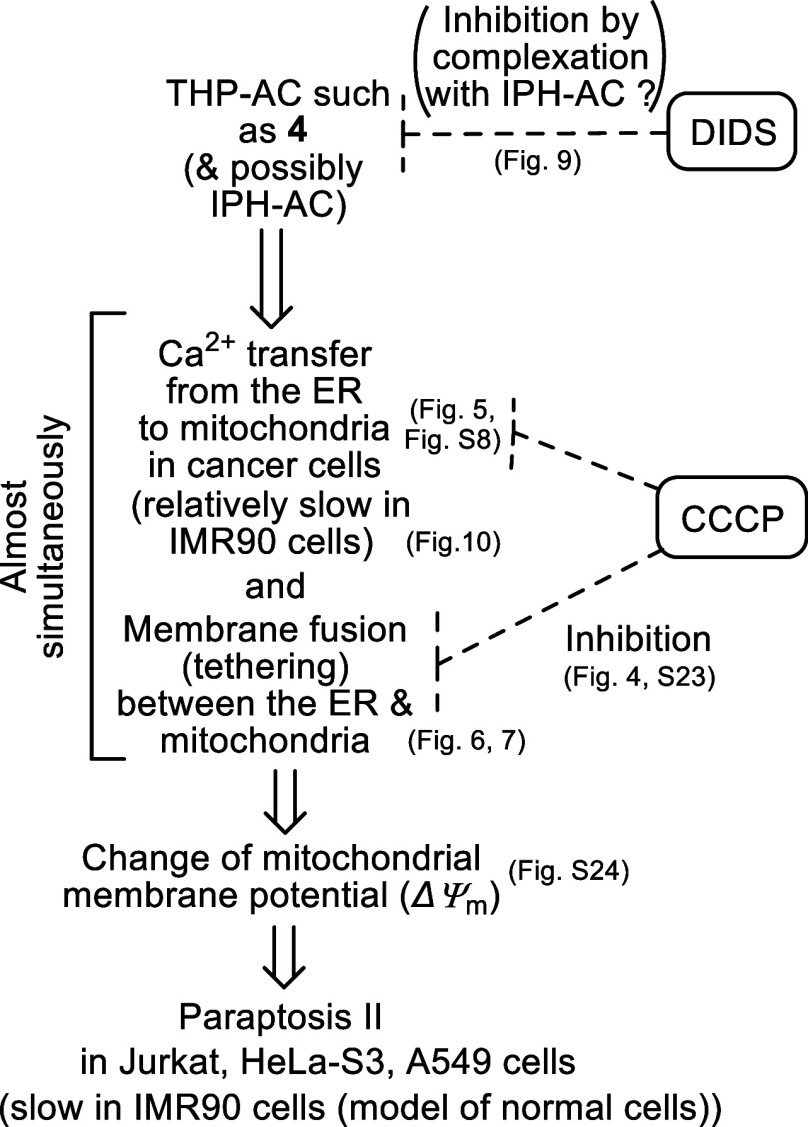
Revision of Proposed Mechanism and Time-course of Paraptosis II Induced by TPH-ACs Such as **4** and Relationship with Paraptosis II Inhibitors in Jurkat, HeLa-S3, A549 and IMR90 Cells

(1) TPH-ACs **4**–**7** (Scheme 1) were newly designed and synthesized in this work. The stability of **5** with cyclic peptide units is higher than that of **4** with corresponding linear peptide units (Figure S1). We found that **4** exhibits a higher level of cytotoxicity compared with that of **3** against these cancer cell lines, which was possibly due to the effect of a somewhat hydrophobic Cha residue in the peptide units of **3**.

(2) TPH-ACs **4**–**7** have potent cytotoxicity against Jurkat, HeLa-S3, and A549 cells, as examined by PI-staining experiments and MTT assays. These TPH-ACs induce cell death after treatment for 1 h with morphological changes similar to that induced by our previous TPH-ACs such as **3** (Figures S2–4), albeit with EC_50_ values that were smaller than those of **3** (Figure 3a–c and Table 1). Moreover, the cytotoxicity of TPH-ACs against IMR90 cells, a model of a normal cell line, is weaker than that against the cancer cells (Figure 3d and Table 1).

(3) TPH-ACs such as **4** induce membrane fusion (or tethering) between the ER and mitochondria, as indicated by costaining experiments of the ER and mitochondria in HeLa-S3 and A549 cells as well as in Jurkat cells, which is suggested to be common phenomena in paraptosis II processes (Figures 6 and 7). On the other hand, a weaker overlap was observed in cancer cell lines after incubation with celastrol than after incubation with **4** (Figures S13 and 8 in the text). It was reported by D’Eletto, M. et al. that the ER and mitochondria undergo membrane fusion by stimulating with transglutaminase 2 (TG2).^89^ However, the effect of a TG2 inhibitor, dansylcadaverine, on paraptosis II was negligible in our previous work.^26^

(4) The **4**-induced paraptosis II in Jurkat, HeLa-S3 and A549 cells is considerably inhibited by CCCP (an inhibitor of mitochondrial Ca^2+^ uptake)^36^ and DIDS (an inhibitor of VDAC), but negligibly inhibited by inhibitors of apoptosis, necroptosis, and autophagy (Figures 4 and 9). On the other hand, the cell death induced by celastrol, which is known to induce a previously reported type of paraptosis (paraptosis I), was negligibly inhibited by these agents (Figure S6).

(5) TPH-AC **4** considerably promotes an increase in Ca^2+^ concentrations in mitochondria as observed using flow cytometry, fluorescence microscopy, and a microplate reader (Figures 5, S7, and S8). Observation of Ca^2+^ overload in mitochondria of Jurkat, HeLa-S3, and A549 cells by flow cytometry (Figure 5) and a microplate reader (Figure S8) strongly support the enhancement of Ca^2+^ concentrations in mitochondrial rather than in cytoplasm.

(6) To analyze the inhibitory effect of CCCP on paraptosis II induced by **4** in Jurkat, HeLa-S3, and A549 cells, the change of Ca^2+^ concentrations in mitochondria (by Rhod-2/AM) and in cytosol (by Rhod-4/AM) of these cancer cells was measured on flow cytometry after the pretreatment with CCCP prior to the addition of **4** (Figure S22). When Jurkat cells were pretreated with CCCP (see Figures S22a, b, g, h), the emission from Rhod-2/AM was enhanced by CCCP in the initial 15 min and then suppressed later (please compare Figure 5a in the text with Figure S22b), although broad signals were observed with respect to the Ca^2+^ concentrations in mitochondria and cytosol regardless of the presence or absence of **4** (see Figures S22a, b, g, h). In HeLa-S3, and A549 cells, the emission from Rhod-2/AM was suppressed by CCCP (Figure 5c in the text vs Figure S22d and Figures 5e vs S22f). The effect of DIDS on paraptosis II is not conclusive, because of the finding of direct salt formation of **4** with DIDS in aqueous solution at neutral pH, as described above. Therefore, DIDS was not used for further mechanistic studies.

(7) It should be mentioned that the results of flow cytometry of Jurkat cells, which were treated with **4** in the presence of CCCP, shown in Figure S22 (especially, Figures S22g and S22h) show broad and complicated spectra, possibly due to the detection of different cells which include Ca^2+^ at different concentrations. Then, we have decided to use a microplate reader, which was expected to detect the total emission from Rhod-2/AM (mitochondrial Ca^2+^) and Rhod-4/AM (cytosol Ca^2+^) in Jutkat, HeLa-S3 and A549 cells after addition of **4** in the presence of CCCP and 2-APB. As summarized in Figure 11, emission enhancement of Rhod-2/AM after the addition of **4** was moderately inhibited by CCCP (red dashed curves), while the effect of 2-APB (black dashed curves) was not so strong. On the other hand, the effect of CCCP and 2-APB on the emission of Rhod-4/AM was negligible, because the change of emission from Rhod-4/AM was negligible even after the addition of **4** (black curves with black filled squares). Although complete inhibition of Ca^2+^ transfer from the ER to mitochondria by CCCP was not observed, these results may support the important roles of mitochondrial Ca^2+^ uptake in paraptosis II induced by **4**.

(8) The costaining experiments of the ER and mitochondria in Jurkat cells after treatment with **4** in the presence of CCCP were also conducted. As summarized in Figure S23, the overlap of the emission from MitoTracker Green and ERTracker Red by **4** (2.5 μM) was considerably inhibited by CCCP (40 μM).^90^ Consideration on aforementioned results together with Figure 11 in the text (weak inhibitory activity of CCCP against mitochondrial Ca^2+^ overload) and Figure S23 strongly suggests that CCCP exhibits inhibitory effect on the-ER-mitochondria fusion (or tethering) and the Ca^2+^ transfer from the ER to mitochondria.

(9) We measured the mitochondrial membrane potential (MMP, *ΔΨ*_m_) in Jurkat cells using 1,1′,3,3,3′,3′-hexamethylindodicarbocyanine iodide (DilC1(5)),^91^ which is a probe that responds to *ΔΨ*_m_. Jurkat cells were stained with DilC1(5) (500 nM) for 30 min and then treated with **4** (2.5 μM) for 10–60 min for the observation on fluorescence microscopy. As shown in Figure S24, the emission of DilC1(5) starts to decrease in ca. 20–40 min after addition of **4**, while weakly CCCP inhibits the decrease in the emission of DilC1(5) by **4**.^92^ Although more detailed experiments will be required in the next work, these results suggest a possibility that the *ΔΨ*_m_ values decrease in later steps of paraptosis II than Ca^2+^ transfer to mitochondria and membrane fusion between the ER and mitochondria.

(10) Scheme 5 summarizes our revised proposal to explain the mechanism of paraptosis II induced by **4** in Jurkat, HeLa-S3, and A549 cells and the reaction points of two paraptosis II inhibitors, DIDS and CCCP. As described above, **4** induces Ca^2+^ overload from the ER to mitochondria in these cells and induce membrane fusion between the ER and mitochondria almost simultaneously in 10–20 min after addition of **4**. After that, the *ΔΨ*_m_ values are decreased. It is suggested that CCCP exhibits inhibitory effect on the Ca^2+^ transfer from the ER to mitochondria and the ER-mitochondria fusion (or tethering) and this point is yet to be studied. Inhibition of paraptosis II by DIDS could be explained by its complexation with **4** (and IPH-ACs such as **1a**) due to the electrostatic interaction between them. Most importantly, it was concluded that TPH-ACs such as **4** induce paraptosis II via similar mechanism at least in these three cancer cell lines.

(11) The cytotoxicity of TPH-ACs against IMR90 cells is weaker and slower (EC_50_ value of **4** is lowered to 4.4 μM after incubation for 5 h) than that against the aforementioned three cancer cell lines (Figure 3d, Table 1, and Scheme 5). This result is attributed to slower mitochondrial Ca^2+^ increase in IMR90 cells than that in cancer cells (Figure S8d) and weaker interaction between the ER and mitochondria than that in cancer cell lines even after treatment with **4**, as proven by the costaining experiments using MitoTracker Green and ERTracker Red (Figures S19 and 10).

(12)Although intracellular free-Fe^2+^ and free-Zn^2+^ ions during apoptosis in Jurkat cells were increased, the effect of intracellular Fe^2+^ and Zn^2+^ ions during paraptosis induced by **4** and celastrol was negligible (Figures S16 and S17). The effects of ATP and ADP, which are an agonist and an antagonist of P2Y1 on paraptosis II, were also negligible (Figure S18).

## Conclusions

In this paper, we report the results of detailed mechanistic studies of the paraptosis that is induced by triptycene-peptide hybrids as amphiphilic conjugates (TPH-ACs) in Jurkat, HeLa-S3, and A549 cells. We confirmed that the paraptosis II induced by TPH-ACs is different from the cell death induced by celastrol, which induces paraptosis I in our definition. TPH-AC-induced cell death is inhibited by CCCP, 2-APB, and DIDS in these cancer cell lines, while celastrol-induced paraptosis I is not affected by these inhibitors. The results of experiments strongly suggest that TPH-ACs induce a transfer of Ca^2+^ into mitochondria possibly from the ER and membrane fusion of the ER and mitochondria in Jurkat, HeLa-S3, and A549 cells, resulting in the induction of paraptosis II in these three cancer cell lines, which is the main point in our hypothesis on paraptosis II. Although the details of mechanism of the inhibition of paraptosis II by CCCP is not fully understood, there is a high possibility that Ca^2+^ from the ER to mitochondria and the membrane fusion between the ER and mitochondria are inhibited by CCCP.

To the best of our knowledge, there is no approved anticancer drug that induces paraptosis II in cancer cells. As described in the Introduction, paraptosis (paraptosis I and II) are not fully understood and its deep understanding could lead to new strategies for the treatment of not only cancers but also autoimmune diseases and other related diseases. At the same time, we assume that the finding of potent inhibitors of intracellular events is very important for their mechanistic study. For example, Z-VAD-fmk is used as one of useful caspase inhibitors and contributes to characterization and mechanistic study of apoptosis. In this work, CCCP was found as a potent inhibitor of paraptosis II and the finding of more potent and selective inhibitors would contribute to the mechanistic studies of paraptosis.

A detailed mechanistic study concerning selective toxicity against cancer cell lines over normal cells (IMR90 cells in this study) suggests the time-dependent toxicity of TPH-ACs against cancer cells over normal cells, which could be one of the future strategies in cancer chemotherapy and in controlling toxicity of anticancer drugs.

We conclude that the results reported in this study provide useful information for not only the mechanistic studies of paraptosis, but also the development of drugs with the potential to target cancer cells while minimizing the side effects on normal cells.

## Supporting Information Available

The stability of **4** and **5** after treatment with trypsin (Figure S1), fluorescence microscopic images of Jurkat (Figure S2), HeLa-S3 (Figure S3), and A549 cells (Figure S4) after treatment with **3**, **4**, **5**, **6**, and **7**, fluorescence microscopic images of HeLa-S3, and A549 cells after treatment with celastrol (Figure S5), results of the MTT assays of HeLa-S3 and A549 cells after treatment with celasterol in the presence of Z-VAD-fmk, necrostatin-1, 3-MA, and CCCP (Figure S6), fluorescence microscopic images of Jurkat, HeLa-S3, and A549 cells treated with Rhod-2/AM or Rhod-4/AM in the presence of **4** (Figure S7), time-dependent change of fluorescence emission from Rhod-2/AM or Rhod-4/AM in Jurkat, HeLa-S3, A549, and IMR90 cells after addition of **4** and celastrol (Figure S8), fluorescence microscopic images of HeLa-S3 (Figure S9) and A549 cells (Figure S10) stained with Rhod-2/AM or Rhod-4/AM after the treatment with celastrol, fluorescence microscopic images of HeLa-S3 cells treated with Rhod-2/AM or Rhod-4/AM in the presence of cisplatin (Figure S11), emission intensity profiles of MitoTracker Green and ERTracker Red in Figure 6 (Figure S12), typical fluorescence confocal microscopy images of HeLa-S3 and A549 cells treated with MitoTracker Green, and ERTracker Red in the presence of celastrol (Figure S13), emission intensity profiles of MitoTracker Green and ERTracker Red in Figure S13 (Figure S14), the results of the MTT assay of Jurkat cells treated with **4** in the presence of Zn(NO_3_)_2_ (Figure S15), fluorescence microscopic images of Jurkat cells treated with Mito-FerroGreen in the presence of cisplatin, etoposide, **4** and celastrol (Figure S16), fluorescence microscopic images of Jurkat cells treated with zinquin in the presence of cisplatin, etoposide, **4**, and celastrol (Figure S17), results of the MTT assays of Jurkat cells after treatment with **4**, celastrol, and etoposide in the presence of ATP and ADP (Figure S18), typical fluorescence confocal microscopy images of IMR90 cells treated with MitoTracker Green, and ERTracker Red in the presence of **4** (Figure S19), emission intensity profiles of MitoTracker Green and ERTracker Red obtained from Figure S19 (Figure S20), fluorescence microscopic images of IMR90 cells treated with Rhod-2/AM or Rhod-4/AM in the presence of **4** (Figure S21), the results of flow cytometry analysis of Jurkat, HeLa-S3, and A549 cells after treatment with Rhod-2/AM and Rhod-4/AM in the presence of **4** and CCCP (Figure S22), typical fluorescent confocal microscopy images of Jurkat cells stained with MitoTracker Green, and ERTracker Red after the pretreatment with CCCP and **4** and the emission intensity profiles of MitoTracker Green and ERTracker Red (Figure S23), fluorescence microscopic images of Jurkat cells treated with DilC1(5) and **4** in the presence of CCCP (Figure S24), and the chemical structures of compounds, inhibitors and probes (Charts S1–S4) (PDF)
