# Supplementary material for: Induction of Paraptotic Cell Death in Cancer Cells by Triptycene–Peptide Hybrids and the Revised Mechanism of Paraptosis II
Source: Biochemistry. 2024 Aug 14;63(17):2111–30. doi: 10.1021/acs.biochem.4c00085 (PMC11375786; doi:10.1021/acs.biochem.4c00085)

Supporting Information

Induction of Paraptotic Cell Death in Cancer Cells by Triptycene-Peptide Hybrids and the Revised Mechanism of Paraptosis II

Mayuka Nii,[a] Kohei Yamaguchi,[a] Toshifumi Tojo,[a,b] Nozomi Narushima,[a] and Shin Aoki[a, b, c]*

[a]Faculty of Pharmaceutical Sciences, Tokyo University of Science, 2641 Yamazaki, Noda, 278-8510, Japan, [b]Research Institute for Science and Technology (RIST), Tokyo University of Science, 2641 Yamazaki, Noda, Chiba 278-8510, Japan, [c]Research Institute for Biomedical Sciences (RIBS), Tokyo University of Science, 2641 Yamazaki, Noda, Chiba 278-8510, Japan.

*Corresponding authors: E-mail, shinaoki@rs.tus.ac.jp

Address of home page: https://www.rs.noda.tus.ac.jp/aokilab/index.html

Table of Contents

Figure S1. The stability of **4** and **5** after treatment with trypsin……………………………S6

Figure S2. Fluorescence microscopic images of Jurkat cells after treatment with **3**, **4**, **5**, **6**, and **7**……………………………………………………………………………………S7

Figure S3. Fluorescence microscopic images of HeLa-S3 cells after treatment with **3**, **4**, **5**, **6**, and **7**……………………………………………………………………………………S8

Figure S4. Fluorescence microscopic images of A549 cells after treatment with **3**, **4**, **5**, **6**, and **7**……………………………………………………………………………………S9

Figure S5. Fluorescence microscopic images of HeLa-S3, and A549 cells after treatment with celastrol……………………………………………………………………………………S10

Figure S6. The results of the MTT assays of HeLa-S3, and A549 cells after treatment with celastrol in the presence of Z-VAD-fmk, necrostatin-1, 3-MA, and CCCP………………S11

Figure S7. Fluorescence microscopic images of Jurkat, HeLa-S3, and A549 cells treated with Rhod-2/AM or Rhod-4/AM in the presence of **4**……………………………………………S12

Figure S8. Time-dependent change of fluorescence emission from Rhod-2/AM or Rhod-4/AM in Jurkat, HeLa-S3, A549, and IMR90 cells after addition of **4** and celastrol……………………………………………………………………………………S14

Figure S9. Fluorescence microscopic images of HeLa-S3 cells stained with Rhod-2/AM or

Rhod-4/AM after addition of celastrol……………………………………………………S15

Figure S10. Fluorescence microscopic images of A549 cells stained with Rhod-2/AM or Rhod-4/AM after addition of celastrol…………………………………………………………S16

Figure S11. Fluorescence microscopic images of HeLa-S3 cells stained with Rhod-2/AM or Rhod-4/AM after addition of cisplatin……………………………………………………S17

Figure S12. Emission intensity profiles of MitoTracker Green and ERTracker Red in Figure 6………………………………………………………………………………………S18

Figure S13. Typical fluorescence confocal microscopy images of HeLa-S3 and A549 cells treated with MitoTracker Green, and ERTracker Red after the treatment with celastrol.… S19

Figure S14. Emission intensity profiles of MitoTracker Green and ERTracker Red in HeLa-S3 and A549 cells obtained from Figure S13…………………………………………………S20

Figure S15. The results of the MTT assay of Jurkat cells treated with **4** in the presence of $Zn(NO_3)_2$. ……………………………………………………………………...S21

Figure S16. Fluorescence microscopic images of Jurkat cells treated with Mito-FerroGreen in the presence of cisplatin, etoposide, **4** and celastrol…………………………………………S22

Figure S17. Fluorescence microscopic images of Jurkat cells treated with zinquin in the presence of cisplatin, etoposide, **4**, and celastrol……………………………………………S23

Figure S18. The results of the MTT assays of Jurkat cells after treatment with **4**, celastrol, and

etoposide in the presence of ATP and ADP...S24

Figure S19. Typical fluorescence confocal microscopy images of IMR90 cells treated with MitoTracker Green, and ERTracker Red in the presence of **4**....................................S25

Figure S20. Emission intensity profiles of MitoTracker Green and ERTracker Red obtained from Figure S19... S26

Figure S21. Fluorescence microscopic images of IMR90 cells treated with Rhod-2/AM or Rhod-4/AM in the presence of **4**..S27

Figure S22. The results of flow cytometry analysis of Jurkat, HeLa-S3, and A549 cells after the treatment with Rhod-2/AM and Rhod-4/AM in the presence of **4** and CCCP...S28

Figure S23. Typical fluorescence confocal microscopy images of Jurkat cells stained with MitoTracker Green, and ERTracker Red after pretreatment with CCCP and then **4** and the emission intensity profiles of MitoTracker Green (green curves) and ERTracker Red (red curves) in Jurkat cells...S29

Figure S24. Fluorescence microscopic images of Jurkat cells treated with DilC1(5) and **4** in the presence of CCCP...S30

Chart S1. The structures of etoposide and cisplatin..S31

Chart S2. The structures of Z-VAD-fmk, necrostatin-1, 3-MA, CCCP and FCCP. S31

Chart S3. The structures of DIDS, 2-APB and RuRed...S31

Chart S4. The structures of Mito-Ferro Green and zinquin ethyl ester.........................S32

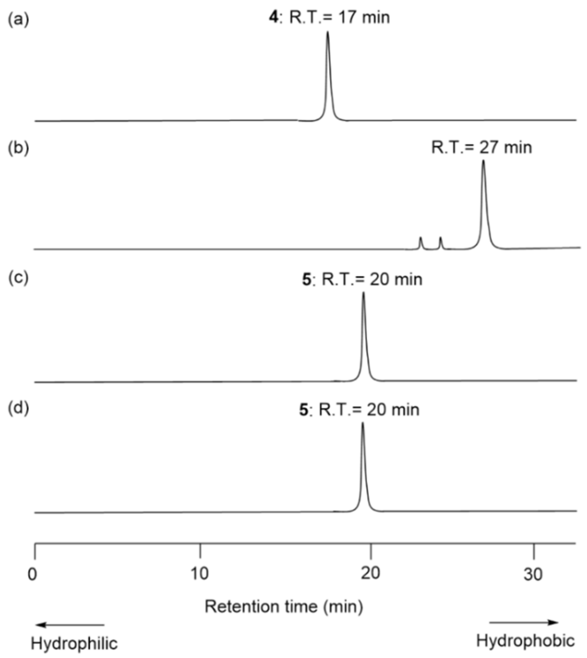

Figure S1. Reversed phase HPLC charts of **4** (10 μM) before treatment with trypsin (a), **4** after treatment with trypsin (5 U/mL) for 1 h at 37 °C (b), **5** (10 μM) before treatment with trypsin (c), and **5** after treatment with trypsin (5 U/mL) for 1 h at 37 °C (d). Elution was carried out with continuous gradient elution (CH_3CN (0.1% TFA)/H_2O (0.1% TFA) = 20/80 to 70/30 (30 min). Column: SenshuPak Pegasil ODS column (4.6 ϕ × 250 mm), flow rate: 1.0 mL/min, UV detection at 220 nm, temperature: room temperature. R.T. depicts retention time.

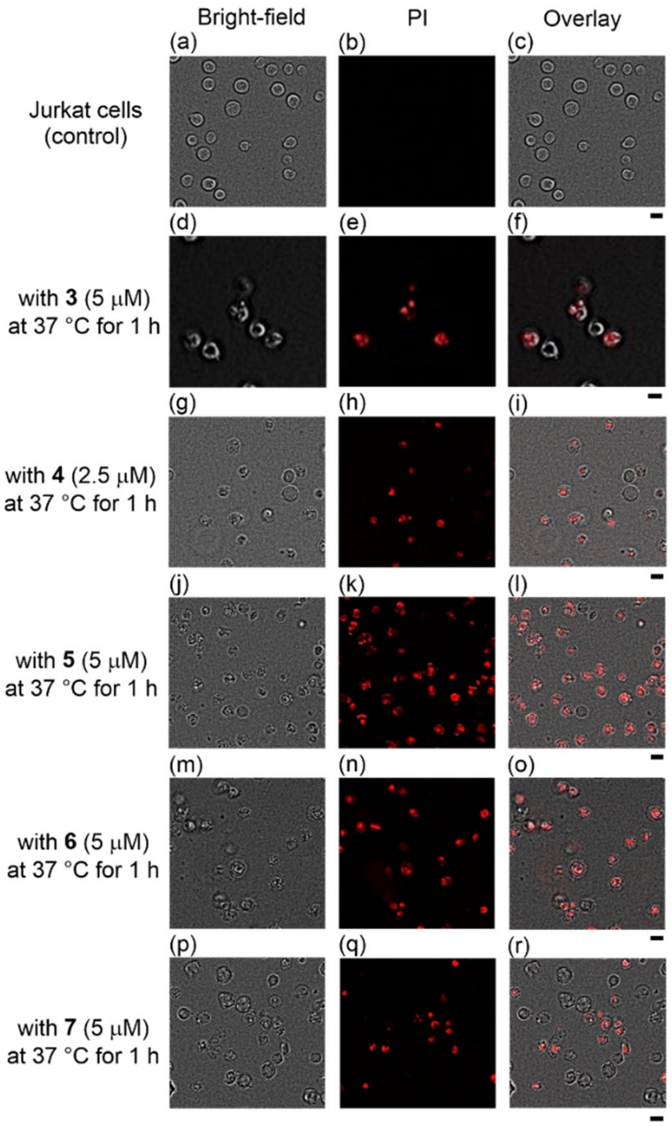

Figure S2. Fluorescence microscopic images of Jurkat cells after treatment with **3** (5 μM), **4** (2.5 μM), **5** (5 μM), **6** (5 μM), and **7** (5 μM) at 37 °C for 1 h. (a) Bright-field, (b) emission, (c) overlay images of the control, (d) bright-field, (e) emission, and (f) overlay images with **3** at 37 °C for 1 h, (g) bright-field, (h) emission, and (i) overlay images with **4** at 37 °C for 1 h, (j) bright field, (k) emission, and (l) overlay images with **5** at 37 °C for 1 h, (m) bright-field, (n) emission, and (o) overlay images with **6** at 37 °C for 1 h, and (p) bright-field, (q) emission, and (r) overlay images with **7** at 37 °C for 1 h. Excitation wavelength was at 540 nm for propidium iodide. Scale bar (black) is 10 μm.

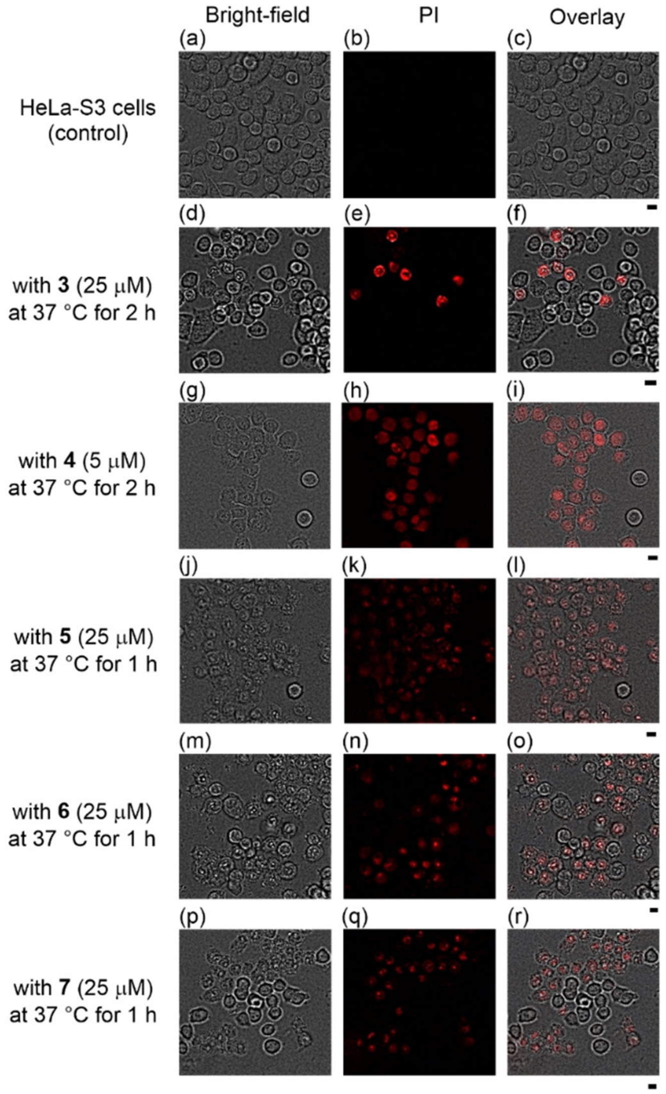

Figure S3. Fluorescence microscopic images of HeLa-S3 cells after treatment with **3** (25 μM), **4** (5 μM), **5** (25 μM), **6** (25 μM), and **7** (25 μM) at 37 °C. (a) Bright-field, (b) emission, (c) overlay images of the control, (d) bright-field, (e) emission, and (f) overlay images with **3** at 37 °C for 2 h, (g) bright-field, (h) emission, and (i) overlay images with **4** at 37 °C for 2 h, (j) bright-field, (k) emission, and (l) overlay images with **5** at 37 °C for 1 h, (m) bright-field, (n) emission, and (o) overlay images with **6** at 37 °C for 1 h, and (p) bright-field, (q) emission, and (r) overlay images with **7** at 37 °C for 1 h. Excitation wavelength was at 540 nm for propidium iodide. Scale bar (black) is 10 μm.

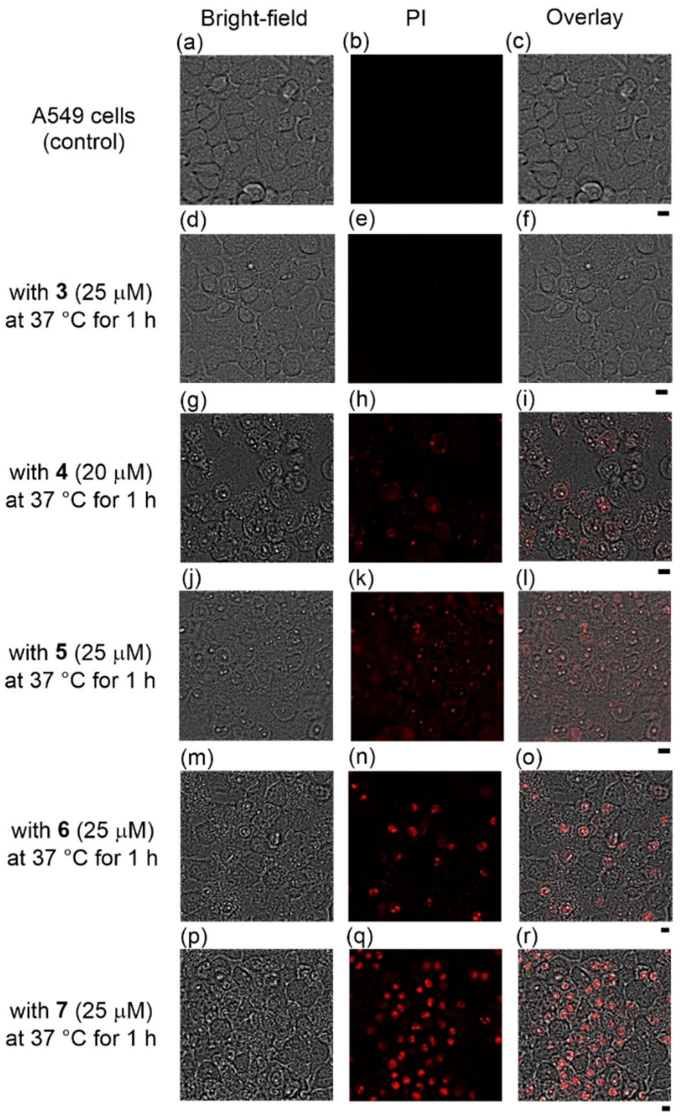

Figure S4. Fluorescence microscopic images of A549 cells after treatment with **3** (25 μM), **4** (20 μM), **5** (25 μM), **6** (25 μM), and **7** (25 μM) at 37 °C for 1 h. (a) Bright-field, (b) emission, (c) overlay images of the control, (d) bright-field, (e) emission, and (f) overlay images with **3** at 37 °C for 1 h, (g) bright-field, (h) emission, and (i) overlay images with **4** at 37 °C for 1 h, (j) bright-field, (k) emission, and (l) overlay images with **5** at 37 °C for 1 h, (m) bright-field, (n) emission, and (o) overlay images with **6** at 37 °C for 1 h, and (p) bright-field, (q) emission, and (r) overlay images with **7** at 37 °C for 1 h. Excitation wavelength was at 540 nm for propidium iodide. Scale bar (black) is 10 μm.

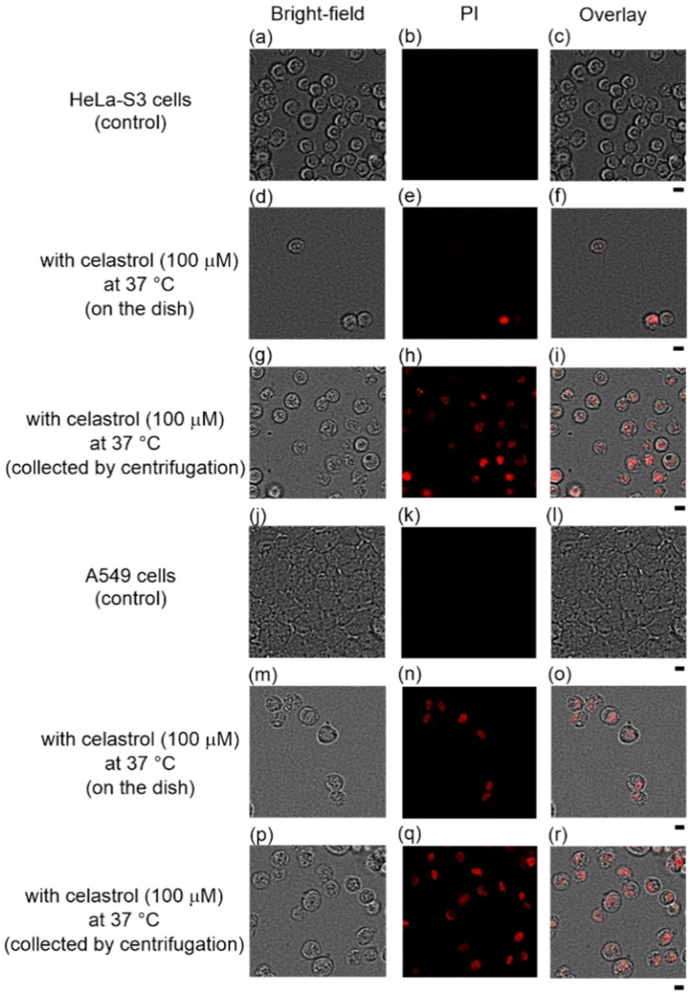

Figure S5. Fluorescence microscopic images of HeLa-S3 (a-i) and A549 cells (j-r) after treatment with celastrol (100 μM) at 37 °C for 24 h. (a) Bright-field image of HeLa-S3 cells, (b) emission, (c) overlay images of the control, (d) bright-field image of HeLa-S3 cells (images on the dish), (e) emission, and (f) overlay images with celastrol at 37 °C for 24 h, (g) bright-field image of HeLa-S3 cells (detached cells were collected by centrifugation), (h) emission, and (i) overlay images with celastrol at 37 °C for 24 h. (j) Bright-field image of A549 cells, (k) emission, (l) overlay images of the control, (m) bright-field image of A549 cells (images on the dish), (n) emission, and (o) overlay images with celastrol at 37 °C for 24 h, (p) bright-field image of A549 cells (detached cells were collected by centrifugation), (q) emission, and (r) overlay images with celastrol at 37 °C for 24 h. Excitation was at 540 nm for propidium iodide. Scale bar (black) is 10 μm.

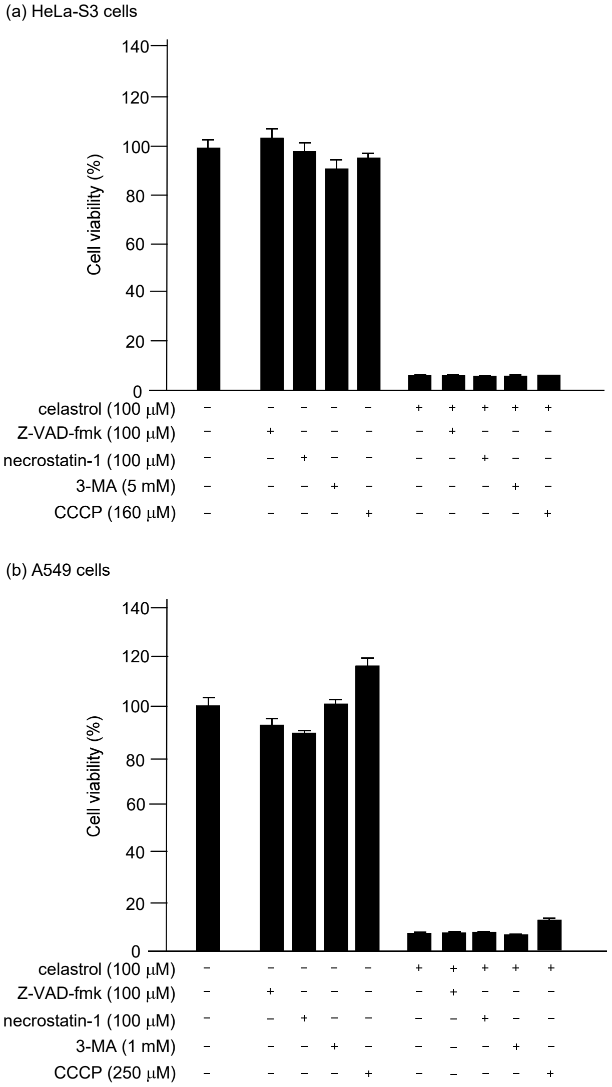

Figure S6. The results of the MTT assays of HeLa-S3 (a), and A549 cells (b) after treatment with celastrol (100 μM) for 24 h in the presence of Z-VAD-fmk, necrostatin-1, 3-MA, and CCCP. (a) Z-VAD-fmk (100 μM), necrostatin-1 (100 μM), 3-MA (5 mM), and CCCP (160 μM), (b) Z-VAD-fmk (100 μM), necrostatin-1 (100 μM), 3-MA (1 mM), and CCCP (250 μM).

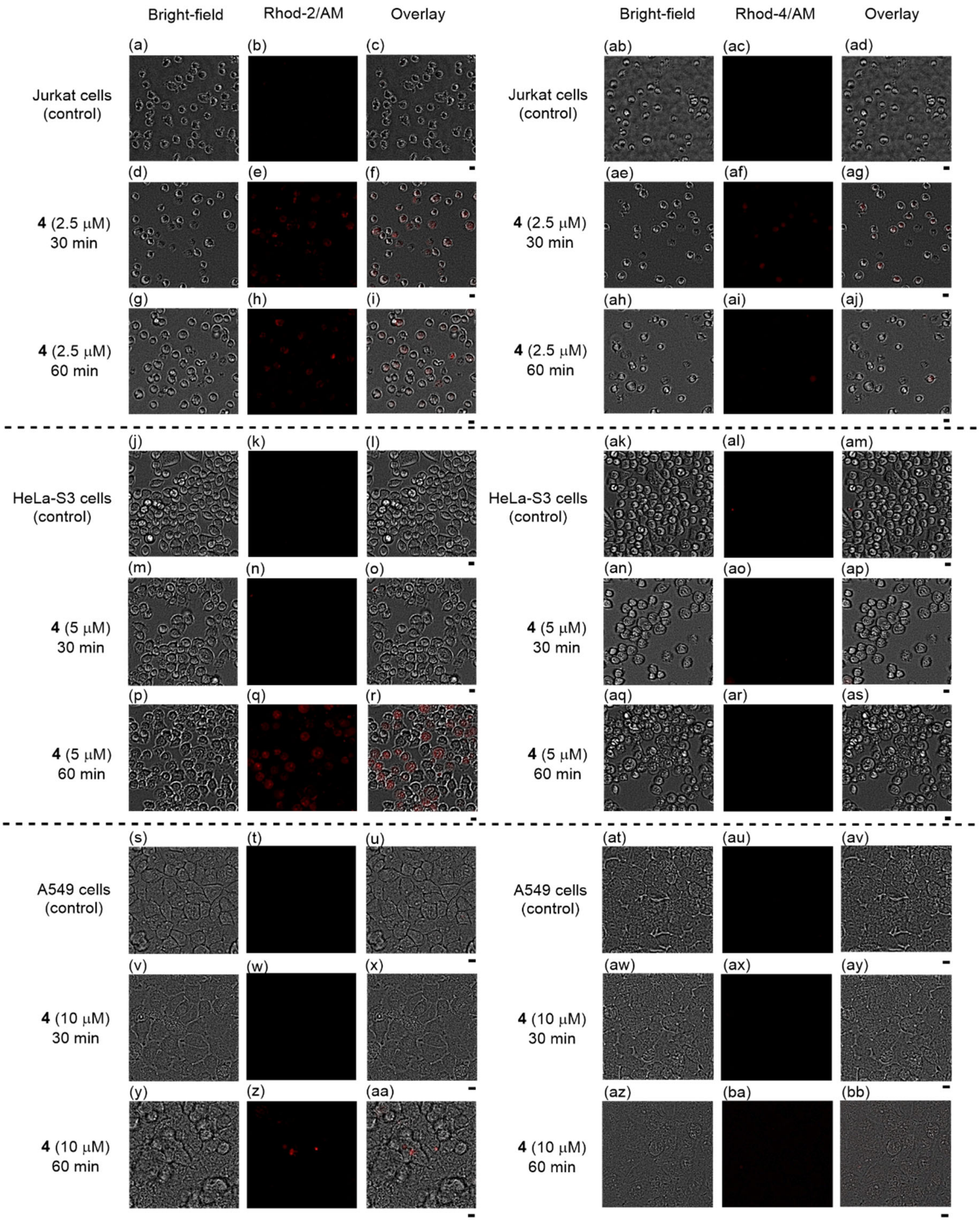

Figure S7. Fluorescence microscopic images of Jurkat, HeLa-S3, and A549 cells treated with Rhod-2/AM (5 μM) or Rhod-4/AM (5 μM) in the presence of **4**. (a), (d), (g), (ab), (ae) and (ah) Bright-field images of Jurkat cells, (b), (e) and (h) emission images of Rhod-2, (ac), (af) and (ai) emission images of Rhod-4, (c) overlay images of (a) and (b), (f) overlay images of (d)

and (e), (i) overlay images of (g) and (h), (ad) overlay images of (ab) and (ac), (ag) overlay images of (ae) and (af), and (aj) overlay images of (ah) and (ai). (j), (m), (p), (ak), (an) and (aq) Bright-field images of HeLa-S3 cells, (k), (n) and (q) emission images of Rhod-2, (al), (ao) and (ar) emission images of Rhod-4, (l) overlay images of (j) and (k), (o) overlay images of (m) and (n), (r) overlay images of (p) and (q), (am) overlay images of (ak) and (al), (ap) overlay images of (an) and (ao), and (as) overlay images of (aq) and (ar). (s), (v), (y), (at), (aw) and (az) Bright-field images of A549 cells, (t), (w), and (z) emission images of Rhod-2, (au), (ax) and (ba) emission images of Rhod-4, (u) overlay images of (s) and (t), (x) overlay images of (v) and (w), (aa) overlay images of (y) and (z), (av) overlay images of (at) and (au), (ay) overlay images of (aw) and (ax), and (bb) overlay images of (az) and (ba). Excitation at 540 nm for (b), (e), (h), (k), (n), (q), (t), (w), (z), (ac), (af), (ai), (al), (ao), (ar), (au), (ax) and (ba). Scale bar (black) is 10 μm.

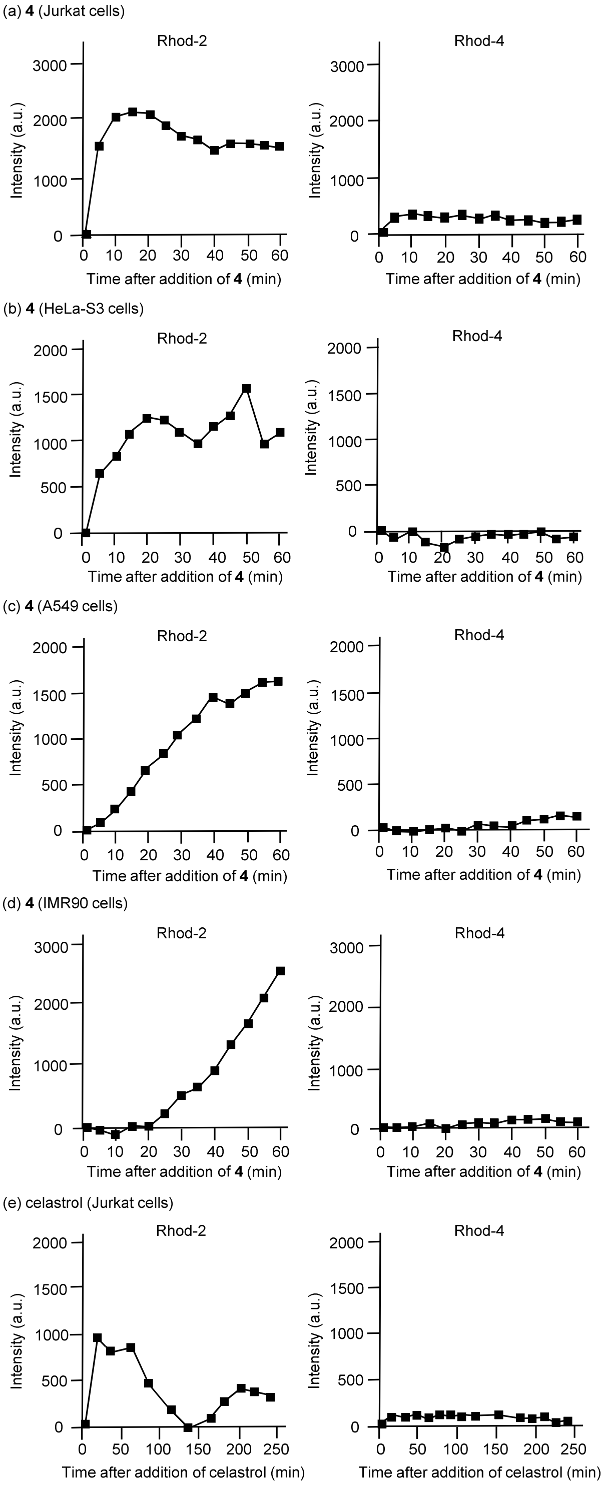

Figure S8. (a-d) Time-dependent change of fluorescence emission from Rhod-2/AM and Rhod-4/AM (excitation at 540 nm and emission at 590 nm) in Jurkat (a), HeLa-S3 (b), A549 (c), and IMR90 cells (d) after addition of **4** (2.5 μM for Jurkat, 5 μM for HeLa-S3, 10 μM for A549, and 5 μM for IMR90 cells) at 37 °C. (e) Time-dependent change of fluorescence emission from Rhod-2/AM and Rhod-4/AM (excitation at 540 nm and emission at 590 nm) in Jurkat cells after addition of celastrol (30 μM) at 37 °C. A.u. is arbitrary unit.

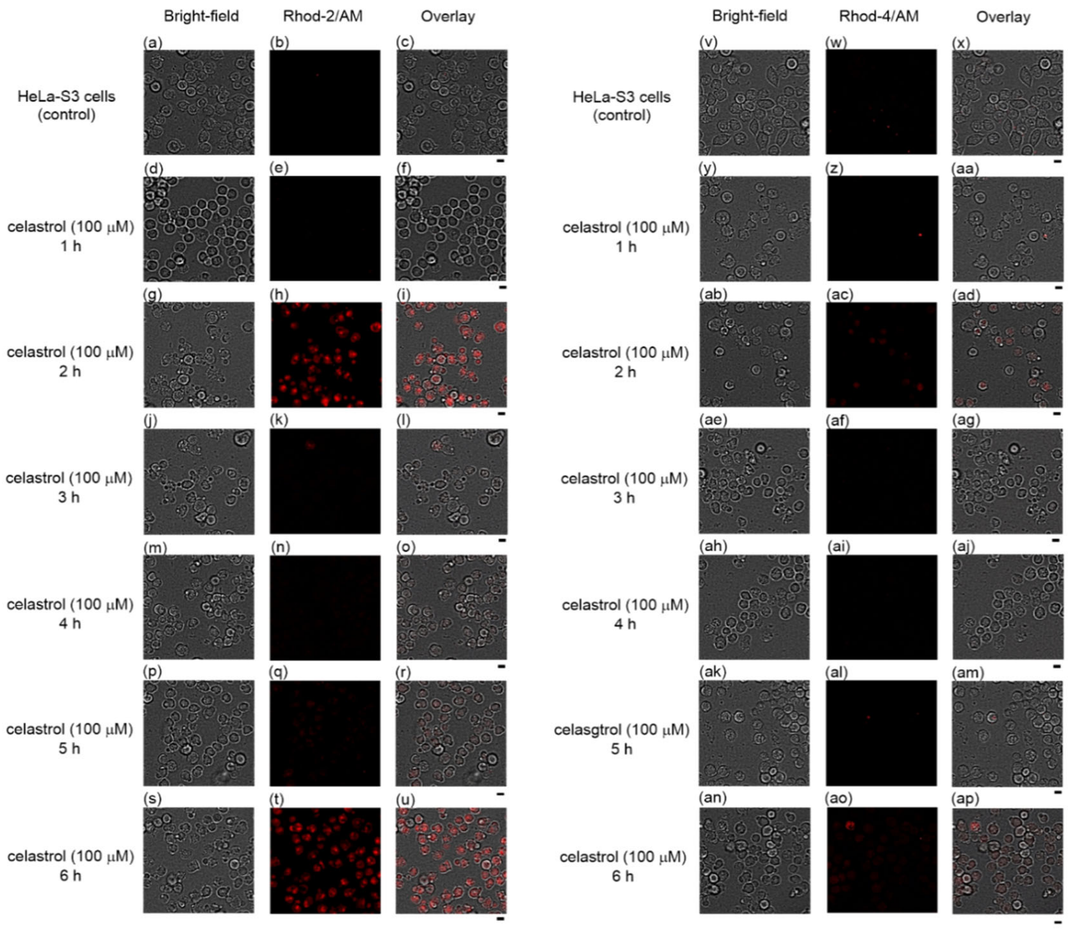

Figure S9. Fluorescence microscopic images of HeLa-S3 cells stained with Rhod-2/AM (5 μM) or Rhod-4/AM (5 μM) after addition of celastrol (100 μM). (a), (d), (g), (j), (m), (p), (s), (v), (y), (ab), (ae), (ah), (ak) and (an) Bright-field images of HeLa-S3 cells, (b), (e) (h), (k), (n), (q) and (t) emission images of Rhod-2, (w), (z), (ac), (af), (ai), (al) and (ao) emission images of Rhod-4, (c) overlay images of (a) and (b), (f) overlay images of (d) and (e), (i) overlay images of (g) and (h), (l) overlay images of (j) and (k), (o) overlay images of (m) and (n), (r) overlay images of (p) and (q), (u) overlay images of (s) and (t), (x) overlay images of (v) and (w), (aa) overlay images of (y) and (z), (ad) overlay images of (ab) and (ac), (ag) overlay images of (ae) and (af), (aj) overlay images of (ah) and (ai), (am) overlay images of (ak) and (al), and (ap) overlay images of (an) and (ao). Excitation at 540 nm for (b), (e), (h), (k), (n), (q), (t), (w), (z), (ac), (af), (ai), (al) and (ao). Scale bar (black) is 10 μm.

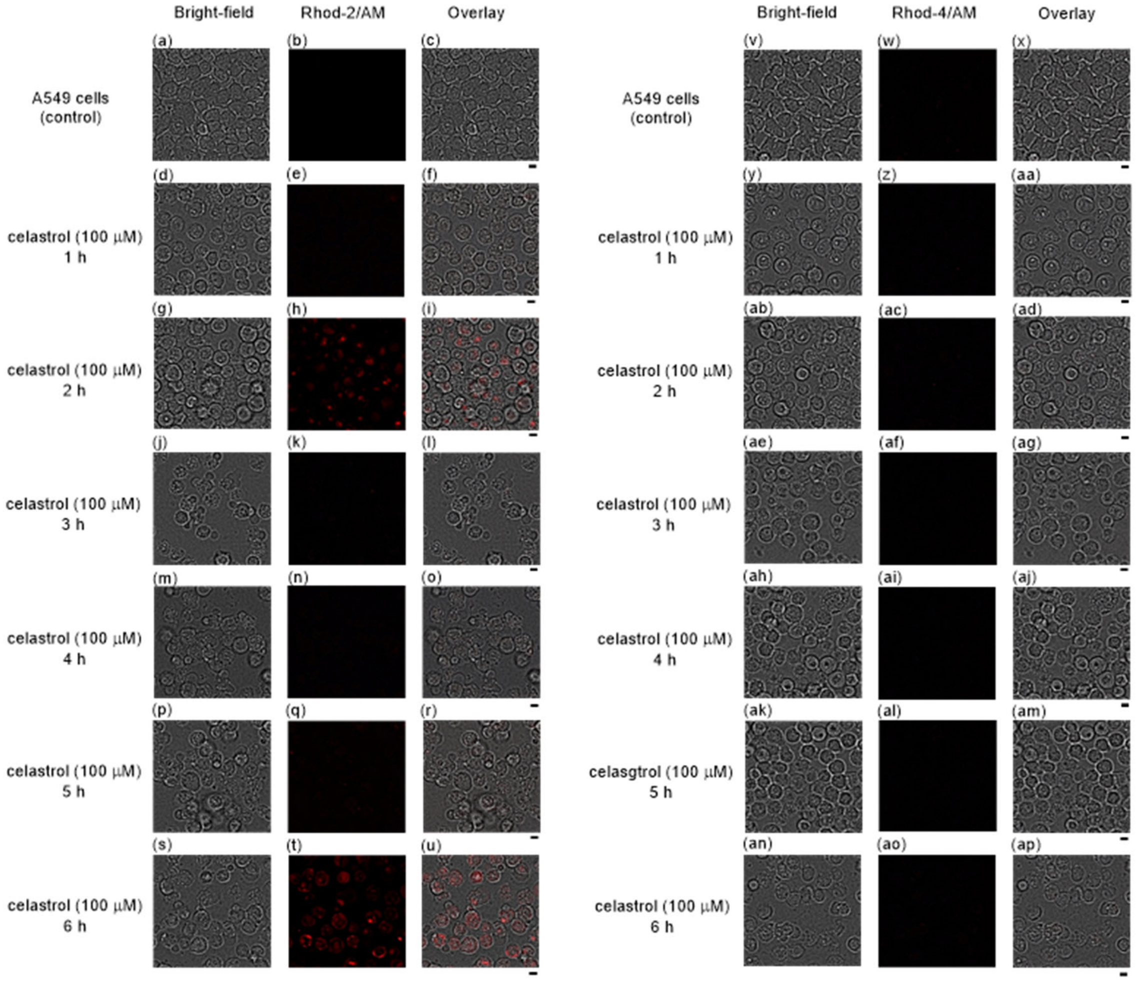

Figure S10. Fluorescence microscopic images of A549 cells stained with Rhod-2/AM (5 μM) or Rhod-4/AM (5 μM) after addition of celastrol (100 μM). (a), (d), (g), (j), (m), (p), (s), (v), (y), (ab), (ae), (ah), (ak) and (an) Bright-field images of A549 cells, (b), (e) (h), (k), (n), (q) and (t) emission images of Rhod-2, (w), (z), (ac), (af), (ai), (al) and (ao) emission images of Rhod-4, (c) overlay images of (a) and (b), (f) overlay images of (d) and (e), (i) overlay images of (g) and (h), (l) overlay images of (j) and (k), (o) overlay images of (m) and (n), (r) overlay images of (p) and (q), (u) overlay images of (s) and (t), (x) overlay images of (v) and (w), (aa) overlay images of (y) and (z), (ad) overlay images of (ab) and (ac), (ag) overlay images of (ae) and (af), (aj) overlay images of (ah) and (ai), (am) overlay images of (ak) and (al), and (ap) overlay images of (an) and (ao). Excitation at 540 nm for (b), (e), (h), (k), (n), (q), (t), (w), (z), (ac), (af), (ai), (al) and (ao). Scale bar (black) is 10 μm.

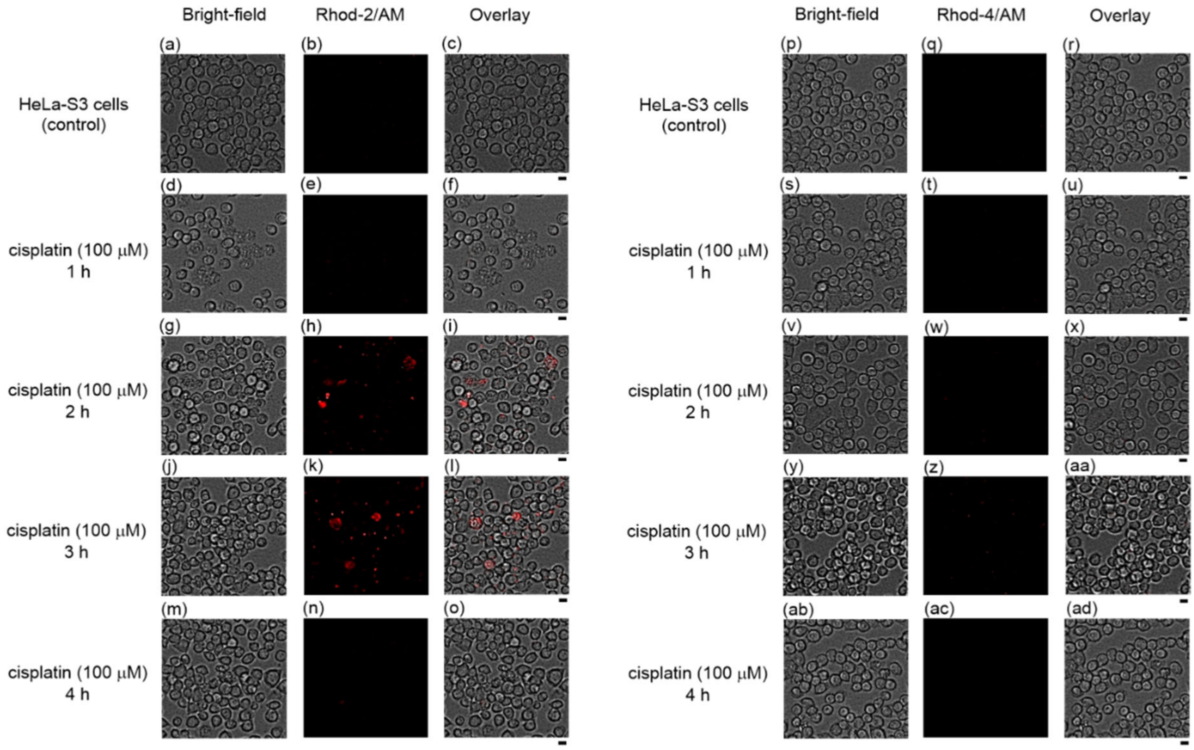

Figure S11. Fluorescence microscopic images of HeLa-S3 cells stained with Rhod-2/AM (5 μM) or Rhod-4/AM (5 μM) after addition of cisplatin (100 μM). (a), (d), (g), (j), (m), (p), (s), (v), (y) and (ab) Bright-field images of HaLa-S3 cells, (b), (e) (h), (k) and (n) emission images of Rhod-2, (q), (t), (w), (z) and (ac) emission images of Rhod-4, (c) overlay images of (a) and (b), (f) overlay images of (d) and (e), (i) overlay images of (g) and (h), (l) overlay images of (j) and (k), (o) overlay images of (m) and (n), (r) overlay images of (p) and (q), (u) overlay images of (s) and (t), (x) overlay images of (v) and (w), (aa) overlay images of (y) and (z), and (ad) overlay images of (ab) and (ac). Excitation at 540 nm for (b), (e), (h), (k), (n), (q), (t), (w), (z) and (ac). Scale bar (black) is 10 μm.

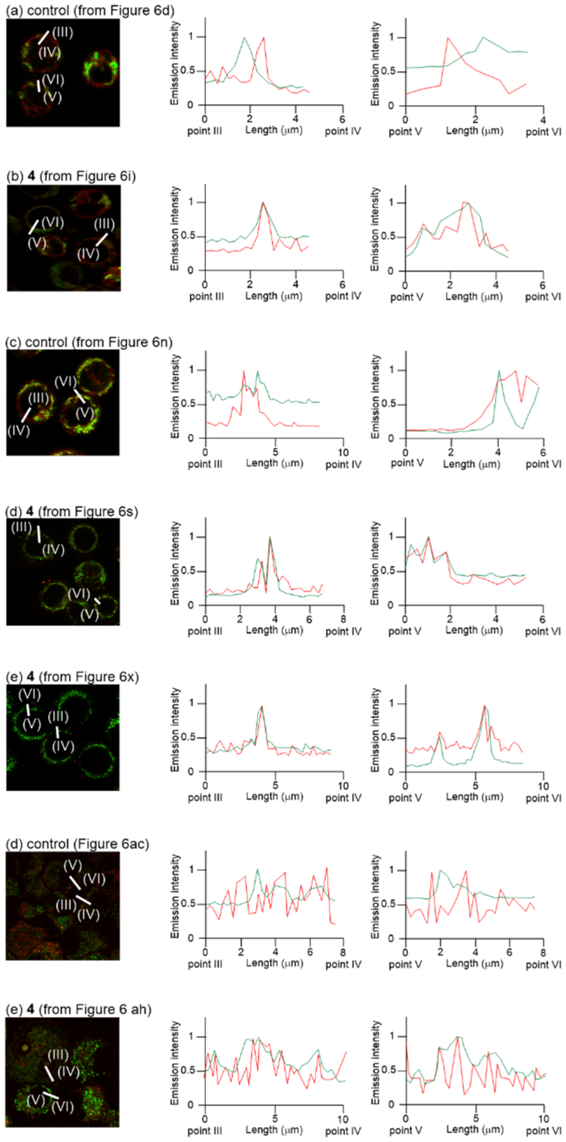

Figure S12. The emission intensity profiles of MitoTracker Green (green curves) and ERTracker Red (red curves) in Jurkat, HeLa-S3, and A549 cells treated with **4**. These plots were taken from the point III to point IV or point V to point VI in Figure S12a for Jurkat cells not treated with **4** (same photo as Figure 6d is used), Figure S12b for Jurkat cells treated with **4** (same photo as Figure 6i is used), Figure S12c for HeLa-S3 cells not treated with **4** (same photo as Figure 6n is used), Figure S12d for HeLa-S3 cells treated with **4** (same photo as Figure 6s is used), Figure S12e (same photo as Figure 6x is used), Figure S12f for A549 cells not treated with **4** (same photo as Figure 6ac is used), and Figure S12g for A549 cells treated with **4** (same photo as Figure 6ah is used).

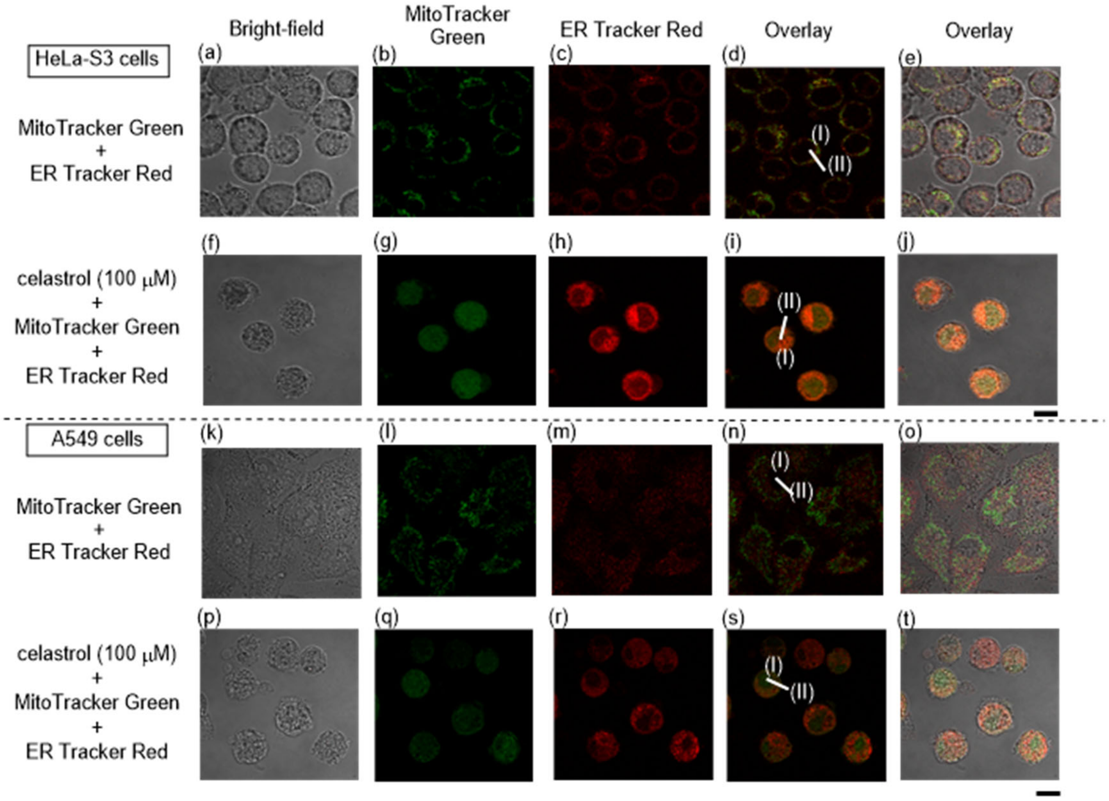

Figure S13. Typical fluorescence confocal microscopy images of HeLa-S3 and A549 cells treated with MitoTracker Green, and ERTracker Red after the treatment with celastrol (100 μM). (a) and (f) Bright-field images of HeLa-S3 cells, (b) and (g) emission images of MitoTracker Green, (c) and (h) emission images of ERTracker Red, (d) overlay images (a-c), (e) overlay images (a-d), (i) overlay images (f-h), and (j) overlay images (f-i). (k) and (p) Bright-field images of A549 cells, (l) and (q) emission images of MitoTracker Green, (m) and (r) emission images of ERTracker Red, (n) overlay images (k-m), (o) overlay images (k-n), (s) overlay images (p-r), and (t) overlay images (p-s). Excitation at 473 nm for (b), (g), (l) and (q), and at 559 nm for (c), (h), (m) and (r). Exposure time was 20 μs/pixel. Scale bar (black) is 10 μm.

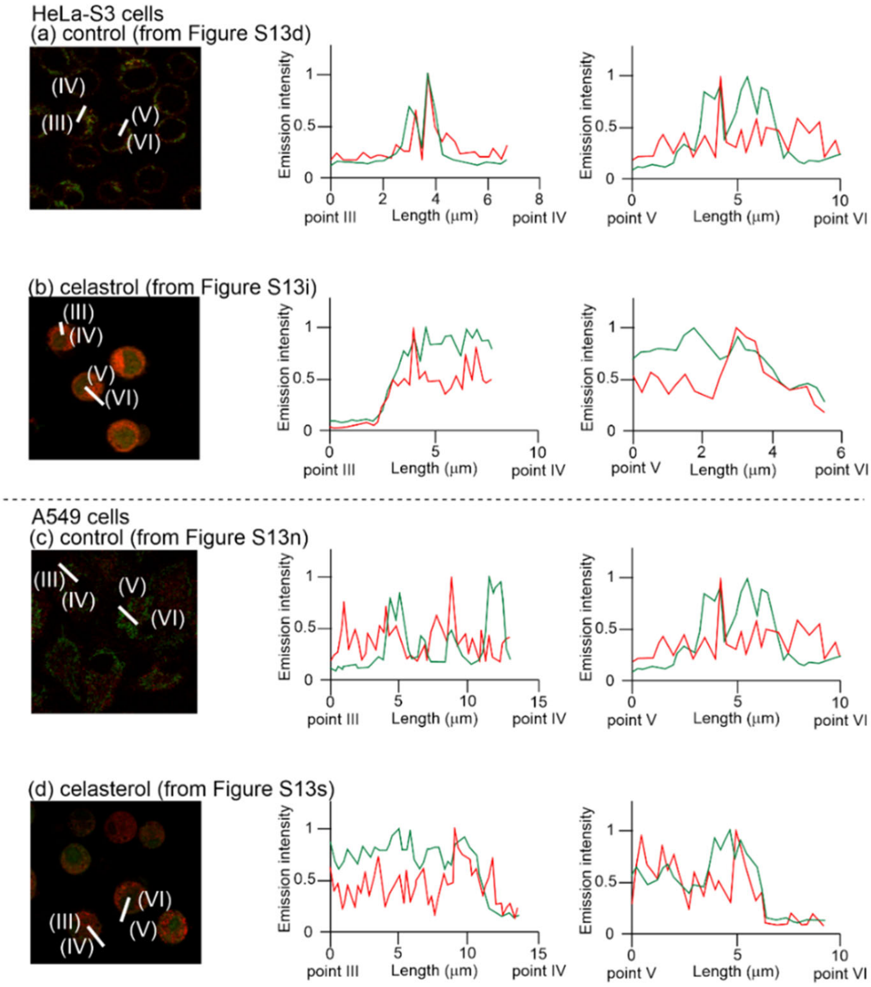

Figure S14. The emission intensity profiles of MitoTracker Green (green curves) and ERTracker Red (red curves) from the point III to point IV or point V to point VI in Figure S14a (same photo as Figure S13d is used), Figure S14b (same photo as Figure S13i is used), Figure S14c (same photo as Figure S13n is used), and Figure S14d (same photo as Figure S13s is used) in HeLa-S3 and A549 cells treated with celastrol.

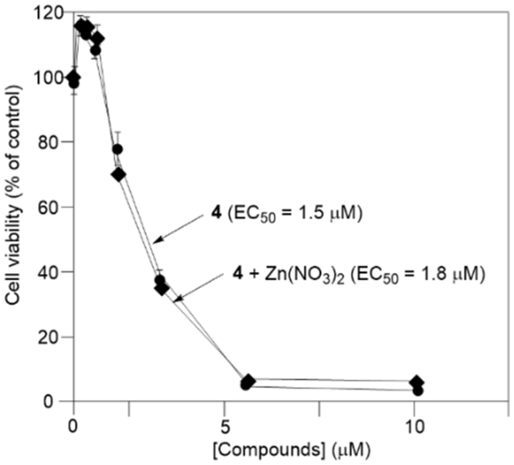

Figure S15. The results of the MTT assay of Jurkat cells treated with **4** alone (closed circles), and in the presence of $Zn(NO_3)_2$ (closed diamonds).

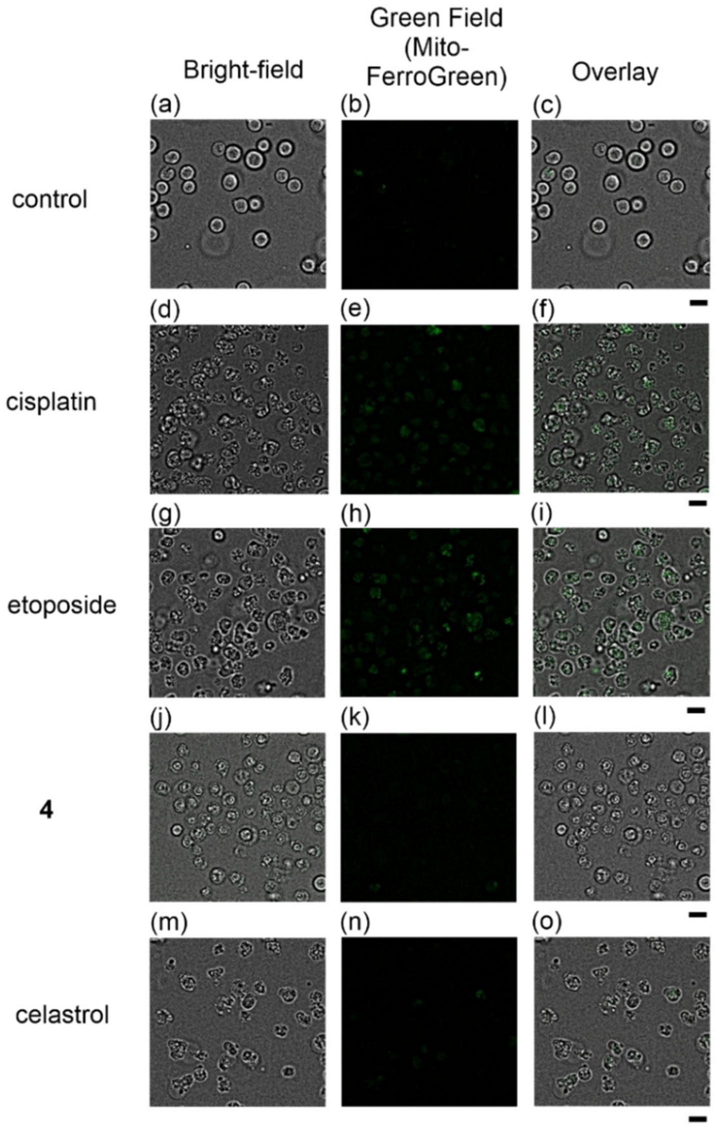

Figure S16. Fluorescence microscopic images of Jurkat cells treated with Mito-FerroGreen to detect Fe^{2+} ions in mitochondria in the presence of cisplatin (100 μM), etoposide (5 μM), **4** (2.5 μM) and celastrol (5 μM) at 37 °C for 1 or 24 h. (a) Bright-field, (b) emission, (c) overlay images of the control, (d) bright-field, (e) emission, (f) overlay images with cisplatin at 37 °C for 24 h, (g) bright-field, (h) emission, and (i) overlay images with etoposide at 37 °C for 24 h, (j) bright-field, (k) emission, and (l) overlay images with **4** at 37 °C for 1 h, and (m) bright-field, (n) emission, and (o) overlay images with celastrol at 37 °C for 24 h. Excitation at 505 nm for Mito-FerroGreen. Scale bar (black) is 10 μm.

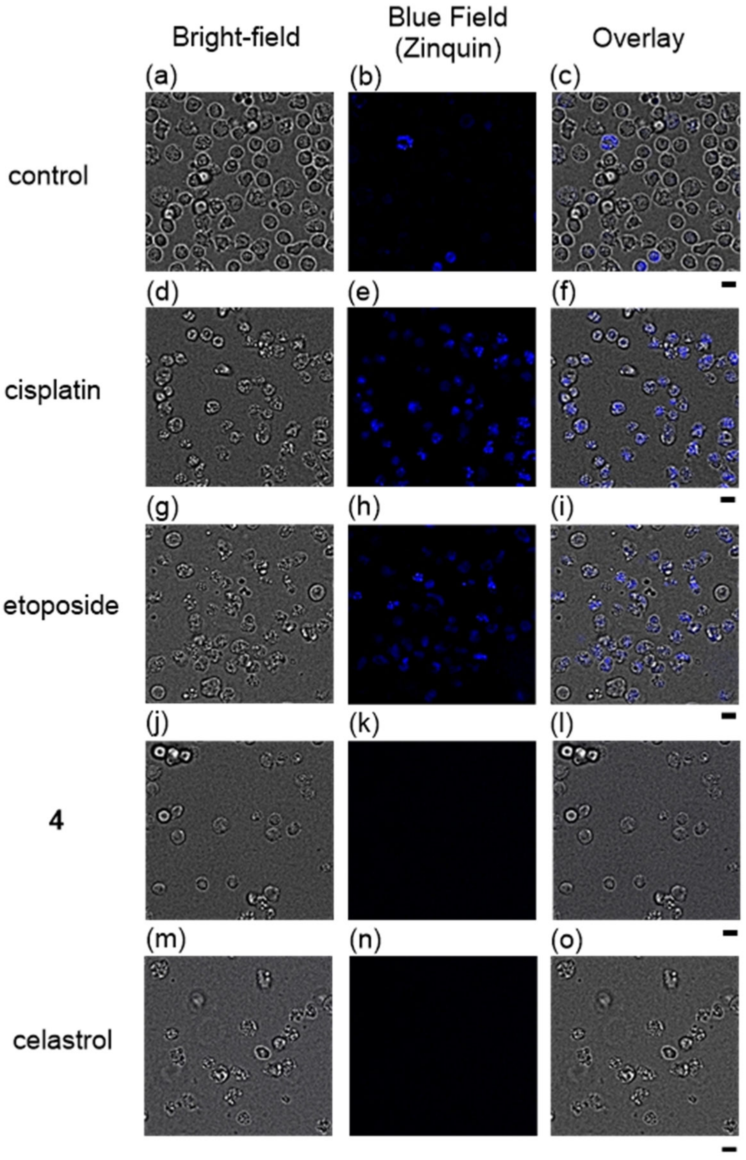

Figure S17. Fluorescence microscopic images of Jurkat cells treated with zinquin to detect intracellular Zn^{2+} ions in the presence of cisplatin (100 μM), etoposide (5 μM), **4** (2.5 μM), and celastrol (5 μM) at 37 °C for 1 or 24 h. (a) Bright-field, (b) emission, and (c) overlay images of the control, (d) bright-field, (e) emission, and (f) overlay images with cisplatin at 37 °C for 24 h, (g) bright-field, (h) emission, and (i) overlay images with etoposide at 37 °C for 24 h, (j) bright-field, (k) emission, and (l) overlay images with **4** at 37 °C for 1 h, and (m) bright-field, (n) emission, and (o) overlay images with celastrol at 37 °C for 24 h. Excitation at 370 nm for zinquin. Scale bar (black) is 10 μm.

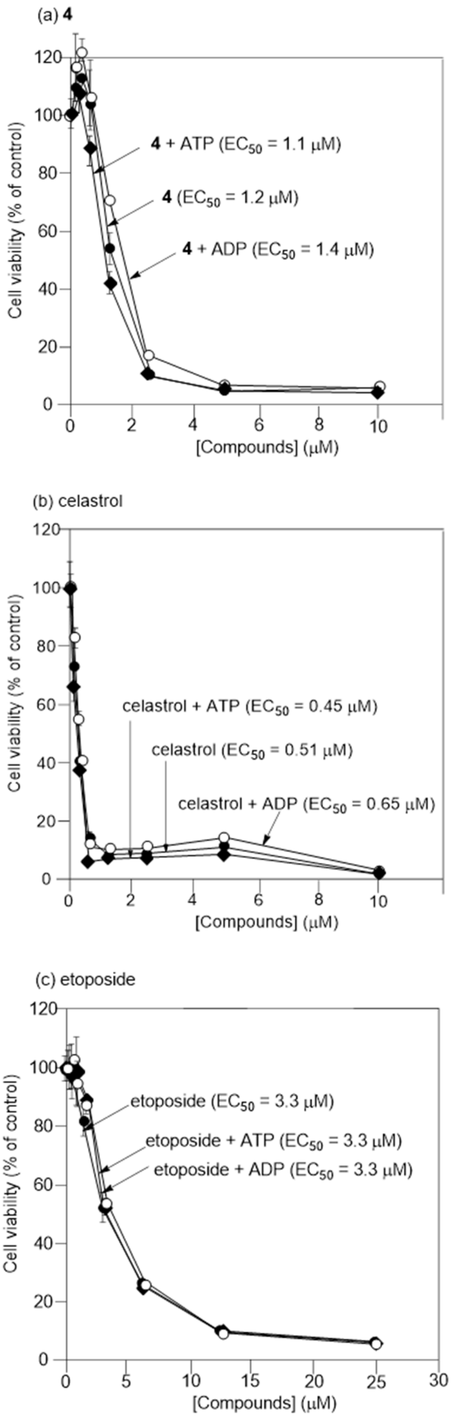

Figure S18. The results of the MTT assays of Jurkat cells after treatment with **4** (a), celastrol (b), and etoposide (c) in the presence of ATP and ADP; compound alone (closed circles), compound+ATP (closed diamond), compound+ADP (opened circles).

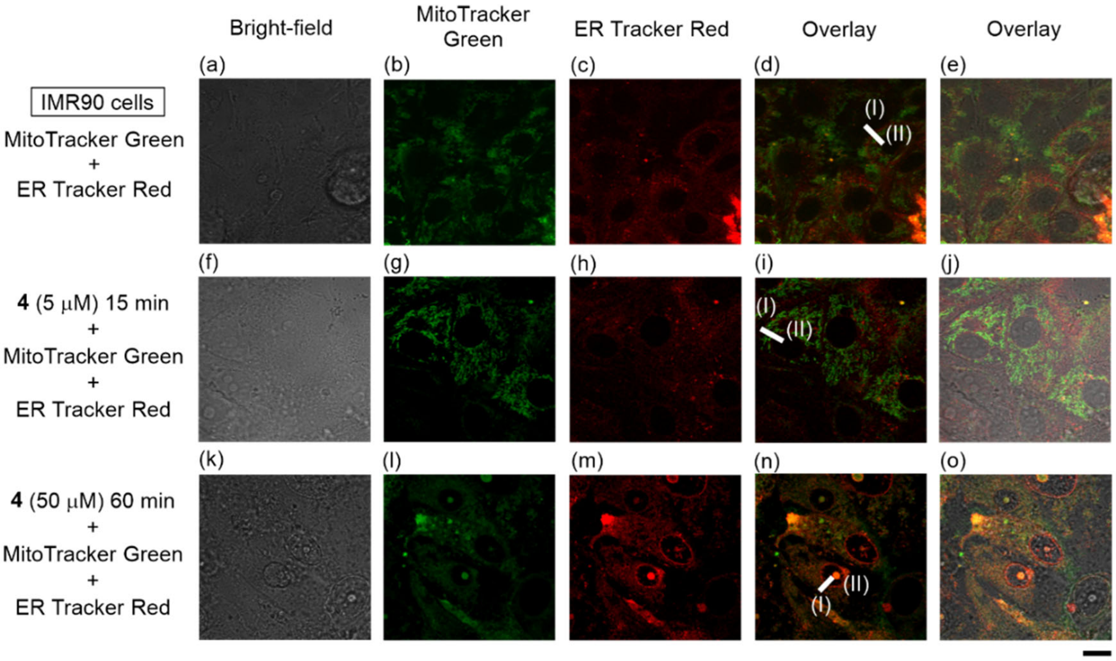

Figure S19. Typical fluorescence confocal microscopy images of IMR90 cells treated with MitoTracker Green, and ERTracker Red in the presence of **4** (5 or 50 μM). (a), (f), and (k) Bright-field images of IMR90 cells, (b), (g) and (l) emission images of MitoTracker Green, (c), (h) and (m) emission images of ERTracker Red, (d) overlay images of (a-c), (e) overlay images of (a-d), (i) overlay images of (f-h), (j) overlay images of (f-i), (n) overlay images of (k-m), and (o) overlay images of (k-n). Excitation at 473 nm for (b), (g) and (l), and at 559 nm for (c), (h) and (m). Exposure time was 20 μs/pixel. Scale bar (black) is 10 μm.

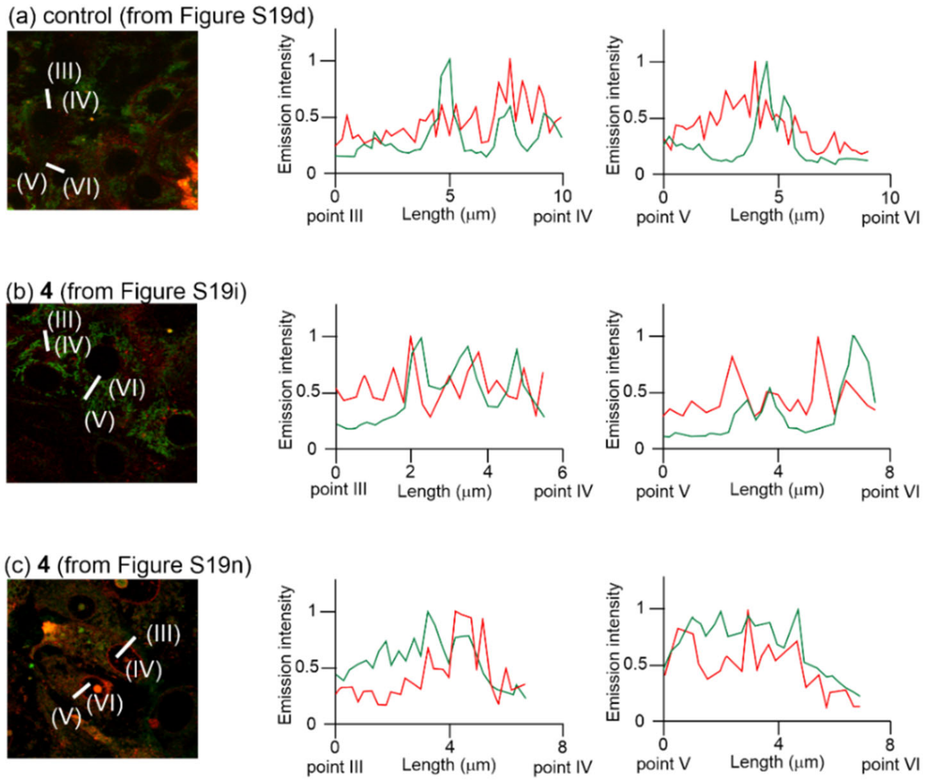

Figure S20. The emission intensity profiles of MitoTracker Green (green curves) and ERTracker Red (red curves) in IMR90 cells obtained from the point III to point IV or point V to point VI in Figure S20a (same photo as Figure S19d is used), Figure S20b (same photo as Figure S19i is used), and Figure S20c (same photo as Figure S19n is used).

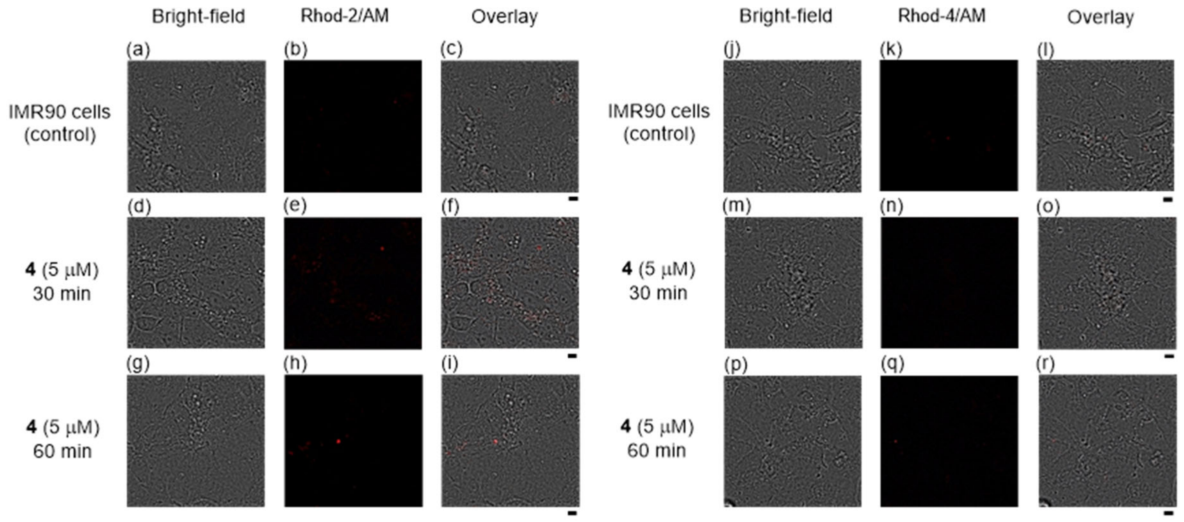

Figure S21. Fluorescence microscopic images of IMR90 cells treated with Rhod-2/AM (5 μM) or Rhod-4/AM (5 μM) in the presence of **4**. (a), (d), (g), (j), (m) and (p) Bright-field images of IMR90 cells, (b), (e) and (h) emission images of Rhod-2, (k), (n) and (q) emission images of Rhod-4, (c) overlay images of (a) and (b), (f) overlay images of (d) and (e), (i) overlay images of (g) and (h), (l) overlay images of (j) and (k), (o) overlay images of (m) and (n), and (r) overlay images of (p) and (q). Excitation at 540 nm for (b), (e), (h), (k), (n) and (q). Scale bar (black) is 10 μm.

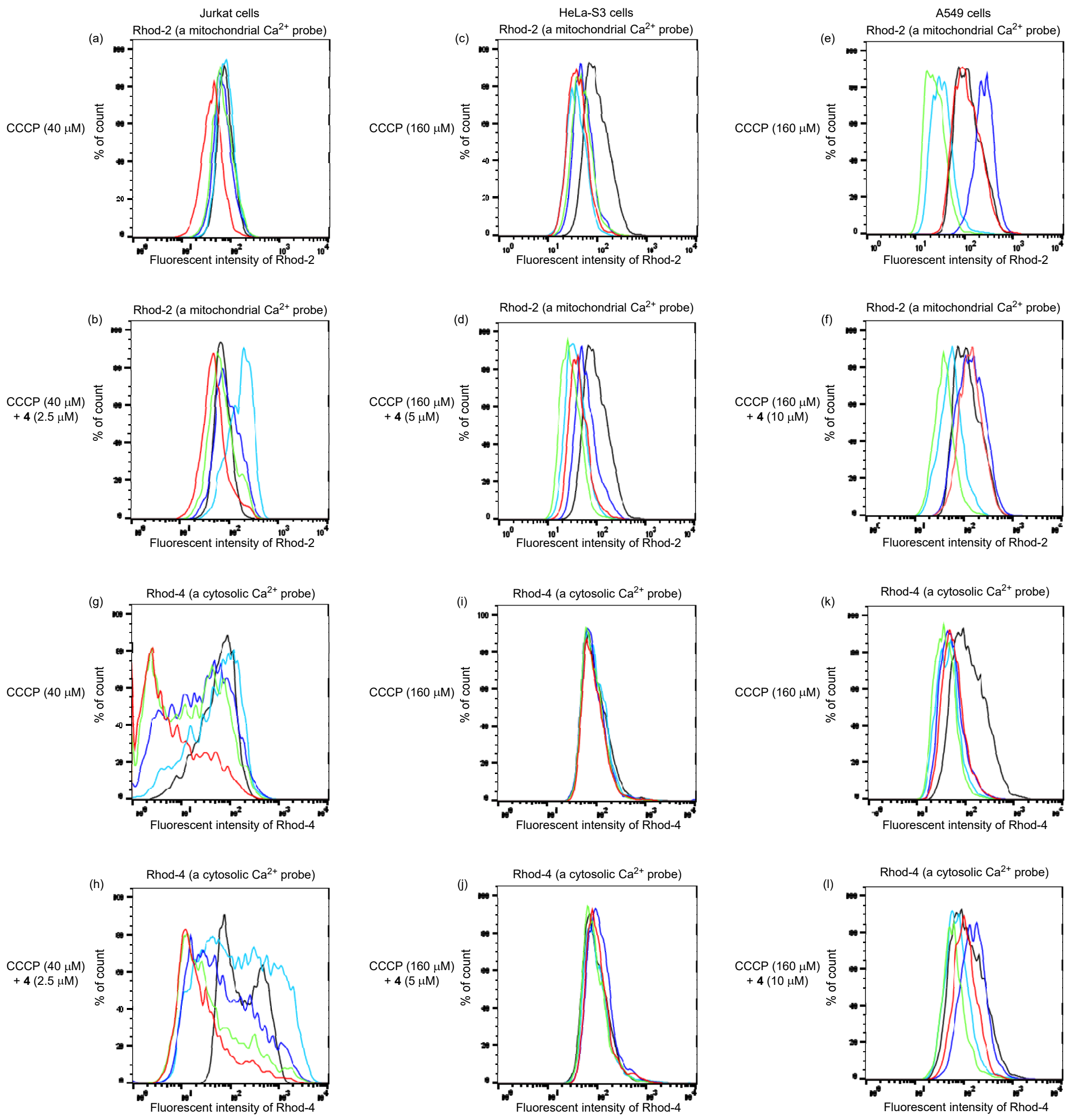

Figure S22. The results of flow cytometry analysis of Jurkat (a, b), HeLa-S3 (c, d), and A549 cells (e, f) after treatment with Rhod-2/AM (5 μM) and **4** (2.5 μM for Jurkat, 5 μM for HeLa-S3, and 10 μM for A549 cells) in the presence of CCCP (40 μM for Jurkat, 160 μM for HeLa-S3 and A549 cells) and those of flow cytometry analysis of Jurkat (g, h), HeLa-S3 (i, j), and A549 cells (k, l) after treatment with Rhod-4/AM (5 μM) and **4** (2.5 μM for Jurkat, 5 μM for HeLa-S3, and 10 μM for A549 cells) in the presence of CCCP (40 μM for Jurkat, 160 μM for HeLa-S3 and A549 cells). Different colors depict different incubation time with **4**: black: 0 min (before addition of **4**), light blue:15 min, green: 45 min, and red: 60 min after addition of **4**.

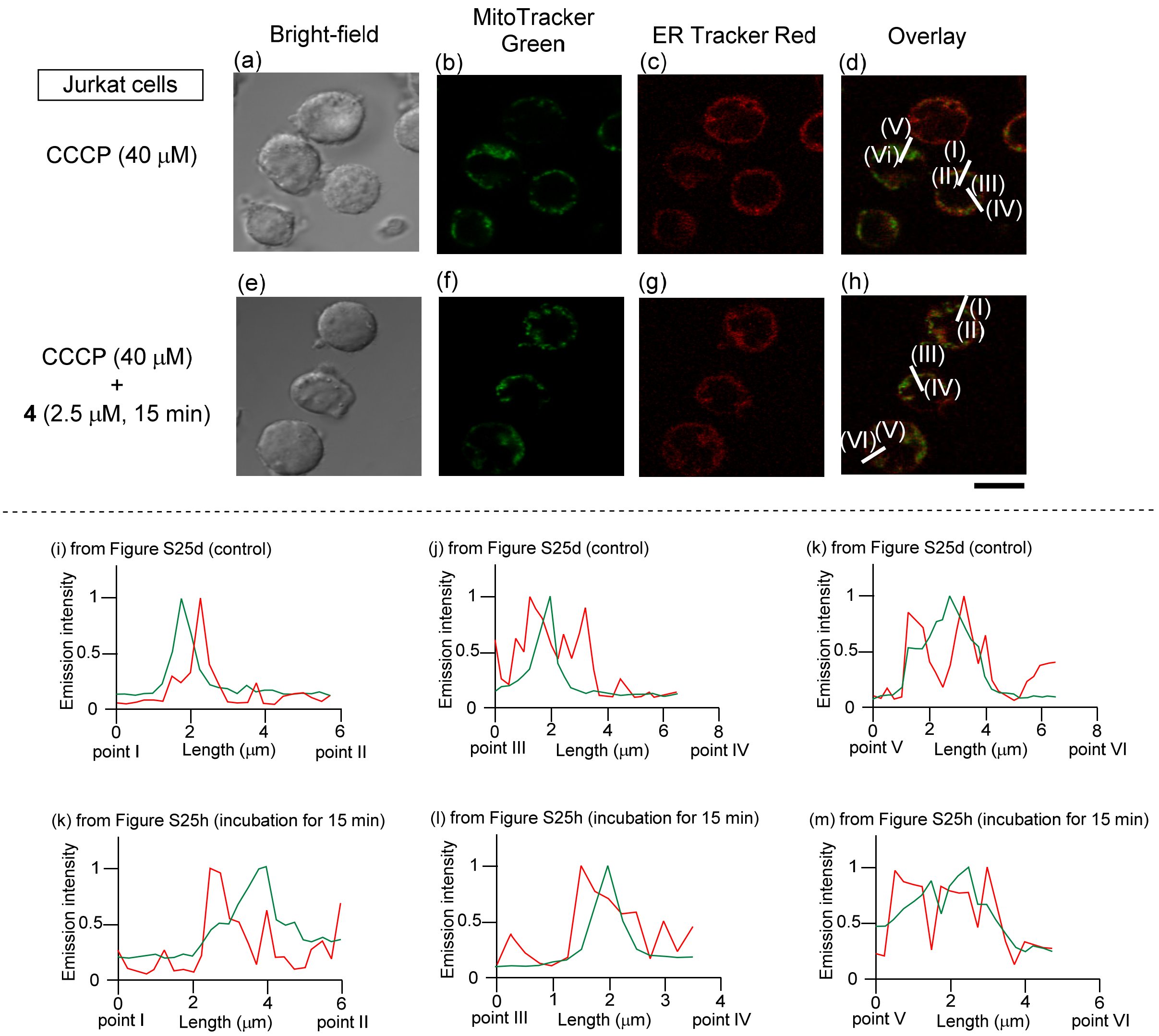

Figure S23. (a-h) Typical fluorescence confocal microscopy images of Jurkat cells stained with MitoTracker Green, and ERTracker Red after pretreatment with CCCP (40 μM) prior to addition of **4** (2.5 μM). (a) and (e) Bright-field images of Jurkat cells, (b) and (f) emission images of MitoTracker Green, (c) and (g) emission images of ERTracker Red, (d) overlay images (a-c), and (h) overlay images (e-g). Excitation at 473 nm for (b) and (f) and at 559 nm for (c) and (g). Exposure time was 20 μs/pixel. Scale bar (black) is 10 μm. (i-m) The emission intensity profiles of MitoTracker Green (green curves) and ERTracker Red (red curves) in Jurkat cells from the point I to point II, point III to point IV, or point V to point VI in Figure S25d, and Figure S25h.

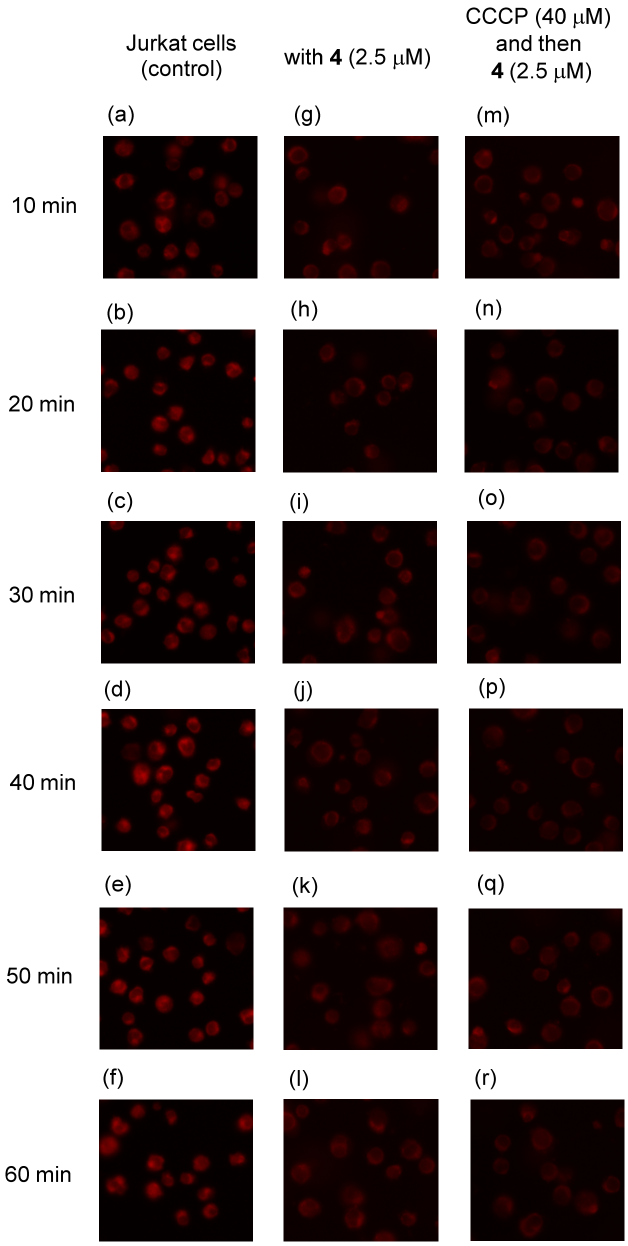

Figure S24. Time-dependent fluorescence microscopic images of Jurkat cells that were stained with DilC1(5) (500 nM) and then treated with **4** in the presence of CCCP. (a-f) Fluorescence images of Jurkat cells stained with DilC1(5) at 10, 20, 30, 40, 50 and 60 min in the absence of **4**, (g-l) fluorescence images of Jurkat cells stained with DilC1(5) at 10, 20, 30, 40, 50 and 60 min after the addition of **4** (2.5 μM), and (m-r) fluorescence images of Jurkat cells stained with DilC1(5) at 10, 20, 30, 40, 50 and 60 min after the treatment with CCCP (40 μM) and then with **4** (2.5 μM). Excitation wavelength for DilC1(5) is 635 nm. Scale bar (black) is 10 μm.

Chart S1. The structures of etoposide and cisplatin.

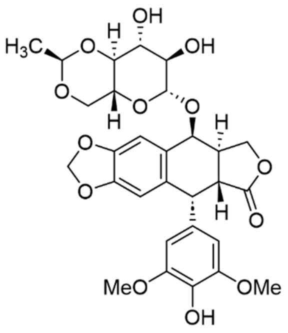

etoposide

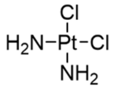

cisplatin

Chart S2. The structures of Z-VAD-fmk, necrostatin-1, 3-MA, CCCP, and FCCP.

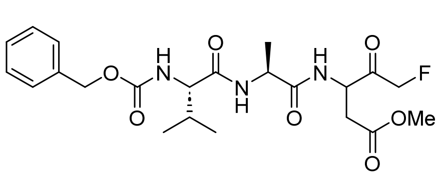

benzyloxycarbonyl-VAD(OMe)-fluoromethylketone
(Z-VAD-fmk)

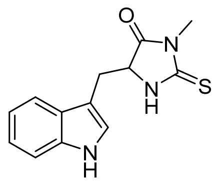

necrostatin-1

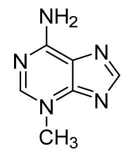

3-methyladenine
(3-MA)

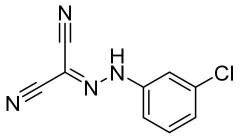

3-chlorophenylhydrazone
(CCCP)

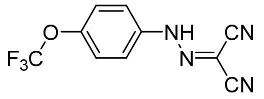

trifluoromethoxy carbonylcyanide phenylhydrazone
(FCCP)

Chart S3. The structures of DIDS, 2-APB and RuRed.

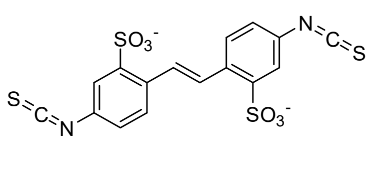

4,4'-diisothiocyano-2,2'-stilbenedisulfonic acid
(DIDS^{2-})

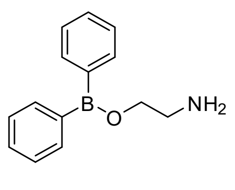

2-aminophenyl borate
(2-APB)

$[Ru(NH_3)_6\text{-O-}Ru(NH_3)_4\text{-O-}Ru(NH_3)_6]^{6+}\ 6Cl^-$

Ruthenium Red

Chart S4. The structures of Mito-FerroGreen and Zinquin ethyl ester.

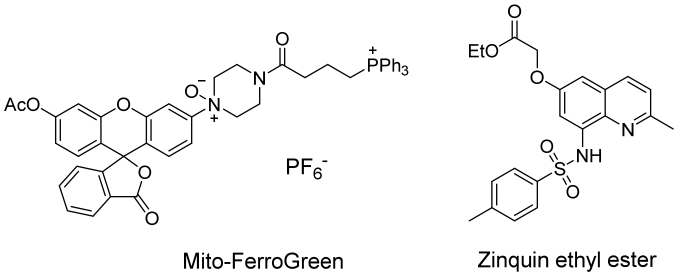

Supplement: Supplementary file 1 — bi4c00085_si_001.pdf [file bi4c00085_si_001.pdf]
